# Glucosidase alpha neutral C promotes influenza virus replication by inhibiting proteosome-dependent degradation of hemagglutinin

**DOI:** 10.1038/s41392-025-02227-6

**Published:** 2025-04-23

**Authors:** Xinzhong Liao, Qian Xie, Minqi Liang, Qijun Liao, Bi Huang, Shengze Zhang, Feng Zhang, Liangliang Wang, Lifang Yuan, Xuejie Liu, Simin Wen, Chuming Luo, Dayan Wang, Yongkun Chen, Huanle Luo, Yuelong Shu

**Affiliations:** 1 School of Public Health (Shenzhen), Shenzhen Key Laboratory of Pathogenic Microbes and Biosafety, Shenzhen Campus of Sun Yat-sen University, Shenzhen, China; 2 Guangzhou First People’s Hospital, Guangzhou Medical University, Guangzhou, China; 3 Chinese National Influenza Center, National Institute for Viral Disease Control and Prevention, Chinese Center for Disease Control and Prevention, Beijing, China; 4 Guangdong Provincial Key Laboratory of Infection Immunity and Inflammation, School of Basic Medical Sciences, Shenzhen University Medical School, Shenzhen University, Shenzhen, China; 5 Key Laboratory of Pathogen Infection Prevention and Control (MOE), State Key Laboratory of Respiratory Health and Multimorbidity, National Institute of Pathogen Biology, Chinese Academy of Medical Sciences & Peking Union Medical College, Beijing, China

**Keywords:** Cell biology, Microbiology

## Abstract

The H7N9 influenza virus poses a significant threat to human health, and the mechanism by which it infects humans remains incompletely understood. Our investigation has unveiled significant insights into the role of *glucosidase alpha, neutral C* (*GANC*) gene in human H7N9 infections. Through whole genome sequencing (WGS), we identified five low-frequency functional and heterozygous variants of *GANC* strongly associated with human H7N9 infections compared to healthy controls. Furthermore, we observed a reduction in mRNA and protein expression of GANC following H7N9 virus infection in vitro and in vivo. Subsequent experiments involving GANC demonstrated the promotion of H7N9 virus replication in a stable strain with GANC overexpression. Conversely, GANC knockdown exhibited the ability to restrict influenza A virus (IAV) replication, including H7N9, H9N2, and H1N1, both in vitro and in vivo. This inhibition was mediated by GANC’s ability to promote the degradation of H7N9 hemagglutinin (HA). Moreover, we discovered that GANC knockdown facilitated the degradation of HA in a proteasome-dependent manner. The inhibition caused by GANC knockdown was mediated by promoting direct binding of HA with the proteasome 26S subunit, non-ATPase, 1 (PSMD1) and PSMD2. All five variants in the *GANC* gene reduced their ability to promote H7N9 virus replication, and also diminished the levels of GANC-induced HA protein expression. Our findings revealed a novel mechanism by which GANC inhibits the proteasome-dependent degradation of HA to promote H7N9 virus replication. These results suggest that GANC may play an important role in IAV replication.

## Introduction

The zoonotic H7N9 influenza virus first emerged in Shanghai and Anhui provinces in 2013, causing severe lower respiratory tract infections.^1^ Since then, five epidemic waves have occurred, resulting in 1568 human cases, with 615 fatalities reported.^2,3^ No human cases have been reported since 2019, primarily attributed to widespread vaccination of chicken populations.^4^ The H7N9 virus has acquired several mutations to improve its adaptation to mammals, including alterations in viral hemagglutinin (HA) receptor specificity and polymerase activity.^5^ However, the host genetic factors responsible for the cross-species transmission of the H7N9 virus remain poorly understood. Increasing evidence suggests that host genetic factors play a significant role in determining susceptibility to influenza virus infections. For influenza A virus (IAV), Zhu et al. identified several significant candidate genes through multiple tissue transcriptome-wide association studies. Additionally, the single-cell genome-wide association revealed that a nonsynonymous variant in *endoplasmic reticulum aminopeptidase 1* (*ERAP1*) increases susceptibility to IAV infection.^6^ To identify genetic mutations that may enhance host susceptibility to H7N9 infection, Chen et al. conducted exon sequencing and validated the single-nucleotide polymorphism (SNP) using Sanger sequencing in H7N9 patients. They identified 21 genes highly associated with H7N9 influenza infection, such as DNA methyltransferase 1 (DNMT1), carboxylesterase 1 (CES1), and toll-like receptor 4 (TLR4).^7^ In our previous study, we identified rare and heterozygous single-nucleotide variants (SNVs) in the *MX1* gene, demonstrating a significant association with human H7N9 infections compared to healthy controls through whole-genome sequencing (WGS).^8^ Myxovirus resistance protein A (MxA) is an antiviral factor against a broad spectrum of viruses, including IAV.^9^ However, most of the MxA variants lost the ability to inhibit H7N9 virus replication.^8^

The glucosidase alpha, neutral C (GANC) belongs to the subgroup I of the glycoside hydrolase 31 family.^10^ The human GANC protein is encoded by the *GANC* gene, located on chromosome 15.^11^ According to data from the Human Protein Atlas, the human *GANC* gene is expressed in the majority of human tissues, and has also been observed in cells associated with the human respiratory tract, such as HBEC3-KT, SCLC-21H and A549 cells.^12,13^ Immunofluorescence analysis in various cell lines suggests that GANC is localized to the actin filaments of the cytoskeleton and the nucleoplasm, excluding the nucleoli.^14,15^ It has been reported that GANC can release glucose from glycogen and low molecular-weight substrates containing α-1,4-glycosidic linkages at neutral pH in vitro.^16,17^ Through WGS, we further identified a robust association of the *GANC* gene with human H7N9 infections based on WGS data. However, whether GANC could affect H7N9 virus replication remains unclear.

Most enveloped viruses, including IAV, depend on the endoplasmic reticulum (ER) glycoprotein quality control (QC) machinery for correct glycoprotein folding. Inhibiting this machinery results in misfolded viral glycoproteins, affecting the formation of virions.^18–20^ ER α-glucosidases I and II are critical components of the ER QC machinery.^21,22^ Glucosidase II catalyzes the hydrolysis of the middle and innermost glucose residues of peptide-bound oligosaccharides, initiating glycoprotein folding through the calnexin and calreticulin cycle.^23–25^ Failure of glucosidase II to release glucose from the glycan, may result in glycoprotein misfolding, leading to degradation through intracellular degradation mechanisms. The 26S proteasome predominantly catalyzes protein degradation, including the degradation of misfolded proteins.^26^ Ubiquitination, a widespread post-translational modification of proteins, is an enzymatic process in which ubiquitin molecules are covalently attached to substrate proteins through the coordinated action of E1 ubiquitin-activating enzymes, E2 ubiquitin-conjugating enzymes, and E3 ubiquitin ligases.^27^ In the ubiquitin-proteasome system, proteins containing ubiquitin-binding UBA domains are recognized by ubiquitin receptors on the proteasome. The proteasome 26S subunit, non-ATPase, 1 (PSMD1) and PSMD2 have been reported to bind both ubiquitin proteins and ubiquitin-like proteins.^28–30^ The ubiquitin-proteasome system plays a significant role in IAV infection with host proteins mediating the degradation of IAV proteins. Tripartite motif containing 22 (TRIM22) and TRIM32 promote polyubiquitination of the polymerase basic protein 1 (PB1) and nucleocapsid protein (NP) for degradation.^31,32^ Moreover, PSMD12 mediates polyubiquitination of the matrix protein 1 (M1), leading to its degradation.^33^ The glucosidase II alpha subunit (GANAB) protein, also known as glucosidase II, composed of a catalytic α subunit (GIIα) and a noncatalytic regulatory β subunit (GIIβ), is crucial for QC and maturation of glycoproteins in the ER.^19,34^ Phylogenetic analysis indicated that GANC evolved in early vertebrates from the GIIα of GANAB.^15^ Influenza virus HA protein is classified as a type I glycoprotein.^35^ However, the role of GANC in HA protein degradation remains unclear.

In this study, we confirmed that E3 ubiquitin ligases STIP1 homology and U-box containing protein 1 (STUB1) mediated the degradation of H7N9 HA proteins through the ubiquitin-proteasome system to decrease H7N9 virus replication. In addition, we observed that PSMD1 and PSMD2 also facilitated H7N9 HA degradation to restrict H7N9 virus replication. We first identified the association of the *GANC* gene with human H7N9 infections through WGS. Our study investigates the mechanism by which GANC promotes H7N9 virus replication, revealing its role in inhibiting the proteasome-dependent degradation of HA. Therefore, small molecules inhibiting GANC expression and proteasome inhibitors may provide effective therapeutic strategies for the treatment of H7N9 infectious diseases.

## Results

### Association between low-frequency functional variants of *GANC* and human H7N9 virus infection

**Fig. 1:**
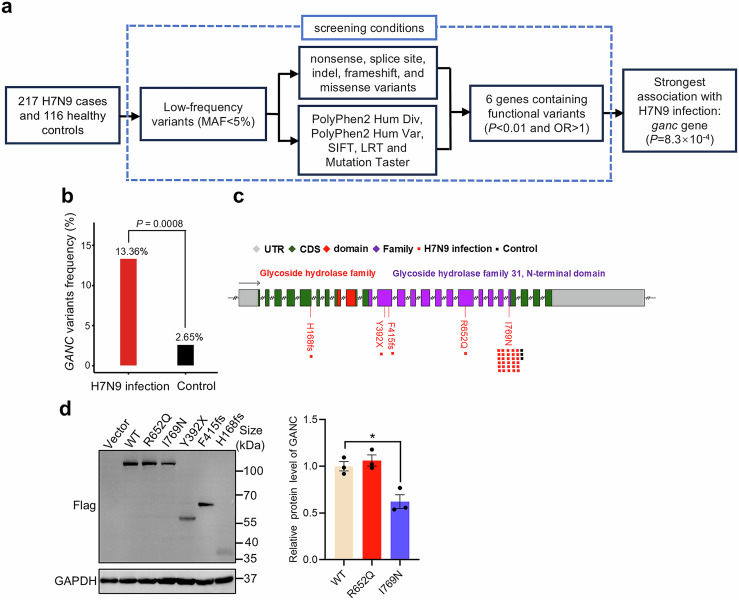
Genetic mutations in the GANC gene are associated with H7N9 infection. **a** Flow chart of the study design. **b** The frequency of the *GANC* variants found in H7N9-infected patients. **c** Identification of five low-frequency functional variants within the *GANC* gene. **d** Western blotting analysis for HEK293T cells transfected with plasmids with GANC and its mutations tagged with Flag for 48 h. Quantification was shown as mean ± SEM. *n* = 3 independent experiments. The Student’s *t*-test (unpaired, two-tailed) was used to compare two independent groups. *P < 0.05

To identify host genes associated with human susceptibility to H7N9 infections, we conducted WGS on individuals with human H7N9 infections and healthy controls. In our previous study, we identified that the rare mutations of the *MX1* gene were linked to susceptibility to H7N9 virus infection, based on a cohort of 217 H7N9 infections and 116 healthy controls.^8^ In this study, we conducted a gene-based association analysis using collapse methods, and focused on low-frequency variants with a minor allele frequency (MAF) of less than 5%, based on predictions of their damaging effects using five distinct prediction algorithms. We primarily focused on genes where low-frequency variants showed a higher prevalence in patients, indicated by an odds ratio (OR) greater than 1 (Fig. 1a). In this study, based on gene-based association analysis, we observed the frequency of *GANC* variants to be 13.36% in H7N9 infections, significantly higher than 2.65% observed in controls. This discrepancy indicated a significant enrichment of *GANC* variants in H7N9 infections (*P* = 0.0008; OR, 5.81; 95% confidence interval [CI], 1.73 to 19.51) (Fig. 1b and Supplementary Table 1), suggesting a strong association of the *GANC* gene with human susceptibility to H7N9 virus infection. Additionally, we identified five low-frequency and heterozygous functional variants in the *GANC* gene (Fig. 1c and Supplementary Table 2), confirmed by Sanger sequencing (Supplementary Fig. 1a). These variants were conserved across different species including macaque, pig, dog, ferret, mouse, mallard, and chicken (Supplementary Fig. 1b), and showed no discernible hotspot mutation features (Supplementary Fig. 1c). Among these mutations, we observed that the 769I to 769N mutation slightly reduced the expression level of the GANC protein, while the 652R to 652Q mutation had no obvious effect, the remaining mutations led to truncated proteins (Fig. 1d). Subsequently, we investigated the impact of H7N9 virus infection on GANC expression in vitro and in vivo. A549 cells were infected with the H7N9 virus at a multiplicity of infection (MOI) of 0.1 (Supplementary Fig. 2a). The results showed that the mRNA and protein levels of GANC were slightly lower in H7N9 virus-infected cells at 24 h, 36 h, and 48 h post-infection compared to control group (Supplementary Fig. 2b, c). Similarly, in vivo experiments demonstrated that inhibited mRNA and protein levels of GANC in H7N9 virus-infected mice compared to phosphate-buffered saline (PBS) treated-control mice on days 1 and 4 post-infection (Supplementary Fig. 2d–f). These findings indicated that H7N9 virus infection decreased GANC expression.

### GANC facilitates H7N9 virus replication in vitro

**Fig. 2:**
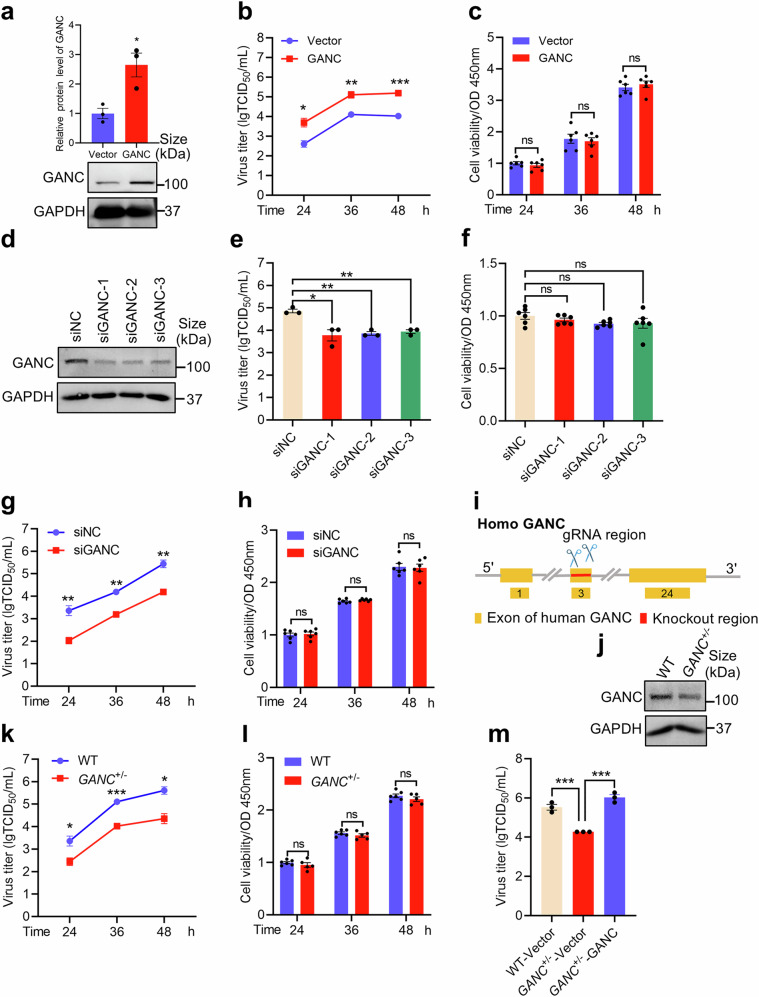
GANC promotes H7N9 virus replication in vitro. **a** Western blotting for confirmation of GANC overexpression stable strain. **b** Virus titers in supernatants from GANC overexpression stable strain and A549 cells infected with H7N9 virus at a MOI of 0.1, were determined at 24 h, 36 h, and 48 h post-infection using TCID_50_ assays in MDCK cells. **c** Assessment of cell viability in GANC overexpression stable strain. **d** Western blotting analysis of A549 cells transfected with GANC siRNA for 48 h. **e** Virus titers in supernatants from GANC siRNA-transfected A549 cells infected with H7N9 virus at a MOI of 0.1, were determined at 36 h post-infection using TCID_50_ assays in MDCK cells. **f** Assessment of cell viability following GANC siRNA transfection. **g** Virus titers in supernatants from GANC siRNA-transfected A549 cells infected with H7N9 virus at an MOI of 0.1, were determined at 24 h, 36 h, and 48 h post-infection using TCID_50_ assays in MDCK cells. **h** Assessment of cell viability following GANC siRNA transfection at 24 h, 36 h, and 48 h. **i**, **j** Schematic representation of sgRNA used for *GANC* gene in A549 cells (**i**), and identification of endogenous GANC proteins in *GANC*^*+/*^^*−*^ cells by Western blotting (**j**). **k** Virus titers in supernatants from WT A549 and *GANC*^*+/*^^*−*^ cells infected with H7N9 virus at a MOI of 0.1, were determined at 24 h, 36 h, and 48 h post-infection using TCID_50_ assays in MDCK cells. **l** Assessment of cell viability in *GANC*^*+/*^^*−*^ cells. **m** Virus titers in supernatants from WT A549 and *GANC*^*+/*^^*−*^ cells, transfected with GANC or vector plasmids, then infected with H7N9 virus at a MOI of 0.1, were determined at 48 h post-infection using TCID_50_ assays in MDCK cells. Quantification was shown as mean ± SEM. *n* = 3 independent experiments. The Student’s *t*-test (unpaired, two-tailed) was used to compare two independent groups. *P < 0.05, **P < 0.01, ***P < 0.001, ns not significant, NC negative control, WT wild-type, MOI multiplicity of infection, TCID_50_ tissue culture infective dose

To evaluate the impact of GANC on H7N9 virus replication in vitro, we established a GANC overexpression stable A549 cell line, the expression level of GANC was increased by at least two times after GANC overexpression in A549 cells, confirmed by Western blotting (Fig. 2a). A significant virus titer promotion was observed in the GANC overexpression stable strain-infected with H7N9 virus at a MOI of 0.1 compared to vector groups (Fig. 2b). Moreover, no significant effect of GANC overexpression on cell viability was observed (Fig. 2c). We further validated GANC’s role using three different small interfering RNAs (siRNAs), which effectively reduced the GANC protein expression levels by at least 50% in A549 cells (Fig. 2d). Following H7N9 virus infection post-siRNA transfection, we observed a significant reduction in virus titer (Fig. 2e), while cell viability remained unaffected (Fig. 2f). Meanwhile, we also found a significant reduction in virus titer at 24 h, 36 h, and 48 h post-infection (Fig. 2g), with no significant impact on cell viability (Fig. 2h). We then generated a heterozygous *GANC* knockout A549 cell line (referred to as *GANC*^*+/−*^) (Fig. 2i), confirmed by Western blotting (Fig. 2j). Upon infecting these cells with H7N9 virus, we noted a significant decrease in virus titer compared to wild-type (WT) A549 cells (Fig. 2k), with no significant impact on cell viability (Fig. 2l). Furthermore, virus titer was elevated following the increased expression of GANC in *GANC*^*+/*^^*−*^ cells (Fig. 2m). Taken together, these results indicated that GANC might promote H7N9 virus replication in vitro. In addition, we observed a significant reduction in virus titer following the H9N2 virus (Supplementary Fig. 3a, b) or H1N1-PR8 virus (Supplementary Fig. 3c, d) infection, post-siRNA transfection, or in *GANC*^*+*/^^*−*^ cells. These results suggested that GANC might regulate the replication of multiple influenza viruses.

### GANC facilitates H7N9 virus replication in vivo

**Fig. 3:**
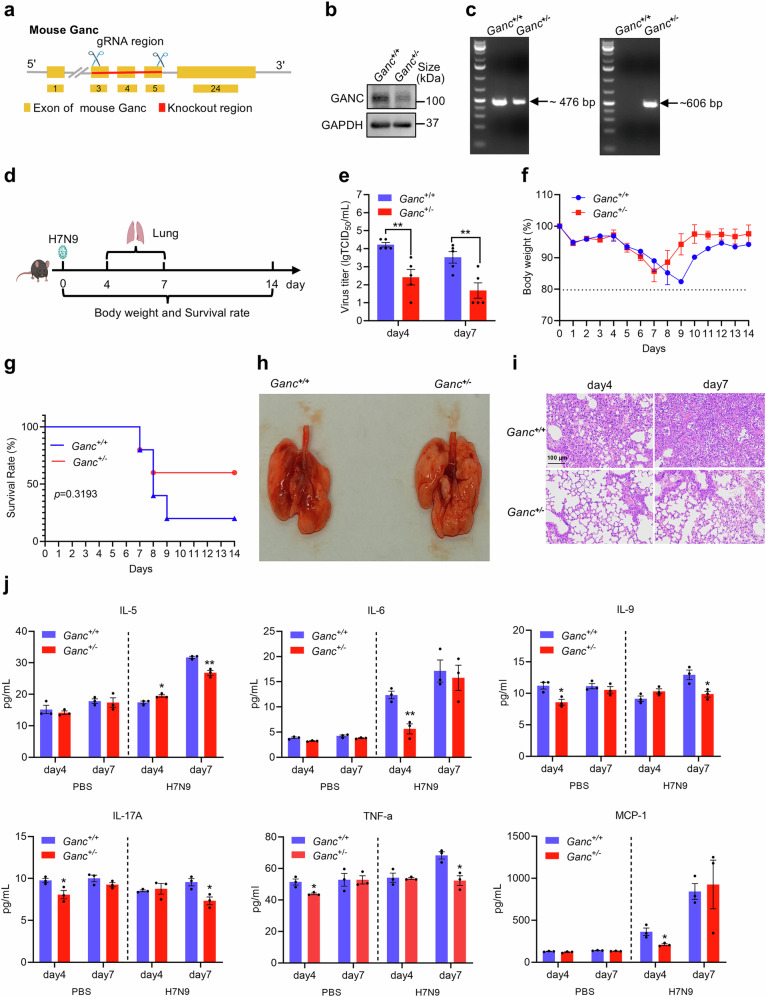
GANC promotes H7N9 virus replication in vivo. **a**, **b** Schematic representation of sgRNA used for *Ganc* gene in mice (**a**), and identification of endogenous GANC protein by Western blotting (**b**). **c** PCR analysis of genomic DNA confirmed the genotypes of *Ganc*^*+/+*^ and *Ganc*^*+/−*^ mice. The gene fragments of the *Ganc* chromosome in mice, with sizes of 476 bp and 606 bp, respectively. **d** Overview of mouse experiments. Six-week-old female C57BL/6 *Ganc*^*+/−*^ and *Ganc*^*+/+*^ mice were intranasally inoculated with 3 MLD_50_ per mouse with H7N9 virus. Lung tissues were collected from five mice per group at days 4 and 7 post-infection for viral titer and histopathology analysis. Five mice per group were monitored for survival for 14 days post-infection. **e** Virus titers in the lungs of *Ganc*^*+/−*^ and *Ganc*^*+/+*^ mice were determined by TCID_50_ assays in MDCK cells. **f** Daily monitoring of body weights in infected mice over 14 days. **g** Survival rate analysis of infected mice, including those humanely sacrificed after losing more than 20% of body weight post-infection. **h** Lung images of *Ganc*^*+/−*^and *Ganc*^*+/+*^ mice at day 7 post-infection. **i** Histopathological analysis of lung lesions. Scale bars, 100 μm. **j** Cytokine levels in lung of *Ganc*^*+/+*^ or *Ganc*^*+/−*^ mice were intranasally inoculated with PBS or 3 MLD_50_ per mouse with H7N9 virus. Quantification was shown as mean ± SEM. *n* = 3 or 5 independent experiments. The Student’s *t*-test (unpaired, two-tailed) was used to compare two independent groups. *P < 0.05, ^**^P < 0.01. MLD_50_ mouse lethal dose

To further investigate the role of GANC during H7N9 infection in vivo, we generated heterozygous *Ganc* knockout mice (referred to as *Ganc*^*+/*^^*−*^) (Fig. 3a). The successful depletion of GANC in the lungs was confirmed by Western blotting, which showed a clear comparison to WT mice (referred to as *Ganc*^*+/+*^) (Fig. 3b). The genotypes were further verified by PCR analysis of genomic DNA for *Ganc* (Fig. 3c). Subsequently, the *Ganc*^*+/−*^ and *Ganc*^*+/+*^ mice were intranasally inoculated with H7N9 virus (3 mouse lethal dose [MLD_50_]) (Fig. 3d). Consistent with in vitro findings, the *Ganc*^*+/−*^ mice exhibited lower virus titers at days 4 and 7 post-infection in the lungs (Fig. 3e). Meanwhile, no significant differences in the body weight in both the *Ganc*^*+/−*^ mice and the *Ganc*^*+/+*^ mice (Fig. 3f). Moreover, while 40% of *Ganc*^*+/−*^ mice died from infection at day 9, only 20% of the *Ganc*^*+/+*^ mice survived post-infection (Fig. 3g). Pulmonary imaging at day 7 and pathology analysis revealed that the *Ganc*^*+/−*^ mice had less severe lung injury compared to control mice at days 4 and 7 post-infection (Fig. 3h, i), accompanied by decreased abundance of several inflammatory factors compared to the *Ganc*^*+/+*^ mice (Fig. 3j). To further assess the impact of GANC on the replication of H1N1-PR8, all mice were intranasally infected with 5 MLD_50_ of H1N1-PR8. We quantified virus loads in lung tissues on day 4 post-infection. The *Ganc*^*+/−*^ mice exhibited slightly lower virus loads compared to the *Ganc*^*+/+*^ mice (Supplementary Fig. 4a). Meanwhile, the body weight and survival rate of the mice were monitored for 5 days, and all the mice had died by day 5. During this period, we observed that no significant differences in the body weight in both the *Ganc*^*+/−*^ mice and the *Ganc*^*+/+*^ mice (Supplementary Fig. 4b). Moreover, while 50% of the *Ganc*^*+/−*^ mice succumbed to infection at day 4, 100% of the *Ganc*^*+/+*^ mice died after infection, and 100% of the *Ganc*^*+/−*^ mice succumbed to infection at day 5 (Supplementary Fig. 4c). In the *Ganc*^*+/+*^ mice groups, histopathological observations suggested extensive lung tissue damage, such as alveolar wall thickening, connective tissue hyperplasia, massive alveolar detachment with hemorrhage and inflammatory cell infiltration on day 4 post-infection. However, lower lung tissue damage was observed in the lungs of mice in the *Ganc*^*+/−*^ mice, which showed a lighter cellular infiltration and inflammation (Supplementary Fig. 4d). These results underscored the crucial role of GANC in promoting H7N9 and H1N1-PR8 virus replication in mice.

### GANC knockdown promotes the proteasome-dependent degradation of HA

**Fig. 4:**
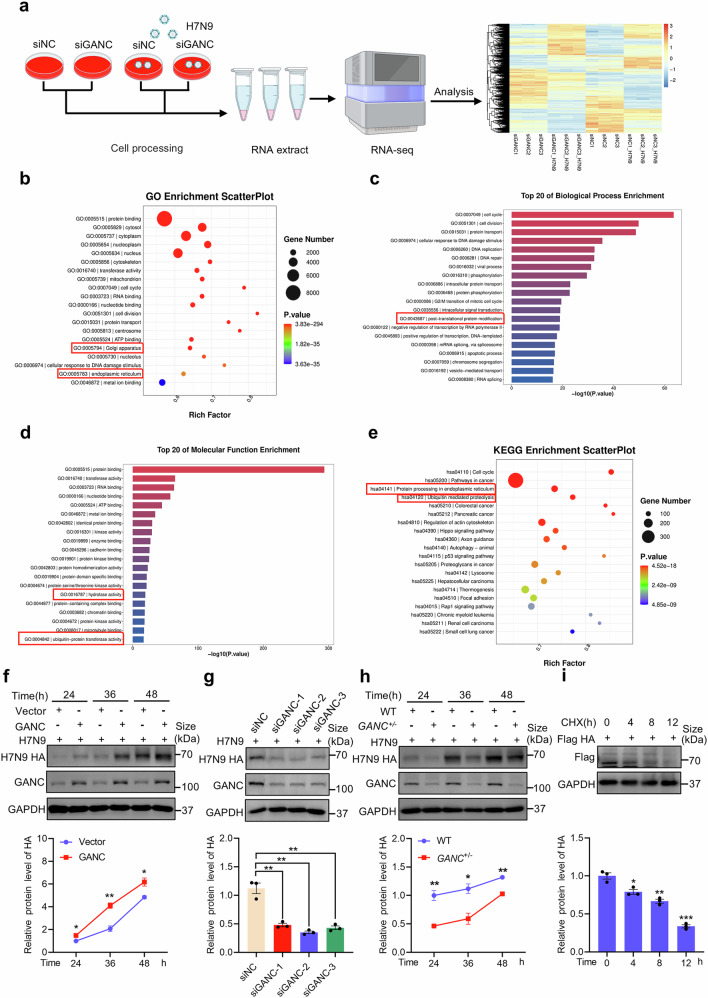
GANC may affect HA degradation. **a** Workflow of RNA-seq data analysis following GANC knockdown, with a heatmap depicting DEGs in A549 cells, with or without H7N9 virus infection at an MOI of 0.1. **b** GO enrichment analysis of DEGs after GANC knockdown in both non-infected and H7N9-infected A549 cells. **c**, **d** Top 20 enriched biological processes (**c**) and molecular functions (**d**) among the DEGs after GANC knockdown in both non-infected and H7N9-infected A549 cells. **e** KEGG pathway analysis of the DEGs after GANC knockdown in both non-infected and H7N9-infected A549 cells. **f**–**h** Western blotting analysis of HA protein levels in GANC overexpression stable strain (**f**), A549 cells transfected with GANC siRNA (**g**), or *GANC*^*+/−*^ cells (**h**) infected with H7N9 virus at a MOI of 0.1. Cells were sampled at the indicated time points post-infection. **i** A549 cells were transfected with HA plasmids tagged with Flag, treated with CHX (40 μg/mL), and cells were collected at the indicated times for Western blotting. Quantification was shown as mean ± SEM. *n* = 3 independent experiments. The Student’s *t*-test (unpaired, two-tailed) was used to compare two independent groups. *P < 0.05, **P < 0.01, ***P < 0.001. DEG differentially expressed gene, MOI multiplicity of infection, CHX cycloheximide

To elucidate the mechanism by which GANC affects H7N9 virus replication, we conducted RNA sequencing (RNA-Seq) analysis using A549 cells transfected with GANC siRNAs, and with or without H7N9 virus infection, revealing differentially expressed genes (DEGs) (Fig. 4a). Based on Gene Ontology (GO) enrichment analysis, identified DEGs in the ER and Golgi apparatus, associated with protein processing (Fig. 4b). The biological process enrichment analysis indicated that DEGs involved post-translational protein modification (Fig. 4c), while molecular function enrichment of DEGs included hydrolase activity and ubiquitin-protein transferase activity (Fig. 4d). Additionally, Kyoto Encyclopedia of Genes and Genomes (KEGG) pathway analysis revealed pathways related to protein processing in the ER and ubiquitin-mediated proteolysis (Fig. 4e and Supplementary Fig. 5a, b). Sequence comparison suggests that GANC is most similar to GIIα of GANAB,^15^ so GANC may have the functions of ER α-Glucosidases II. Considering HA protein is the predominant glycoprotein on the surface of the influenza virus,^36,37^ we hypothesize that HA may be a target for GANC. Indeed, GANC overexpression increased HA protein levels at 24 h, 36 h, and 48 h post-infection (Fig. 4f), while GANC siRNA or *GANC*^*+/−*^ cells reduced HA protein levels in A549 cells infected with H7N9 virus (Fig. 4g, h). Subsequently, we treated A549 cells overexpressing H7N9 HA plasmids with cycloheximide (CHX) to inhibit protein synthesis and determine protein half-life. We observed intracellular degradation of HA protein over time, suggesting that HA might be degraded through intracellular degradation pathways (Fig. 4i).

**Fig. 5:**
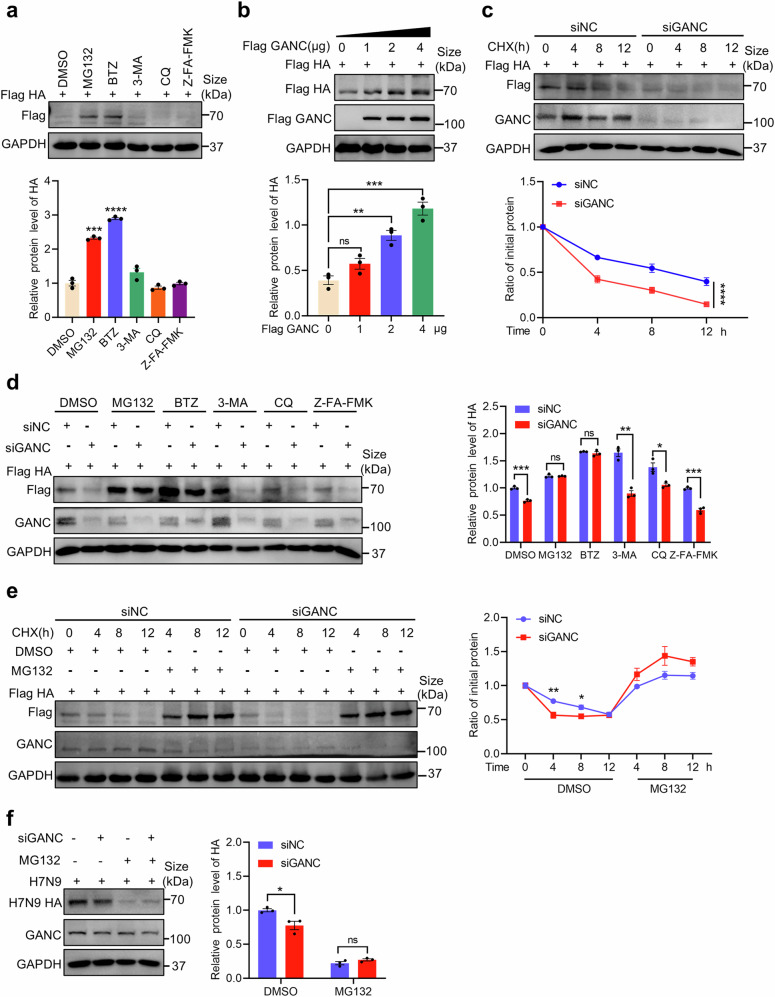
GANC knockdown increases the proteasome-dependent degradation of HA in A549 cells. **a** A549 cells were transfected with HA plasmids tagged with Flag, treated with DMSO, MG132 (20 μM), BTZ (20 μM), 3-MA (1 mg/mL), CQ (100 μM), or Z-FA-FMK (50 μM) for 12 h before collection. Protein levels of HA were then assessed by Western blotting. **b** A549 cells were transfected with HA plasmids tagged with Flag, and cotransfected with increasing amounts of plasmids containing GANC tagged with Flag. Cells were collected 48 h post-transfection for analysis of HA protein levels by Western blotting. **c** A549 cells were transfected with GANC siRNA, along with HA plasmids tagged with Flag, treated with CHX (40 μg/mL), and collected at indicated times to assess HA protein levels by Western blotting. **d** A549 cells were transfected with GANC siRNA, along with HA plasmids tagged with Flag, and treated with different chemical inhibitors for 12 h before collection. HA protein levels were evaluated by Western blotting. **e** A549 cells were transfected with GANC siRNA, along with HA plasmids tagged with Flag, and cotreated with CHX (40 μg/mL) and either DMSO or MG132 (20 μM). Cells were collected at the indicated times for Western blotting to analyze HA protein levels. **f** A549 cells were transfected with GANC siRNA and infected with H7N9 virus, then treated with DMSO or MG132 (20 μM). Cells were collected at 24 h for Western blotting to analyze HA protein levels. Quantification was shown as mean ± SEM. *n* = 3 independent experiments. The Student’s *t*-test (unpaired, two-tailed) was used to compare two independent groups, and a two-way ANOVA test was performed for comparisons of multiple groups. *P < 0.05, **P < 0.01, ***P < 0.001, ^****^P < 0.0001, ns not significant, CHX cycloheximide

Chemical inhibitors testing revealed that HA degradation was significantly reduced by proteasome inhibitors MG132 and bortezomib (BTZ) (Fig. 5a and Supplementary Fig. 6a), supporting the involvement of the proteasome system in HA degradation processing. However, inhibitors of autophagosome (3-Methyladenine [3-MA]), lysosomal (chloroquine [CQ]), and apoptosis (Z-FA-FMK) showed no effect on HA degradation in A549 and HEK293T cells (Fig. 5a and Supplementary Fig. 6a). Next, we explored the regulatory effect of GANC on HA expression. Initially, we overexpressed an increasing amount of GANC, and observed a corresponding increase in HA protein levels (Fig. 5b and Supplementary Fig. 6b). Subsequently, we assessed the effect of GANC knockdown on the stability of HA protein. Decreased GANC expression resulted in a significantly shorter half-life of HA compared to controls (Fig. 5c and Supplementary Fig. 6c). A549 and HEK293T cells were then co-transfected with HA plasmids and GANC siRNA in the presence or absence of chemical inhibitors. The results showed that GANC knockdown enhanced HA degradation, which was rescued by treatment with MG132 and BTZ. Conversely, 3-MA, CQ, or Z-FA-FMK showed no obvious effect (Fig. 5d and Supplementary Fig. 6d). Furthermore, the half-life of HA protein gradually increased in a time-dependent manner in the presence of MG132 following GANC siRNA transfection (Fig. 5e and Supplementary Fig. 6e). Similarly, we observed that MG132 treatment and GANC knockdown no longer regulated HA protein degradation in H7N9-infected A549 cells (Fig. 5f). These findings suggested that decreased GANC expression might promote the degradation of HA in a proteasome-dependent manner. Next, to test whether GANC specifically regulates the HA protein, we investigated the effect of GANC on the H7N9 neuraminidase (NA) protein. We overexpressed increasing amounts of GANC in HEK293T cells. The results showed that as GANC expression increased, the protein levels of NA also increased (Supplementary Fig. 7a), while GANC knockdown resulted in a decrease in the protein level of NA (Supplementary Fig. 7b). These results indicated that the HA protein was not the sole target through which GANC regulates the replication of the H7N9 virus. Moreover, to test whether GANC specifically regulates enveloped viruses, we further assessed the impact of GANC on the enveloped virus Zika virus (ZIKV) and the nonenveloped virus Adenovirus 5 (Ad-5) in A549 cells. We found that knockdown of GANC had no effect on the expression of ZIKV envelope protein (E) (Supplementary Fig. 8a). However, knockdown of GANC promoted the replication of Ad-5 (Supplementary Fig. 8b), while Ad-5 replication was inhibited when GANC was overexpressed (Supplementary Fig. 8c). The above results collectively suggested that the regulation of viral replication by GANC was not specific to IAV and may play distinct roles in both enveloped and non-enveloped viruses.

### GANC facilitates the ubiquitination of HA

In the proteasome-mediated degradation system, substrates should be ubiquitinated before proceeding.^38^ Through Co-immunoprecipitation (Co-IP), we observed ubiquitination of HA in the presence of both endogenous and overexpressed ubiquitin (Supplementary Fig. 9a, b). Overexpression of K48- or K63-linked ubiquitin chains along with HA revealed the formation of polyubiquitin chains on HA through both K48 and K63 linkages (Supplementary Fig. 9c, d). Subsequently, we screened for E3 ligases capable of ubiquitinating HA in HA-transfected HEK293T cells using immunoprecipitation followed by mass spectrometry (IP-MS), identified three potential candidates, STUB1, HECT domain E3 ubiquitin protein ligase 1 (HECTD1), and TRIM21 (Supplementary Table 3). Further investigation confirmed the interaction of HA with STUB1, but not with HECTD1 and TRIM21, using either STUB1 or HA as the bait protein (Supplementary Fig. 10a–c). Confocal microscopy confirmed the co-localization of STUB1 and HA (Supplementary Fig. 10d). Subsequent in vitro pulldown assays validated the interaction after purified STUB1 and HA (Supplementary Fig. 10e, f). We then performed STUB1 siRNA knockdown (Supplementary Fig. 11a) or utilized STUB1-deficient HEK293T cells (Supplementary Fig. 11d) to investigate the regulatory role of STUB1 in HA degradation. Stability assays with CHX treatment confirmed an extended half-life of HA (Supplementary Fig 11b, e), alongside a reduction in polyubiquitin chains for HA both under transfected siRNA conditions and in the *STUB1* knockout cells (Supplementary Fig. 11c, f). Furthermore, STUB1 overexpression significantly reduced HA protein levels in a dose-dependent manner, regardless of H7N9 virus infection (Supplementary Fig. 11g, h). Additionally, the stability assay with CHX treatment further confirmed a decreased half-life of HA (Supplementary Fig. 11i), and increased formation of polyubiquitin chains (Supplementary Fig. 11j). After overexpression of STUB1, MG132 treatment no longer decreased the protein level of HA (Supplementary Fig. 11k). Furthermore, we performed an in vitro ubiquitination assay (Supplementary Fig. 11l). To determine whether STUB1-mediated HA ubiquitination occurred via K48 or K63, we performed ubiquitination assays using Myc-tagged K48 and K63 revealing that overexpression of STUB1 obviously increased K48- linked ubiquitination (Supplementary Fig. 11m), but K63- linked ubiquitination (Supplementary Fig. 11n). However, K48R- linked ubiquitination substantially eliminated the STUB1-mediated polyubiquitination of HA (Supplementary Fig. 11o). These findings collectively suggested that STUB1 mediated the degradation of HA through the ubiquitin-proteasome system.

Next, we investigated whether GANC mediated the ubiquitination-dependent degradation of HA. Unexpectedly, GANC knockdown resulted in a decreased level of endogenous ubiquitination of HA (Supplementary Fig. 12a). In contrast, GANC overexpression increased the endogenous ubiquitination level of HA (Supplementary Fig. 12b). These findings indicated that knockdown of GANC promoted the degradation of the HA protein, which may be partly dependent on ubiquitination modification.

### GANC knockdown facilitates HA degradation via PSMD1 and PSMD2

**Fig. 6:**
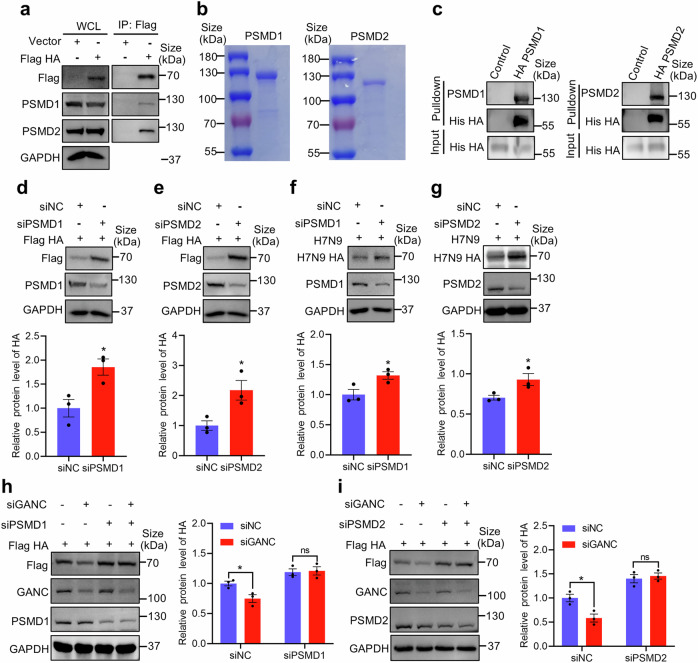
GANC knockdown increases the PSMD1/2-dependent degradation of HA. **a** HEK293T cells were transfected with HA plasmids tagged with Flag. 48 h later, the WCLs were incubated with anti-Flag magnetic beads, and subjected to Western blotting to detect PSMD1 or PSMD2. **b** Purification and Coomassie staining analysis of PSMD1 and PSMD2 proteins. **c** Detection of pulldown proteins by Western blotting after incubating anti-HA magnetic beads with HA proteins tagged with His. **d**, **e** Western blotting analysis of HA protein levels in HEK293T cells transfected with siRNA targeting PSMD1 (**d**) or PSMD2 (**e**), along with HA plasmids tagged with Flag, collected 48 h post-transfection. **f**, **g** Western blotting analysis of HA protein levels in A549 cells transfected with siRNA targeting PSMD1 (**f**) or PSMD2 (**g**), and infected with H7N9 virus at a MOI of 0.1. Cells were sampled at 48 h post-infection. **h**, **i** HEK293T cells were transfected with GANC, PSMD1 (**h**) or PSMD2 (**i**) siRNA, along with HA plasmids tagged with Flag, and cells were collected 48 h post-transfection for analysis of HA protein levels by Western blotting. Quantification was shown as mean ± SEM. *n* = 3 independent experiments. The Student’s *t*-test (unpaired, two-tailed) was used to compare two independent groups. *P < 0.05, ns not significant, WCL whole-cell lysate, MOI multiplicity of infection

To further investigate the host proteins that interact with HA. Based on the number of peptides identified for each protein showed two high-confidence candidates, PSMD1 and PSMD2 (Supplementary Table 4). In further experimental validation, Co-IP assays confirmed the interaction between HA and endogenous and exogenous PSMD1/2 in the non-infected group and upon H7N9 infection (Fig. 6a and Supplementary Fig. 13a–c). Confocal microscopy confirmed the co-localization of PSMD1/2 and HA (Supplementary Fig. 13d). Pulldown assays further validated the interaction following the purification of PSMD1, PSMD2, and HA (Fig. 6b, c). Meanwhile, knockdown of PSMD1 or PSMD2 increased HA protein expression level in HEK293T cells (Fig. 6d, e), and H7N9-infected A549 cells (Fig. 6f, g), suggesting their involvement in HA degradation. Next, we investigate whether PSMD1 and PSMD2 are involved in GANC-mediated regulation of HA protein degradation. Firstly, we reduced the expression levels of PSMD1, PSMD2, and GANC in HEK293T cells using siRNA, followed by overexpression of the HA plasmid. Knockdown of GANC alone promoted the degradation of the HA protein, however, HA protein degradation no longer occurred when the expression levels of both GANC and PSMD1 (Fig. 6h) or PSMD2 (Fig. 6i) were reduced simultaneously. These results collectively suggested that GANC knockdown promoted the degradation of the HA protein, which is dependent on PSMD1 and PSMD2.

### GANC inhibits the degradation of HA by decreasing its binding affinity to PSMD1 and PSMD2

**Fig. 7:**
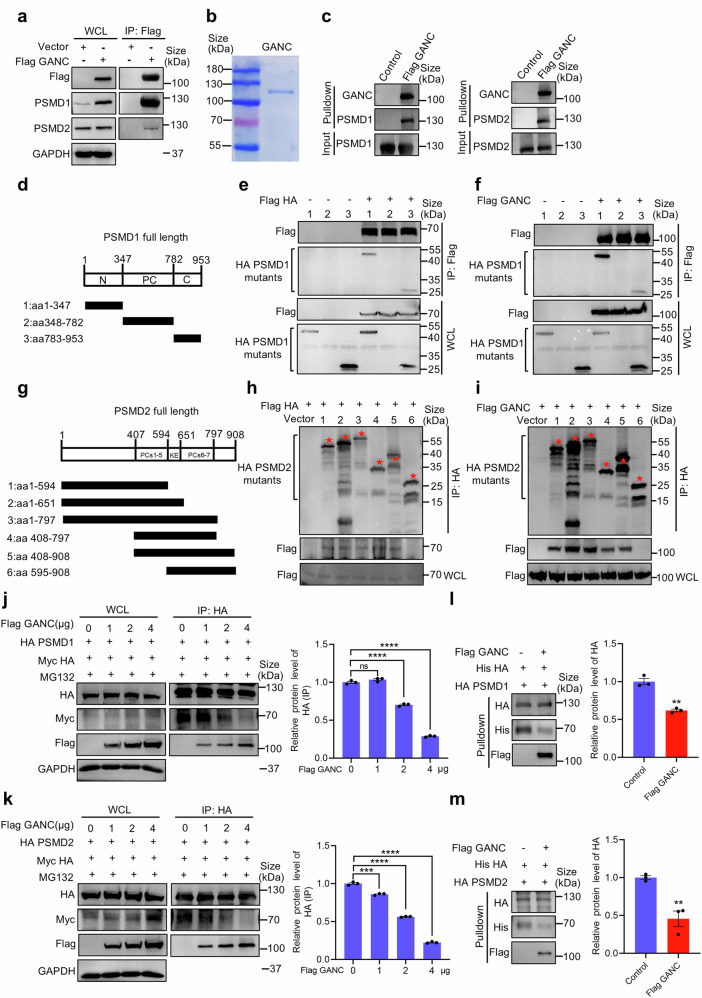
Competitive binding of GANC and HA to PSMD1 or PSMD2. **a** HEK293T cells were transfected with GANC plasmids tagged with Flag. 48 h later, the WCLs were incubated with anti-Flag magnetic beads and subjected to Western blotting to detect PSMD1 or PSMD2. **b** Purification and Coomassie staining analysis of GANC proteins. **c** Detection of pulldown proteins by Western blotting after incubating anti-Flag magnetic beads with GANC proteins, followed by incubation with PSMD1 or PSMD2 tagged with HA. **d** Schematic diagram of various truncations of PSMD1. **e**, **f** Western blotting for the detection of PSMD1 mutants in HEK293T cells transfected with PSMD1 truncations tagged with HA, along with HA plasmids tagged with Flag (**e**), or GANC plasmids tagged with Flag (**f**). WCLs were incubated with anti-Flag magnetic beads. **g** Schematic diagram of various truncations of PSMD2. **h**, **i** Western blotting for the detection of HA (**h**) or GANC (**i**) in HEK293T cells transfected with PSMD2 truncations tagged with HA, along with HA plasmids tagged with Flag, or GANC plasmids tagged with Flag. The red pentagons indicate the locations of various truncations of PSMD2. WCLs were incubated with anti-HA magnetic beads. **j**, **k** HEK293T cells transfected with HA plasmids tagged with Myc, followed by transfection with increasing amounts of GANC plasmids tagged with Flag, along with PSMD1 (**j**) or PSMD2 (**k**) plasmids tagged with HA. Cells were treated with MG132 (20 μM) for 12 h before collection. Western blotting was performed after incubating the WCLs with anti-HA magnetic beads. **l**, **m** Detection of pulldown proteins by Western blotting after incubating anti-HA magnetic beads with PSMD1 (**l**) or PSMD2 (**m**) proteins tagged with HA for 12 h, followed by incubation with HA protein tagged with His, and GANC protein tagged with Flag. Quantification was shown as mean ± SEM. *n* = 3 independent experiments. The Student’s *t*-test (unpaired, two-tailed) was used to compare two independent groups. **P < 0.01, ***P < 0.001, ****P < 0.0001. WCL whole-cell lysate

To elucidate how GANC knockdown degraded the HA protein via PSMD1 and PSMD2, we conducted IP-MS to screen for proteins that interacted with GANC. We pulled down the GANC protein from HEK293T cells. Through an overlap analysis, we identified shared proteins between HA and GANC, specifically PSMD1 and PSMD2 (Supplementary Table 5). Co-IP assays confirmed the interaction between GANC and endogenous and exogenous PSMD1/PSMD2 in the non-infected group and upon H7N9 infection. (Fig. 7a and Supplementary Fig. 14a–c). Confocal microscopy confirmed the co-localization of PSMD1/2 and GANC (Supplementary Fig. 14d). Pulldown assays further validated the interaction following the purification of GANC (Fig. 7b, c). To identify the functional domains mediating the interaction of HA and GANC with PSMD1 or PSMD2, we generated three truncated mutants of PSMD1 (Fig. 7d), but their PC region was undetectable, and six truncated mutants of PSMD2 (Fig. 7g), according to the methods provided by previous research.^39,40^ Co-IP assays showed that both the N-terminal and C-terminal regions of PSMD1 bound to HA or GANC (Fig. 7e, f), and the PCs1-5 of PSMD2 (aa 408-594) mutants bound to HA and GANC (Fig. 7h, i). Subsequently, competitive Co-IP assays indicated that GANC inhibited the interaction of HA with PSMD1/PSMD2, while enhancing its interaction with PSMD1/PSMD2, ultimately leading to increased HA protein levels in the whole-cell lysates (WCLs) (Fig. 7j, k). Pulldown assays further validated, GANC inhibited the interaction of HA with PSMD1/PSMD2 when GANC interacted with PSMD1/PSMD2 (Fig. 7l, m). However, there was no significant interaction between HA and GANC in the transfected and H7N9-infected cells (Supplementary Fig. 15a, b), suggesting that the degradation of the HA protein by GANC does not occur through interaction with it.

### *GANC* variants impair their ability to promote multiple influenza viruses replication

**Fig. 8:**
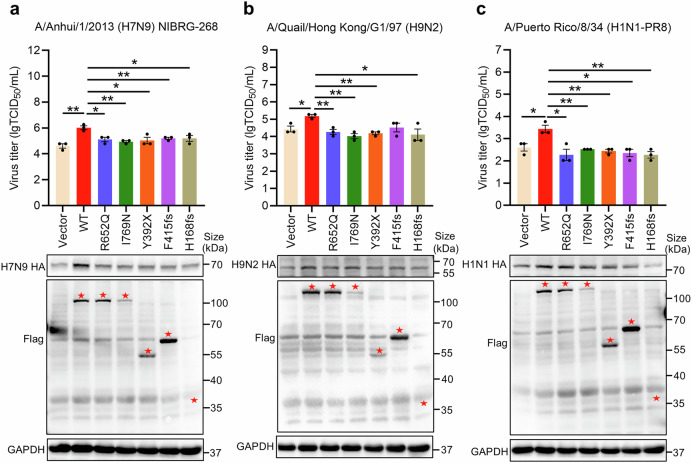
Mutations in the GANC gene impair its ability to promote multiple influenza viruses replication. **a**–**c** Virus titers in supernatants from cells overexpressing GANC or its mutations, infected with H7N9 (**a**), H9N2 (**b**) or H1N1-PR8 (**c**) virus at a MOI of 0.1, were determined at 36 h post-infection using TCID_50_ assays in MDCK cells. Western blotting analysis of HA protein levels in A549 cells transfected with plasmids encoding GANC or its mutations. The red pentagons indicate the locations of various mutations of GANC. Quantification was shown as mean ± SEM. *n* = 3 independent experiments. The Student’s *t*-test (unpaired, two-tailed) was used to compare two independent groups. *P < 0.05, **P < 0.01. MOI multiplicity of infection, TCID_50_ tissue culture infective dose

Through WGS and gene-based association analysis, we identified five potential variants in the *GANC* gene-H168fs, Y392X, F415fs, R652Q, and I769N that are associated with human H7N9 infection. We further explored whether these variants affect GANC’s regulation of H7N9 virus replication. The experimental results indicated that overexpression of GANC significantly increased H7N9 virus replication efficiency compared to the control group, however, all the five variants in the *GANC* gene reduced its ability to promote H7N9 virus replication, and also diminished the levels of GANC-induced HA protein expression (Fig. 8a). Additionally, we examined the effects of these variants on H9N2 and H1N1-PR8 virus replication. The results demonstrated that these variants similarly impaired their ability to enhance replication of both H9N2 and H1N1-PR8 viruses, and diminished their function in promoting HA protein expression (Fig. 8b, c). These findings indicated that these variants in the *GANC* gene found in H7N9 infections may impair its ability to facilitate the replication of influenza viruses.

## Discussion

Host factors regulate IAV infection by targeting viruses through various mechanisms. Transmembrane serine protease 2 (TMPRSS2) facilitates the HA cleavage, a critical step for viral entry,^31,41^ while interferon-induced transmembrane protein 1 (IFITM1) and IFITM2 inhibit the entry of IAV by blocking viral membrane fusion.^42^ MxA restricts IAV replication by targeting polymerase activity,^8^ and PSMD12 mediates M1 protein degradation via the ubiquitin-proteasome system.^33^ Inhibition of ER α-glucosidases I and II, has been shown to inhibit multiple virus families by altering glycoprotein processing, leading to their degradation both in vitro and in vivo.^18,43,44^ The GANAB protein, a glucosidase II, composed of GIIα and GIIβ, is crucial for QC and maturation of glycoproteins in the ER.^19,34^ The study indicates that GANC evolves from the GIIα of GANAB.^15^ The sequence comparison indicates that GANC is highly similar to GIIα. Specifically, the amino acid sequence similarity between human GIIα and human GANC is 69.1%, with an identity percentage of 49.4%.^15^ According to GO enrichment analysis, our results indicated that GANC may affect post-translational protein modification, leading to protein degradation. We discovered a mechanism by which GANC knockdown limits the infection of the H7N9 virus by targeting HA degradation. As HA protein is the predominant glycoprotein on the influenza virus surface,^31,32^ GANC overexpression increased the replication of H7N9, H9N2, and H1N1-PR8, thus, inhibiting GANC expression may have broad-spectrum anti-influenza virus activity. In addition to HA, NA is another key glycoprotein of the IAV.^45^ Our findings demonstrated that GANC enhanced the protein expression of H7N9 NA, indicating that HA is not the sole target through which GANC modulates H7N9 viral replication. Further investigations are needed to elucidate the mechanisms by which GANC regulates NA expression. Interestingly, GANC had no effect on the E protein of ZIKV, yet it inhibited the replication of Ad5. This suggests that GANC may exert distinct regulatory roles across different viruses, highlighting the complexity of its antiviral effects. These observations warrant further exploration to better understand the differential impacts of GANC on various viral pathogens.

IAV infection can induce changes in the expression of many host genes, some of which are involved in affecting the antiviral response, while others increase the replication of the virus.^46,47^ For example, upregulation of microRNA-203 during IAV infection inhibits viral replication,^48^ possibly due to the host cells upregulating the expression of antiviral factors to resist viral replication during the infection period. Conversely, when host genes promote viral replication, the host may suppress the expression of these genes. A study suggests that, upon initial exposure to the IAV, host cells downregulate the expression of miR-4276 to reduce viral replication.^49^ In our study, the expression levels of GANC were lower in H7N9 virus-infected cells and mice compared to the control group. We speculate that the host may limit the expression of GANC to inhibit viral replication. We will investigate it further if we can find a host gene in the future to explore the phenomenon.

Host genetic factors play a critical role in virus susceptibility, with various variants identified to increase susceptibility to IAV. Previous studies have highlighted the significant association between variants of the *MX1* gene, *GALECTIN-1* (rs13057866), and *TMPRSS2* (rs2070788 and rs383510) with susceptibility to H7N9 virus infection.^8,50,51^ In our research, we further identified a strong association between the *GANC* gene and H7N9 infections. These results underscored the significant role of host genetic factors in H7N9 virus susceptibility. Through WGS, we identified five low-frequency functional and heterozygous variants of *GANC* strongly associated with human H7N9 infections compared to healthy controls. The variant frequency of the *GANC* gene in H7N9-infected patients is higher than in the healthy population, thus, we hypothesize that the *GANC* variants should promote viral infection; however, these variants impair their ability to facilitate the replication of IAV. We speculate this discrepancy may arise from differences in the regulation of *GANC* variants at the cellular and in vivo levels. Currently, our research has only explored the impact of *GANC* variants on H7N9 virus replication at the cellular level, whereas the regulatory mechanisms in animals and humans are more complex. To further investigate the mechanisms by which *GANC* variants affect the virus, it will be necessary to establish animal models carrying *GANC* variants, allowing us to study the impact of these variants on H7N9 virus replication at the animal level.

MxA is an antiviral factor against the H7N9 virus, however, most of the MxA variants have lost the ability to inhibit H7N9 virus replication.^8^ We observed that GANC could promote H7N9 virus replication; however, the *GANC* variants lost or attenuated this ability to promote viral replication. All *GANC* variants attenuated the expression of HA protein rather than abolishing it, suggesting that *GANC* variants also affect its role in regulating other aspects of IAV replication, for example, the expression of other viral proteins, binding, entry, vRNP nuclear import, and coordination of viral particle assembly and budding.

Ubiquitination, a most common posttranslational modification, is important for protein degradation.^52^ STUB1, possessing E3 ubiquitin ligase activity,^53,54^ interacted with H7N9 HA, facilitating its ubiquitination, and subsequent proteasomal degradation. However, RNA-seq results indicated that the downregulation of GANC led to the suppression of STUB1 expression, which may play a significant role in the inhibition of HA protein expression following GANC knockdown.

In conclusion, our study demonstrated a significant association between the *GANC* gene and H7N9 infections. We elucidated, for the first time, that the H7N9 HA protein was degraded through either ubiquitination-independent or -dependent proteasome systems. GANC knockdown facilitated the degradation of H7N9 HA in a proteasome-dependent manner. This novel mechanism highlighted how GANC inhibited HA degradation, consequently promoting H7N9 virus replication through decreasing HA binding to PSMD1 and PSMD2.

## Materials and methods

### Mice

The heterozygous *Ganc* knockout C57BL/6N mice were created by CRISPR/Cas9 (Clustered Regularly Interspaced Short Palindromic Repeats/Cas9)-mediated gene editing from Biocytogen Co., Ltd. Mice were group-housed with a 12 h dark/light cycle, and had access to food and water randomly. All animal experiments were performed in compliance with the policies of Shenzhen Bay Laboratory, and were approved by the Animal Care and Use Committee of Sun Yat-sen University (assurance number, 2023-B035).

### Cells and viruses

Human non-small-cell lung cancer cells (A549), human embryonic kidney 293T cells (HEK293T), human cervical cancer cells (HeLa), and Madin-Darby canine kidney cells (MDCK) were cultured in Dulbecco’s modified Eagle’s medium (DMEM) (Gibco) supplemented with 10% fetal bovine serum (FBS) (Gibco) and 1% penicillin–streptomycin (P/S) (Gibco), and kept at 37 °C in a humidified atmosphere of 5% of CO_2_. The influenza virus A/Anhui/1/2013 (H7N9) NIBRG-268, A/Quail/Hong Kong/G1/97 (H9N2), and A/Puerto Rico/8/34 (H1N1-PR8) were kindly provided by the Chinese National Influenza Center (CNIC), National Institute for Viral Disease Control and Prevention, Chinese Center for Disease Control and Prevention. ZIKV was kindly provided by Huanle Luo from Sun Yat-sen University, and Ad-5 was kindly provided by Caijun Sun from Sun Yat-sen University.

### The heterozygous *GANC* knockout or *STUB1* knockout cell lines

The heterozygous *GANC* knockout A549 cell line was created by CRISPR/Cas9. Briefly, Guide RNA (gRNA) sequences were designed for CRISPR/Cas9 at the CRISPR design website (http://crispr.mit.edu/). Insert oligonucleotides for human *GANC* gRNA are 5′-ATTCTGAACAGGTATCTAGAGGG-3′ and 5′-GTGAGCCACTAATCACTAAAAGG-3′. For human *STUB1* gRNA is 5′-TCGCGATTCGAAGAGCGCTGGGG-3′. A549 or HEK293T cells were seeded in a 6-well plate and transfected with gRNA by using electroporation following the manufacturer’s protocols. Monoclonal was selected after electroporation. The GANC or STUB1 deficiency was confirmed by Western blotting, respectively.

### Construction of the GANC over-expression stable strain

To create a human GANC overexpression A549 cell line model using lentiviral technology. Briefly, human *GANC* CDS sequences were cloned into the LV-EFS > Kozak-Human *GANC* CDS-MV > T2A/Puro vector. A549 cells will be transduced by lentivirus, and the resistant cells were selected with puromycin (1 μg/mL) for at least 7 days. Human GANC expression will be detected by Western blotting in a GANC overexpression stably strain.

### IAV infection

The H7N9 virus was propagated in 9–11-day-old specific pathogen-free (SPF) embryonated chicken eggs and used to infect A549 cells. To determine the function of GANC, A549 cells were transfected siRNA targeting GANC, *GANC*^*+/−*^ or GANC overexpression stably A549 cells were cultured separately in 6-well plates and infected with H7N9, H9N2 or H1N1-PR8 virus for 1 h, washed three times with PBS, and then maintained at 37 °C in DMEM containing 1% bovine serum albumin (BSA) (Sigma-Aldrich), 1% P/S and tosylsulfo phenylalanyl chloromethyl ketone (TPCK)-trypsin (9002-07-7, Sigma-Aldrich) at 1 μg/mL. At the indicated times, the culture supernatant was harvested, and the cells were harvested using RIPA lysis buffer (P0013D, Beyotime Biotechnology). Mice were anesthetized with avertin (Sigma-Aldrich) and infected intranasally with H7N9 virus in 50 μL (3 MLD_50_), and H1N1-PR8 virus in 50 μL (5 MLD_50_). Mice were monitored daily and euthanized at different time points for sampling. The infected mice with more than 20% weight loss were euthanized and treated as dead.

### Median tissue culture infective dose (TCID_50_)

Initially, 1.5 × 10^5^ cells/mL MDCK cells per well were plated in a 96-well plate 24 h before viral infection. The following day, the virus was prepared at various dilutions in fresh, serum-free DMEM. The MDCK cells were then exposed to the diluted virus for 1 h, with gentle shaking every 20 mins. After infection, the virus solution was aspirated, and the cells were washed twice with PBS. The cytopathic effects, which indicated viral presence and correlated with the virus concentration, were recorded and used to calculate the viral titer using the Reed and Muench method.

### Cytokine Bio-Plex

The samples of cell culture supernatants and mouse lungs were collected for cytokine analysis using the Bio-Plex Pro Mouse Cytokine Grp I Panel 23-plex Assays (M60009RDPD, Bio-Rad), respectively. For each well, 20 μL of the sample and 35 μL of beads were added, followed by incubation at 37 °C for 1 h. After incubation, the beads were washed by adding 400 μL of wash buffer. Then, 20 μL of detection antibody was added, and the samples were incubated at 37 °C for 35 mins, followed by another wash of the beads. Subsequently, 50 μL of Bio-Plex SA-PE (Streptavidin-Phycoerythrin) was added, and the samples were incubated for 15 mins, followed by a final wash of the beads. Data were acquired using the Bio-Plex 200 System, and the Bio-Plex Software provided results as median fluorescence intensity (MFI) and concentration (pg/mL). The cytokine concentrations bound to each bead were directly correlated with the MFI signal. Data analysis will be conducted using Bio-Plex Data Software.

### RNA interference

The siRNAs sequences for target genes were as following: siGANC-1 (5’-GGUCAGAUCUCGCACUCAUTT-3’), siGANC-2 (5’-GGAUGUCUAUGGAUACCAATT-3’) and siGANC-3 (5’-GCUAAUGGCCCUUCUUCUATT-3’); siSTUB1-1 (5’-AGGCCAAGCACGACAAGUATT-3’), siSTUB1-2 (5’-GGCAAUCGUCUGUUCGUGGGCCGAA-3’), siSTUB1-3 (5’-GGCAGUCUGUGAAGGCGCACUUCUU-3’); siPSMD1 (5’-UGAUAAAAUACUUUAGAUGCC-3’); siPSMD2 (5’-GCGCCAGUUAGCUCAAUAUTT-3’), and negative control siRNA, siNC (5’-TTCTCCGAACGTGTCACGT-3’) were synthesized by GenePharma. The siRNA was used at a final concentration of 20 nM and transfected into cells using jetPRIME (101000046, Polyplus) according to the manufacturer’s instructions.

### RNA-Seq and data analysis

A549 cells were transfected with GANC siRNAs, and with or without H7N9 virus infection at an MOI of 0.1, and cells were harvested at 36 h post-infection for RNA-Seq and data analysis. All the experiments were conducted in triplicate. Transcriptome assembly and annotation protocols were provided by LC Bio-Technology Co., Ltd (Hangzhou, China). The RNA libraries were sequenced using the Illumina NovaseqTM 6000 platform. Transcriptome assembly and annotation protocols were provided by LC Bio-Technology Co., Ltd. Briefly, total RNA was extracted using TRIzol reagent (Invitrogen) following the manufacturer’s instructions. And mRNA was purified from total RNA using Dynabeads Oligo (dT). Then the cleaved RNA fragments were reverse-transcribed to create the cDNA libraries with average insert sizes of 300 ± 50 bp. The 2 × 150 bp paired-end sequencing (PE150) was performed on an Illumina Novaseq 6000. And bioinformatic analysis was performed using the OmicStudio tools at https://www.omicstudio.cn/tool.

### Plasmids, antibodies, and reagents

DNA fragments encoding the *GANC* were amplified from corresponding cDNAs in A549 cells and subcloned into a pcDNA3.1 or p3 × Flag-CMV-7.1 vector. H7N9 HA plasmid was kindly provided by Dr. Wenfei Zhu from CNIC, and then HA was amplified from the corresponding plasmid, and subcloned into the p3 × Flag-CMV-7.1 vector. *PSMD1*, *PSMD2*, and *STUB1* were generated by PCR from corresponding cDNAs in HEK293T cells, and subcloned into the pcDNA3.1 vector with an HA tag. Plasmids of WT ubiquitination, K48- and K63-linked ubiquitination were kindly provided by Zanxian Xia from Central South University. Anti-Flag M2 (F1084) was purchased from Sigma-Aldrich. Anti-H7N9 HA (40103-T62) and anti-H9N2 HA (11229-R106) were purchased from Sino Biological. Anti-GANC (sc-393697) and anti-PSMD1 (sc-166038) were from Santa Cruz Biotechnology. Anti-STUB1 (66974-1-Ig), anti-PSMD2 (66974-1-Ig), anti-ubiquitin (10201-2-A), anti-Myc tag (16286-1-AP), HRP-conjugated affinipure goat anti-mouse (SA00001-1), and goat anti-rabbit IgG (SA00001-2) were from Proteintech. Anti-HA tag (GTX115044), anti-ZIKV E (GTX133314), anti-H1N1 HA (GTX127357), and anti-GAPDH (GTX100118) were purchased from GeneTex. Goat anti-mouse IgG H&L (Alexa Fluor® 488) (ab150113) and goat anti-rabbit IgG H&L (Alexa Fluor® 594) (ab1150080) were from Abcam. CHX (HY12320), MG132 (HY-13259), BTZ (HY-10227), 3-MA (HY-19312), CQ (HY-17589A), and Z-FA-FMK (HY-P0109A) were purchased from MedChemExpress.

### Mass spectrometry

To identify H7N9 HA and GANC-associated interacting proteins, we performed IP-MS experiments by Shanghai Applied Protein Technology. HEK293T cells were transfected separately with p3 × Flag-CMV-7.1-H7N9 HA, p3 × Flag-CMV-7.1-GANC, or p3 × Flag-CMV-7.1 empty vectors for 48 h. HEK293T cells were treated with MG132 for 12 h before collection. Subsequently, HEK293T cells were harvested by RIPA lysis buffer containing protease inhibitor mixture (PMSF) (Beyotime Biotechnology) and *N*-ethylmaleimide (NEM) (S2692, Selleck). All cell lysates were then incubated with anti-Flag magnetic beads (M8823, Sigma-Aldrich) at 4 °C overnight. Magnetic bead-bound immune complexes were washed in lysis buffer for 6 × 5 mins and were eluted with sodium dodecyl sulfate (SDS) loading buffer. The proteins were digested by trypsin, and then all fractions were injected for nano LC-MS/MS analysis.

### Confocal microscopy

HeLa cells were seeded in confocal dishes and cells were transfected with plasmids encoding HA-tagged or Flag-tagged proteins when grown to 50–60% confluency. PBS-washed cells were fixed in 4% paraformaldehyde (PFA) (P0099, Beyotime Biotechnology) for 15 mins, followed by permeabilization with 0.1% Triton X-100 (P0096, Beyotime Biotechnology) in PBS for 10 mins. After a washing step with PBS, cells were incubated with 1% BSA for 1 h at room temperature, then were incubated with antibodies overnight at 4 °C. The next day, after 3 × 5 mins washes with PBS. The cells were incubated for 1 h at room temperature with the following secondary antibodies in 1% BSA buffer: goat anti-mouse IgG H&L (Alexa Fluor® 488) and goat anti-rabbit IgG H&L (Alexa Fluor® 594), respectively. Cells were washed with PBS, then cells were subsequently counterstained with Antifade Mounting Medium with 4′,6-diamidino-2-phenylindole (DAPI) (P0131, Beyotime Biotechnology) for 10 mins.

### Immunoprecipitation and western blotting

HEK293T cells were transfected with the corresponding plasmids. Cells were harvested with RIPA lysis buffer including 1% PMSF, then centrifuged at 15,000 rpm for 20 mins at 4 °C. The Co-IP assay was performed using anti-Flag magnetic beads or anti-HA magnetic beads (88837, Thermo Fisher Scientific), then WCLs were incubated with indicated magnetic beads at 4 °C overnight. Subsequently, the magnetic beads were washed in lysis buffer for 6 × 5 mins, then eluted with 1× SDS loading buffer and heated to 100 °C for 10 mins. Samples were evaluated using Western blotting with the corresponding antibodies. Then corresponding secondary antibodies were incubated.

### Expression and purification of STUB1, PSMD1, PSMD2, HA, and GANC

Codon-optimized genes encoding the *STUB1* were synthesized and cloned into the prokaryotic expression vector pET-28a. Codon-optimized genes encoding the *PSMD1* and *PSMD2* were synthesized and cloned into the prokaryotic expression vector pET-28a-sumo. Both genes included a His tag in the N-terminal, and a HA tag in the C-terminal. STUB1, PSMD1, and PSMD2 were expressed as full-length proteins in E. coli BL21-DE3 (Tsingke). Purification was performed using Ni column affinity chromatography purification. These proteins were eluted using elution buffer (20 Mm Tris-HCl, pH 8.0, 500 mM imidazole and 0.5 M NaCl); A DNA sequence encoding the influenza virus A/Anhui/1/2013(H7N9) HA (EPI439507) (Met1-Val524) was expressed with a C-terminal His tag. The gene encoding the HA ectodomain from A/Anhui/1/2013 (H7N9) was cloned into the baculovirus transfer vector pFastBac1 in-frame with an N-terminal gp67 signal peptide for secretion, a C-terminal thrombin cleavage site, a trimerization folded sequence, and a His at the extreme C terminus for purification. Purification was performed using Ni column affinity chromatography purification. For GANC protein, HEK293T cells were transfected with the Flag GANC-expressing plasmid. 48 h after transfection, cells were collected and lysed with RIPA lysis buffer supplemented with a protease inhibitor including PMSF. WCLs were sonicated, and incubated with anti-Flag magnetic beads at 4 °C overnight. The proteins were eluted from the beads with 0.2 M glycine-HCl buffer (pH 3.0), and neutralized with 1.0 M Tris-HCl buffer (pH 9.0).

### In vitro HA ubiquitination assay

In vitro ubiquitination was performed as per the manufacturer’s instructions from ‘Human CHIP Ubiquitin Ligase Kit—Glow-Fold Substrate’ (K-280, BostonBiochem). Briefly, the mixture of 1 μg Recombinant Human HSP70 Protein (P02346, Solarbio), 1 μg Recombinant Human HSP40 Protein (P02467, Solarbio), 4 mM ATP (A2383, Sigma) in reaction buffer (50 mM Tris [PH 7.4], 500 mM NaCl, 50 mM KCl) were heated for 7 mins at 43 °C, then immediately transfer to ice for 10 mins, then the anti-His magnetic beads with HA protein was incubated with STUB1, together with 200 ng of UBE1 (E-305-025, R&D Systems), 400 ng of UbcH5c/UBE2D3 (E2-627, R&D Systems), and 5 μg of recombinant ubiquitin (U-100H-10M, R&D Systems) at 30 °C for 1 h. The supernatant was removed, and the reaction was terminated by adding 2× loading buffer. The ubiquitin chain was detected by Western blotting.

### RNA isolation, reverse transcription, and RT-qPCR

Total intracellular RNA was isolated using Trizol reagent (15596026CN, Invitrogen). Total cDNA was synthesized by EasyScript® One-Step gDNA Removal and cDNA Synthesis SuperMix (AE311-03, TransGen Biotech) according to the manufacturer’s protocols. To quantify mRNA expression, quantitative real-time PCR (RT-qPCR) was performed with CFX96 Real-time PCR System and ChamQ Universal SYBR qPCR Master Mix (Q711-02, Vazyme). The RT-qPCR primers are listed in Supplementary Table 6. The selected gene mRNA levels relative to GAPDH have been calculated to obtain the relative mRNA expression.

### Statistical analysis

Statistical significance was assessed using two-way ANOVA for comparisons of multiple groups with GraphPad Prism 8.0. Two-tailed unpaired (Student’s) *t*-test was used if only two conditions were compared. Each experiment was repeated three times. Error bars show mean and standard deviation (mean ± SEM), as indicated in figure legends. ^*^P < 0.05, ^**^P < 0.01, ^***^P < 0.001, ^****^P < 0.0001, ns = not significant *P* > 0.05.

## Supplementary information

Supplementary_Materials

Uncropped Western Blots

## Data Availability

The MS spectrometry proteomics is available from the ProteomeXchange (http://proteomecentral.proteomexchange.org/cgi/GetDataset?ID=PXD061732) with the dataset identifier PXD061732, and the original data of the RNA-seq are stored in the GEO public database (https://www.ncbi.nlm.nih.gov/geo/) under the accession numbers GSE291573. The corresponding authors made the data utilized in the present investigation accessible to interested individuals upon a reasonable request.
