# Supplementary material for: Glucosidase alpha neutral C promotes influenza virus replication by inhibiting proteosome-dependent degradation of hemagglutinin
Source: Signal Transduct Target Ther. 2025 Apr 23;10:131. doi: 10.1038/s41392-025-02227-6 (PMC12015365; doi:10.1038/s41392-025-02227-6)
Supplement: Supplementary file 1 — Supplementary_Materials [file 41392_2025_2227_MOESM1_ESM.docx]

Supplementary Materials for

**Glucosidase alpha neutral C promotes influenza virus replication by inhibiting proteosome-dependent degradation of hemagglutinin**

Xinzhong Liao^1#^, Qian Xie^1#^, Minqi Liang^1^, Qijun Liao^1^, Bi Huang^1^, Shengze Zhang^1^, Feng Zhang^1^, Liangliang Wang^1^, Lifang Yuan^1^, Xuejie Liu^1^, Simin Wen^2^, Chuming Luo^1^, Dayan Wang^3^, Yongkun Chen^4*^, Huanle Luo^1,5*^, Yuelong Shu^1,6*^

Correspondence to: Yuelong Shu ([shuylong@mail.sysu.edu.cn](mailto:shuylong@mail.sysu.edu.cn)), Huanle Luo ([luohle@mail.sysu.edu.cn](mailto:luohle@mail.sysu.edu.cn)), and Yongkun Chen ([chenyk@szu.edu.cn](mailto:chenyk@szu.edu.cn)).

**This PDF file includes:**

Supplementary Fig. 1 to Fig. 15

Supplementary Table. 1 to Table. 6

Supplementary Fig. 1.


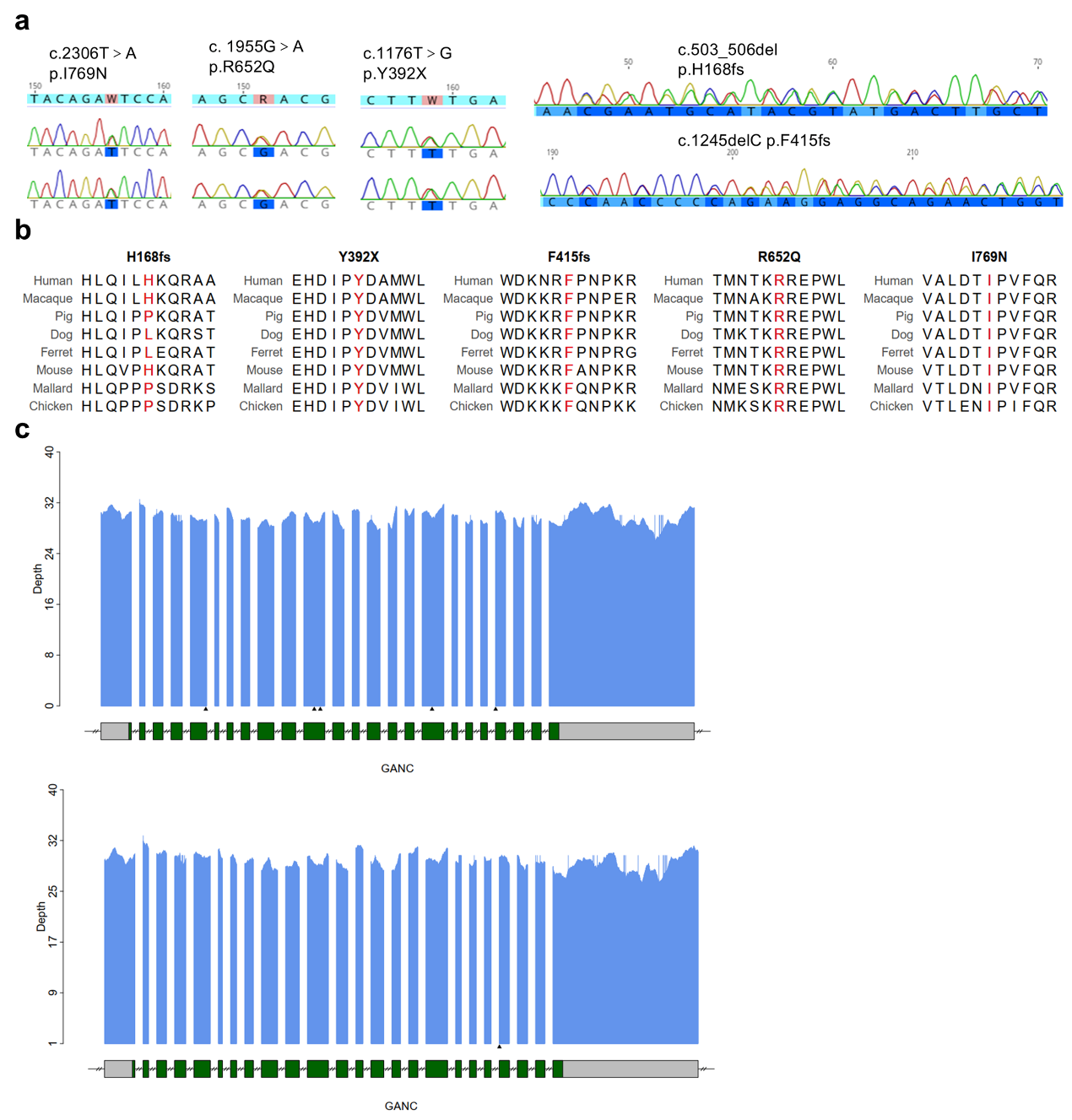


**Supplementary Fig. 1 Genetic mutations of the *GANC* gene in H7N9 infections (related to Fig. 1). a** The positions of five heterozygous SNVs in the *GANC* gene sequence fragments are highlighted in red in the consensus sequences shown above the electropherograms. Five SNVs were confirmed by Sanger sequencing in both directions. **b** Sequence conservation of the indicated mutation sites in the *GANC* gene among different species. **c** Schematic representation of the *GANC* gene with mutations in H7N9 infections (top) and healthy controls (bottom). The black triangles indicate the location of the mutation sites. SNV: single nucleotide variant.

Supplementary Fig. 2.


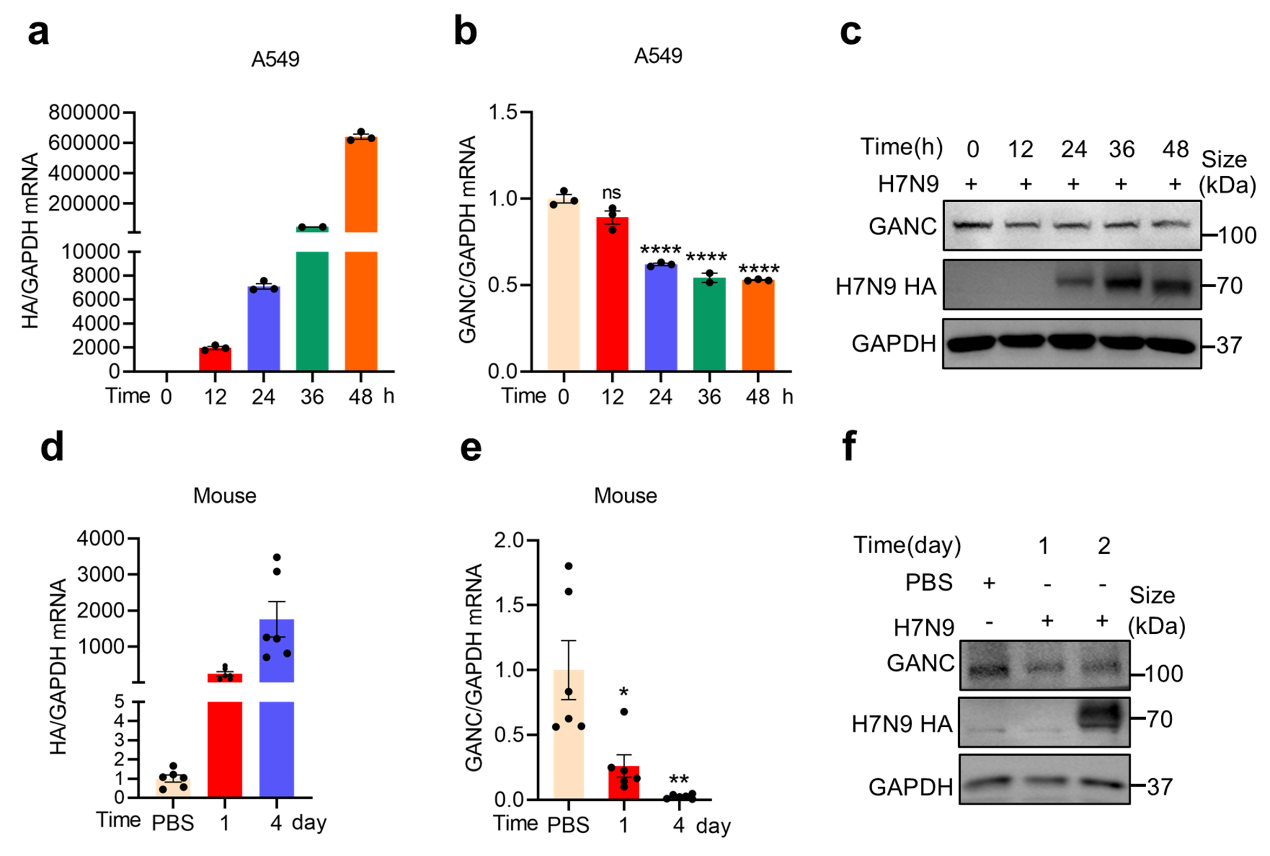


**Supplementary Fig. 2 H7N9 virus infection decreases the expression levels of GANC (related to Fig. 1). a-c** A549 cells were infected with H7N9 virus at a MOI of 0.1. Cells were collected at 12, 24, 36, and 48 h post-infection, and the mRNA (**a, b**) and protein (**c**) levels of H7N9 HA and GANC were determined by RT-qPCR and Western blotting. **d-f** C57BL/6 mice were infected with 50 μL of H7N9 virus (3 MLD_50_ per mouse). Lung samples were collected at days1 and 4 post-infection, and the mRNA (**d, e**) and protein (**f**) levels of H7N9 HA and GANC were assessed by RT-qPCR and Western blotting. Quantification was shown as mean ± SEM. *n* = 3 or 6 independent experiments. Student’s *t*-test (unpaired, two-tailed) was used to compare two independent groups. ** P <* 0*.*05, ** *P* < 0.01, **** *P* < 0.0001, ns = not significant. MOI: multiplicity of infection; MLD_50_: mouse lethal dose.

Supplementary Fig. 3.


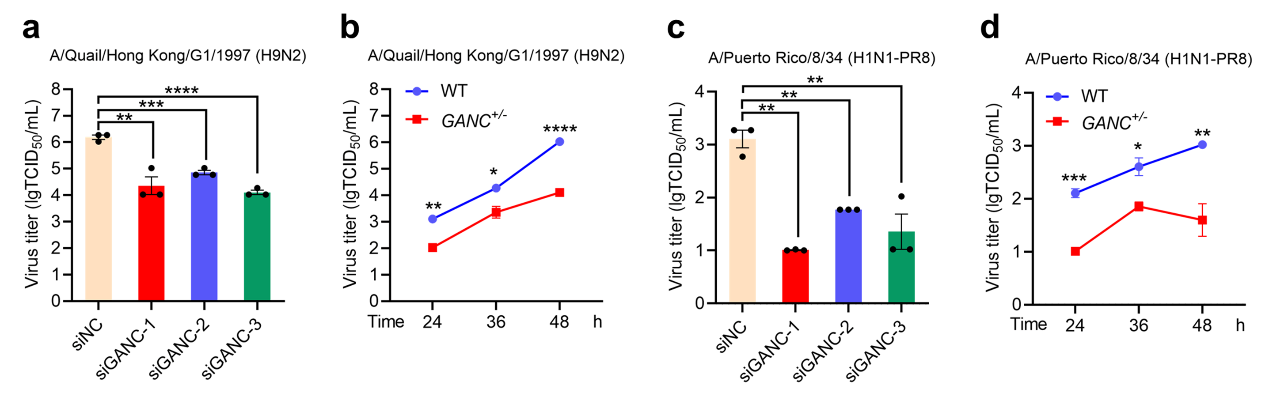


**Supplementary Fig. 3** **GANC knockdown inhibits the replication of H9N2 and H1N1-PR8 viruses (related to Fig. 2).** **a** Virus titers in supernatants from GANC siRNA-transfected A549 cells infected with H9N2 virus at a MOI of 0.1, were determined at 48 h post-infection using TCID_50_ assays in MDCK cells. **b** Virus titers in supernatants from WT A549 and *GANC^+/-^* cells infected with H9N2 virus at a MOI of 0.1, were determined at 24, 36, and 48 h post-infection using TCID_50_ assays in MDCK cells. **c** Virus titers in supernatants from GANC siRNA-transfected A549 cells infected with H1N1-PR8 virus at a MOI of 0.1, were determined at 48 h post-infection using TCID_50_ assays in MDCK cells. **d** Virus titers in supernatants from WT A549 and *GANC^+/-^* cells infected with H1N1-PR8 virus at a MOI of 0.1, were determined at 24, 36, and 48 h post-infection using TCID_50_ assays in MDCK cells. Quantification was shown as mean ± SEM. *n* = 3 independent experiments. Student’s *t*-test (unpaired, two-tailed) was used to compare two independent groups. ** P <* 0*.*05, ** *P* < 0.01, *** *P* < 0.001, **** *P* < 0.0001. WT: wild-type; MOI: multiplicity of infection; TCID_50_: tissue culture infective dose.

Supplementary Fig. 4.

**
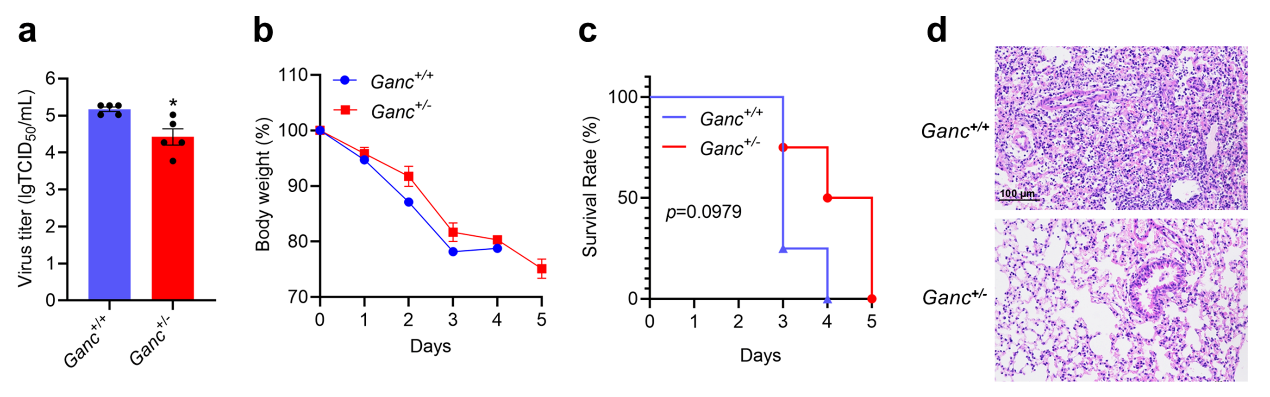
**

**Supplementary Fig. 4 GANC promotes H1N1-PR8 virus replication in vivo (related to Fig. 3).** Six-week-old female C57BL/6N *Ganc^+/-^* and *Ganc^+/+^* mice were intranasally inoculated with 5 MLD_50_ per mouse with H1N1-PR8 virus. Lung tissues were collected from five mice per group at day 4 post-infection for viral titer. Four mice per group were monitored for body weight and survival for 5 days post-infection. **a** Virus titers in the lungs of *Ganc^+/-^* and *Ganc^+/+^* mice were determined by TCID_50_ assays in MDCK cells. **b** Daily monitoring of body weights in infected mice over 5 days. **c** Survival analysis of infected mice, including those humanely sacrificed after losing more than 20% of body weight post-infection. **d** Histopathological analysis of lung lesions. Scale bars, 100 μm. Quantification was shown as mean ± SEM. *n* = 4 or 5 independent experiments. Student’s *t*-test (unpaired, two-tailed) was used to compare two independent groups. * *P* < 0.05. MLD_50_: mouse lethal dose; TCID_50_: tissue culture infective dose.

Supplementary Fig. 5.


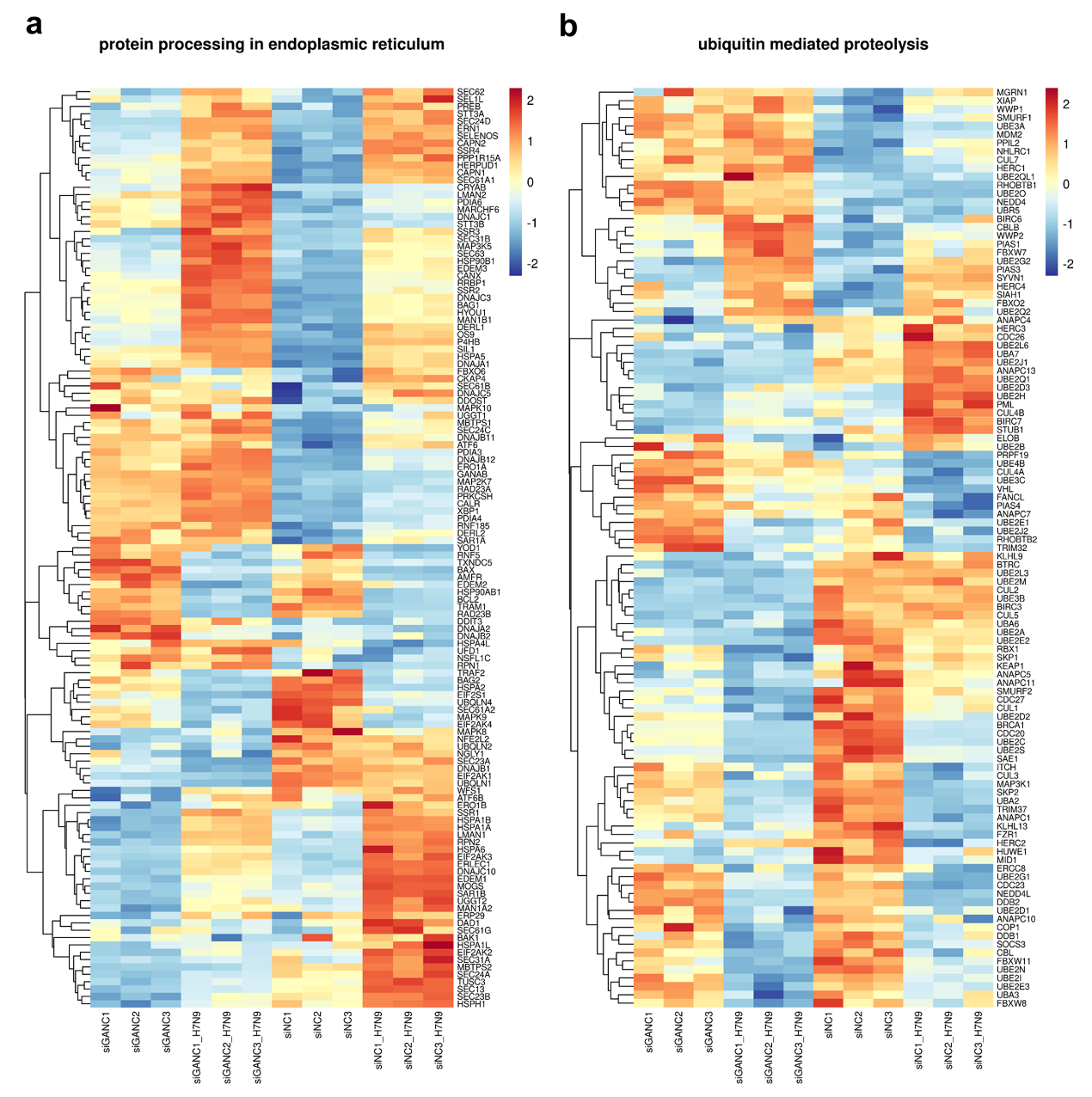


**Supplementary Fig. 5 GANC may regulate protein processing via ubiquitin-mediated proteolysis (related to Fig. 4). a** A heat map showing the mRNA expression levels of DEGs involved in protein processing in the ER. **b** A heat map showing the mRNA expression levels of DEGs involved in ubiquitin-mediated proteolysis. DEG: differentially expressed gene; ER: endoplasmic reticulum.

Supplementary Fig. 6.


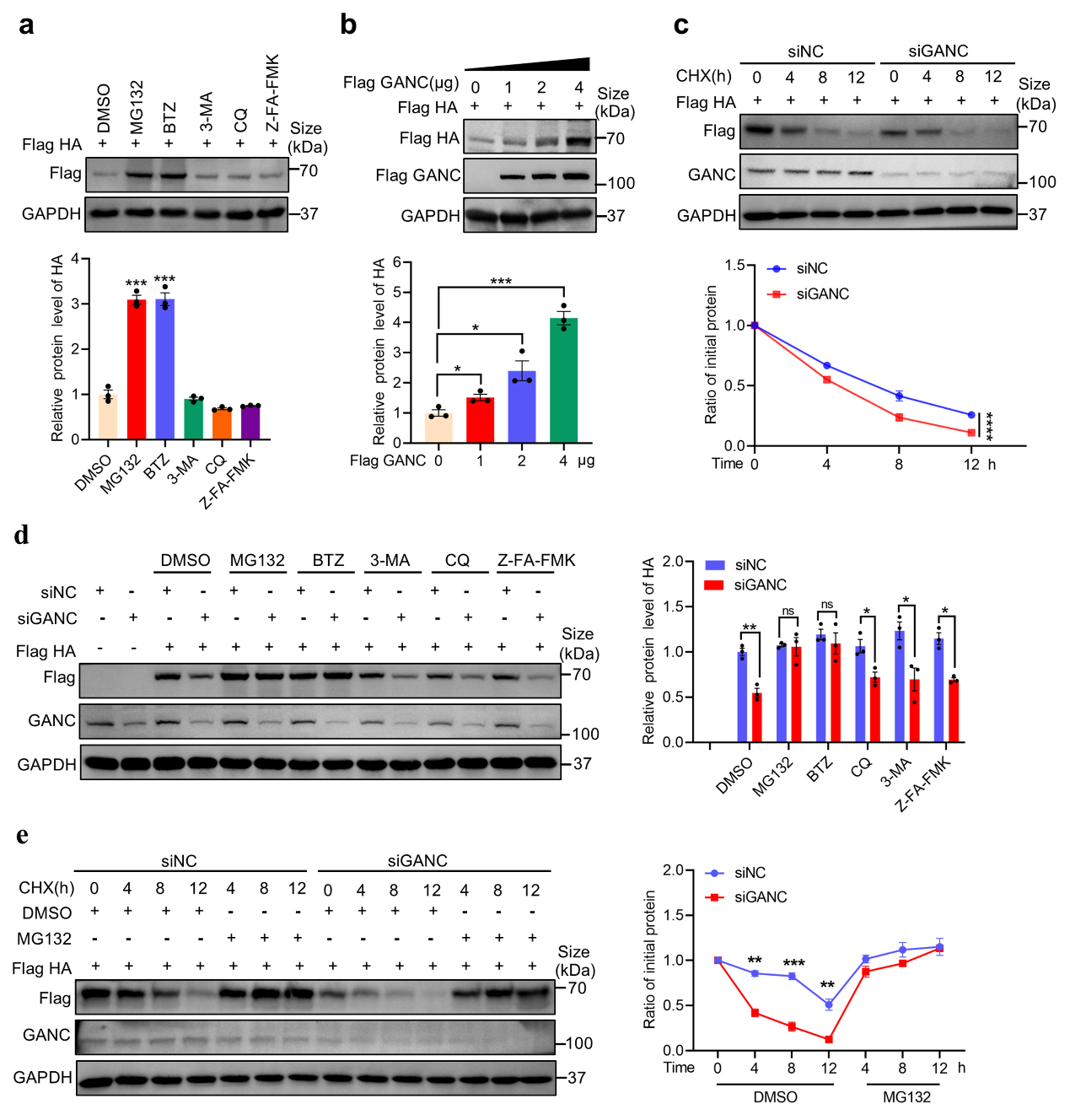


**Supplementary Fig. 6 GANC knockdown increases the proteasome-dependent degradation of HA in HEK293T cells (related to Fig. 5). a** HEK293T cells were transfected with HA plasmids tagged with Flag, treated with DMSO, MG132 (20 μM), BTZ (20 μM), 3-MA (1 mg/mL), CQ (100 μM), or Z-FA-FMK (50 μM) for 12 h before collection. Protein levels of HA were assessed by Western blotting. **b** HEK293T were transfected with HA plasmids tagged with Flag, and cotransfected with increasing amounts of plasmids containing GANC tagged with Flag. Cells were collected 48 h post-transfection for analysis of HA protein levels by Western blotting. **c** HEK293T cells were transfected with GANC siRNA, along with HA plasmids tagged with Flag, treated with CHX (40 μg/mL), and collected at indicated times to assess HA protein levels by Western blotting. **d** HEK293T cells were transfected with GANC siRNA, along with HA plasmids tagged with Flag, and treated with different chemical inhibitors for 12 h before collection. HA protein levels were evaluated by Western blotting. **e** HEK293T cells were transfected with GANC siRNA, along with HA plasmids tagged with Flag, and cotreated with CHX (40 μg/mL) and either DMSO or MG132 (20 μM). Cells were collected at indicated times for Western blotting to analysis HA protein levels. Quantification was shown as mean ± SEM. *n* = 3 independent experiments. Student’s *t*-test (unpaired, two-tailed) was used to compare two independent groups, and a two-way ANOVA test was performed for comparisons of multiple groups. ** P <* 0*.*05, ** *P* < 0.01, *** *P* < 0.001, **** *P* < 0.0001, ns = not significant. CHX: cycloheximide.

Supplementary Fig. 7.


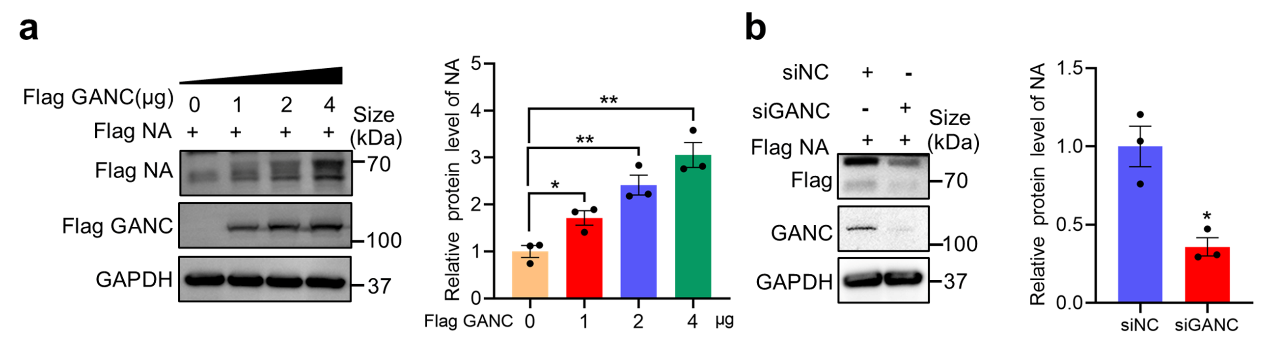


**Supplementary Fig. 7 GANC facilitates the expression of NA (related to Fig. 5). a** Western blotting for the detection of NA protein levels in HEK293T cells transfected with increasing amounts of GANC plasmids tagged with Flag, along with NA plasmids tagged with Flag, cells were collected 48 h post-transfection. **b** Western blotting analysis of NA protein levels in HEK293T cells transfected with siRNA targeting GANC, along with NA plasmids tagged with Flag, cells were collected 48 h post-transfection. Quantification was shown as mean ± SEM. *n* = 3 independent experiments. Student’s *t*-test (unpaired, two-tailed) was used to compare two independent groups, and a two-way ANOVA test was performed for comparisons of multiple groups. ** P <* 0*.*05, ** *P* < 0.01.

Supplementary Fig. 8.

**
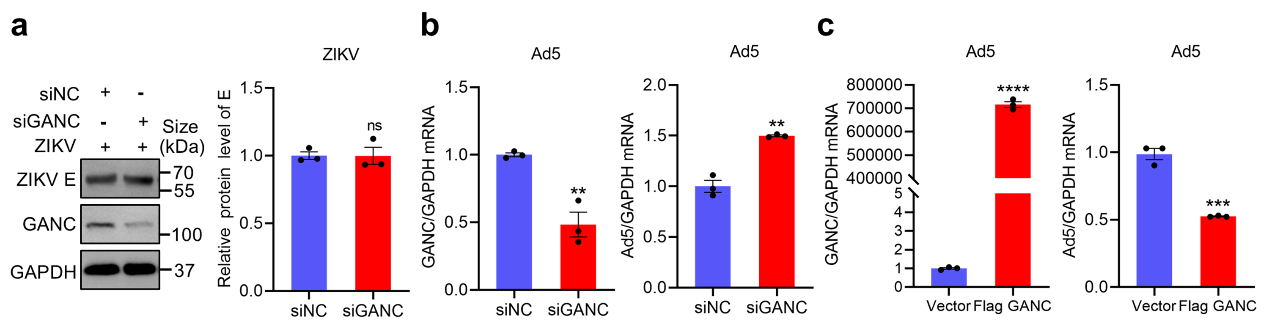
**

**Supplementary Fig. 8 GANC does not affect the expression of the ZIKV E protein, but it effectively inhibited the Ad5 replication (related to Fig. 5). a** The expression levels of ZIKV E from GANC siRNA-transfected A549 cells infected with ZIKV at a MOI of 0.5, were determined at 48 h post-infection by Western blotting. **b** The viral DNA in cells from GANC siRNA-transfected A549 cells infected with Ad-5 at a MOI of 0.5, were determined at 48 h post-infection by RT-qPCR. **c** The viral DNA in cells from GANC plasmids-transfected A549 cells infected with Ad-5 at a MOI of 0.5, were determined at 48 h post-infection by RT-qPCR. Quantification was shown as mean ± SEM. *n* = 3 independent experiments. Student’s *t*-test (unpaired, two-tailed) was used to compare two independent groups. ** *P* < 0.01, *** *P* < 0.001, **** *P* < 0.0001, ns = not significant. ZIKV: Zika virus; MOI: multiplicity of infection; Ad-5: Adenovirus 5.

Supplementary Fig. 9.


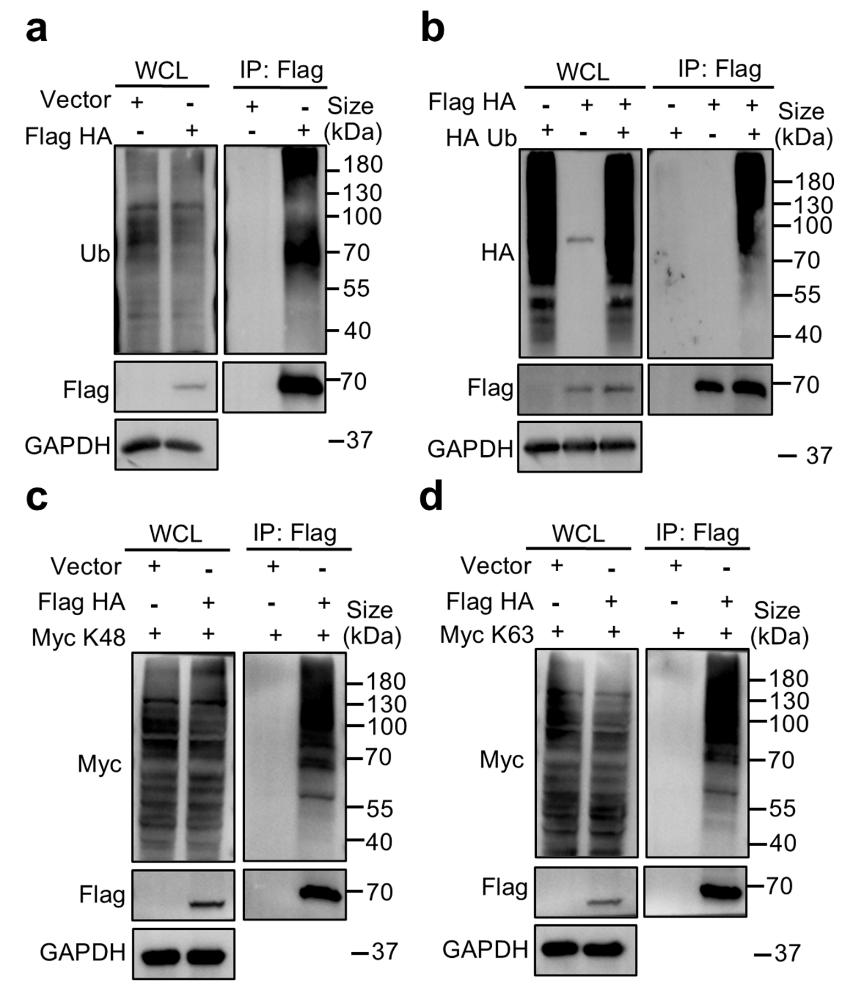


**Supplementary Fig. 9 HA interacts with ubiquitin (related to Fig. 6). a** Western blotting for the detection of HA ubiquitination levels in HEK293T cells transfected HA plasmids tagged with Flag. The WCLs were incubated with anti-Flag magnetic beads. **b** HEK293T cells were co-transfected with HA plasmids tagged with Flag and ubiquitin plasmids with HA tag. The WCLs were incubated with anti-Flag magnetic beads and subjected to Western blotting. **c, d** HEK293T cells were co-transfected with HA plasmids tagged with Flag, and Myc K48- (**c**) or Myc K63- (**d**) ubiquitin plasmids. The WCLs were incubated with anti-Flag magnetic beads and analyzed by Western blotting. WCL: whole-cell lysate.

Supplementary Fig. 10.


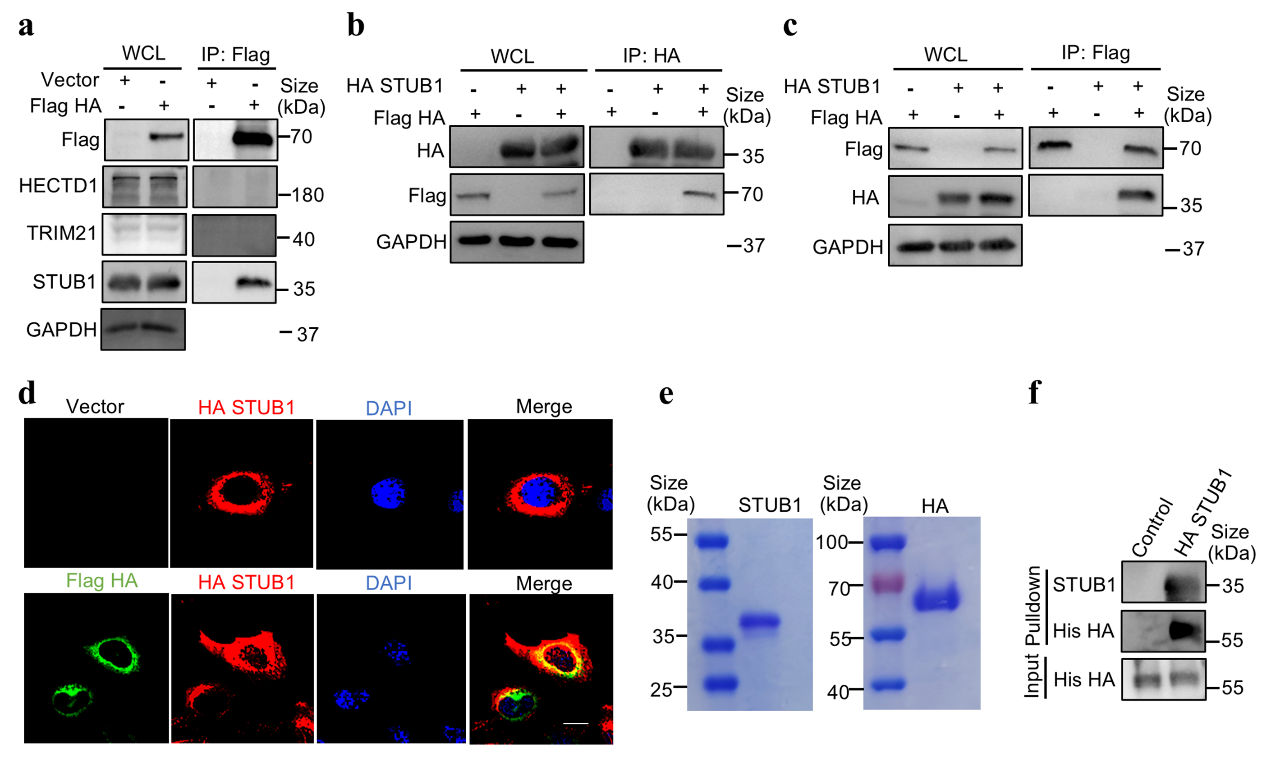


**Supplementary Fig. 10 HA interacts with STUB1 (related to Fig. 6). a** Western blotting for the detection of HECTD1, TRIM21, and STUB1 in HEK293T cells transfected with HA plasmids tagged with Flag, the WCLs were incubated with anti-Flag magnetic beads. **b, c** HEK293T cells were transfected with H7N9 HA plasmids tagged with Flag, and STUB1 plasmids tagged with HA. After 48 h, the WCLs were incubated with anti-HA magnetic beads (**b**) or anti-Flag magnetic beads **(c)** and subjected to Western blotting. **d** Colocalization of HA and STUB1 at 24 h post-transfection in HeLa cells transfected with the indicated plasmids. Scale bars, 20 μm. **e** Coomassie staining of purified STUB1 and HA proteins. **f** Anti-HA magnetic beads binding STUB1 proteins tagged with HA, incubated with HA proteins tagged with His, and the pulldown proteins were detected with anti-His tag antibody by Western blotting. WCL: whole-cell lysate.

Supplementary Fig. 11.

**
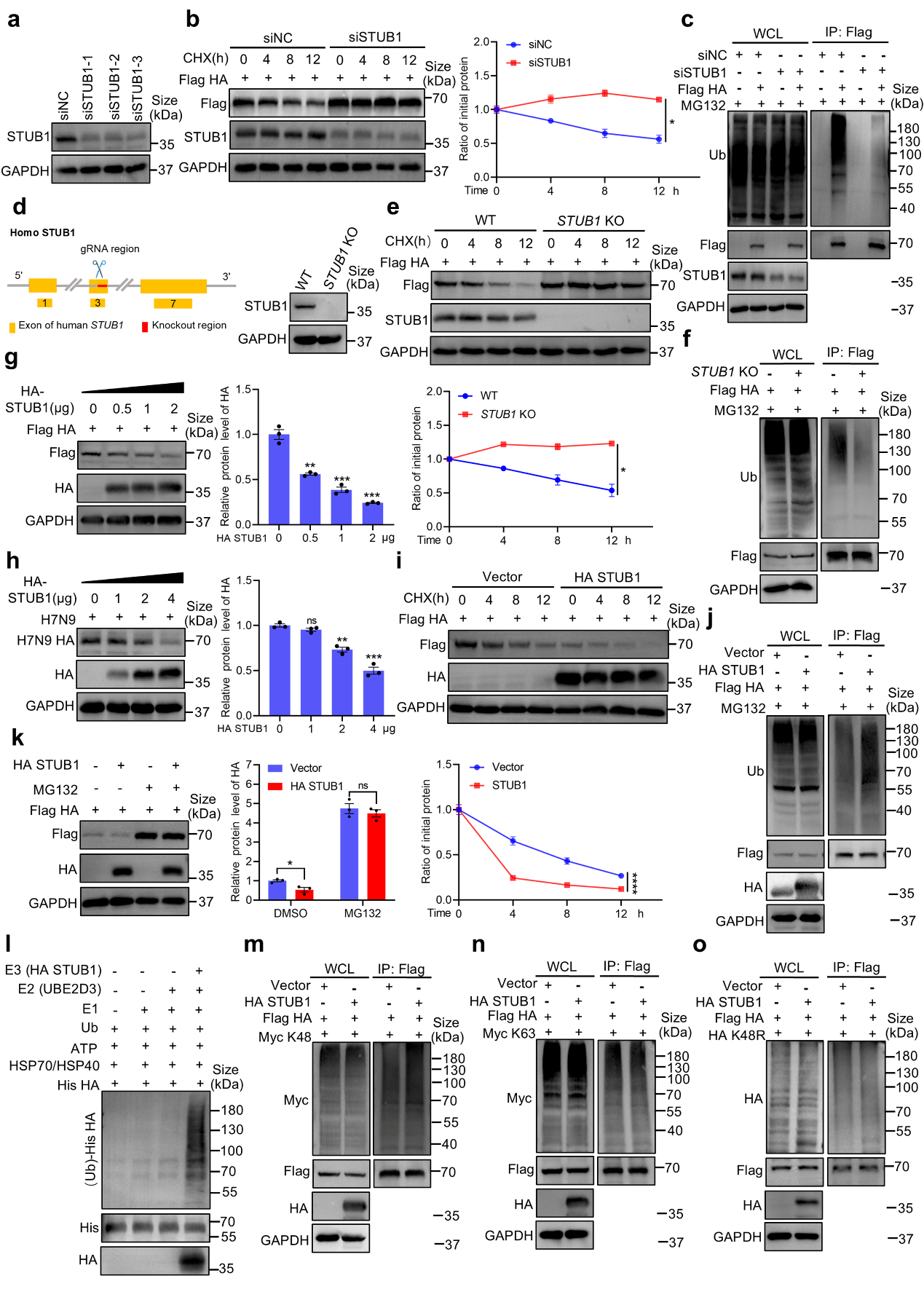
**

**Supplementary Fig. 11** **STUB1 mediates the degradation and ubiquitination of HA (related to Fig. 6). a** Western blotting analysis of STUB1 from HEK293T cells transfected with STUB1 siRNA. **b** HEK293T cells were transfected with STUB1 siRNA, along with HA plasmids tagged with Flag. Cells were treated with CHX (40 μg/mL) and collected at the indicated times to assess the protein levels of HA by Western blotting. **c** Analysis of HA ubiquitination in STUB1 siRNA-transfected HEK293T cells, and transfected with Flag HA or not. The polyubiquitin chains of HA were detected by Western blotting. **d** The scheme of sgRNA was used for *STUB1* gene knockout, and Western blotting for endogenous STUB1 protein in knockout HEK293T cells. **e** WT and STUB1 knockout HEK293T cells were transfected with HA plasmids tagged with Flag, treated with CHX (40 μg/mL), and collected at the indicated times to test the protein levels of HA by Western blotting. **f** Detection of polyubiquitin chains on HA in WT and STUB1 knockout HEK293T cells treated with MG132 using anti-Flag magnetic beads. **g** Western blotting for the detection of HA protein levels in HEK293T cells transfected with increasing amounts of STUB1 plasmids tagged with HA. **h** A549 cells were transfected with STUB1 plasmids tagged with HA, and then infected with H7N9 virus at a MOI of 0.1. Cells were collected for analysis of HA protein levels by Western blotting. **i** HEK293T cells were transfected with HA plasmids tagged with Flag, together with STUB1 plasmids tagged with HA, treated with CHX (40 μg/mL), and collected at indicated times to test HA protein levels by Western blotting. **j** HEK293T cells were transfected with HA plasmids tagged with Flag, together with STUB1 plasmids tagged with HA, treated with MG132 (20 μM) for 12 h, and collected at indicated times to test polyubiquitin chains of HA by Western blotting. **k** HEK293T cells were transfected with HA plasmids tagged with Flag, and co-transfected with increasing amounts of STUB1 plasmids tagged with HA, and were treated with MG132 (20 mM) for 12 h. Cells were collected for analysis of HA protein levels by Western blotting. **l** In vitro ubiquitination assay of HA. STUB1 protein tagged with HA were incubated with HA tagged with His in a reaction buffer containing ATP, ubiquitin, E1 (UBE1) and E2 (UbcH5c/UBE2D3). The ubiquitination levels were detected by Western blotting with anti-Ub antibody. **m-o** HEK293T cells were co-transfected with HA plasmids tagged with Flag, STUB1 plasmids tagged with HA, and K48- (**m**), K63- (**n**) ubiquitin plasmids tagged with Myc, or K48R- (**o**) ubiquitin plasmids tagged with HA. Cells were treated with MG132 (20 μM) for 12 h before collection. The WCLs were incubated with anti-Flag magnetic beads and analyzed by Western blotting. Quantification was shown as mean ± SEM. *n* = 3 independent experiments. Student’s *t*-test (unpaired, two-tailed) was used to compare two independent groups, and a two-way ANOVA test was performed for comparisons of multiple groups. ** P <* 0*.*05, ** *P* < 0.01, *** *P* < 0.001, **** *P* < 0.0001, ns = not significant. CHX: cycloheximide. WCL: whole-cell lysate.

Supplementary Fig. 12.


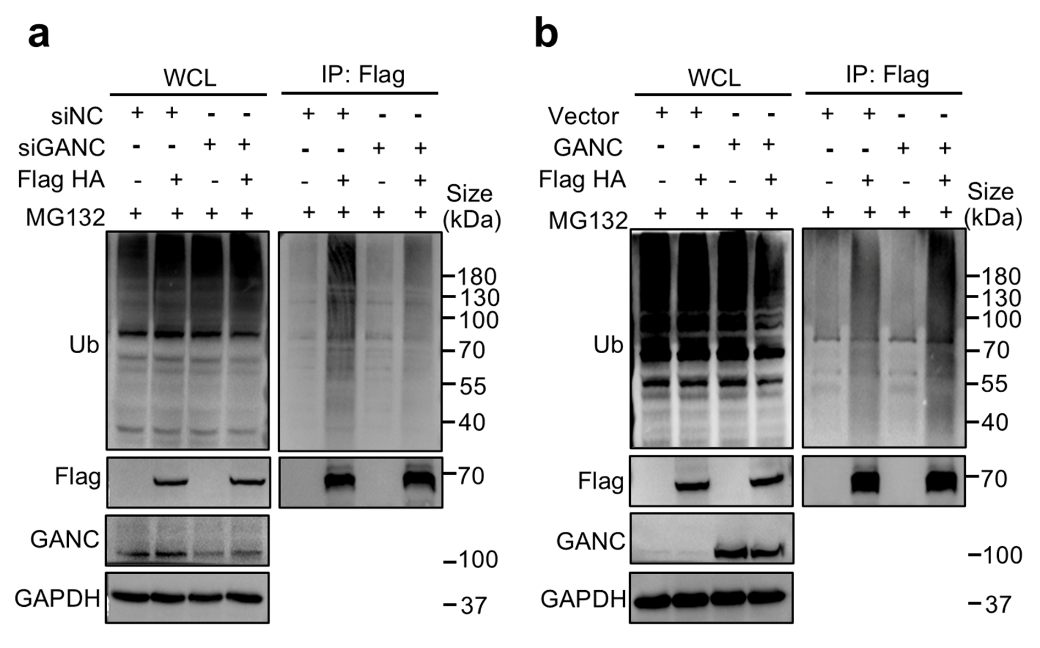


**Supplementary Fig. 12 GANC increases the polyubiquitination of HA (related to Fig. 6). a, b** HEK293T cells were transfected with GANC siRNA (**a)** or GANC plasmids (**b)**, along with HA plasmids tagged with Flag, treated with MG132 (20 μM) for 12 h, and cells were collected at indicated times to test polyubiquitin chains of HA by Western blotting. WCL: whole-cell lysate.

Supplementary Fig. 13.


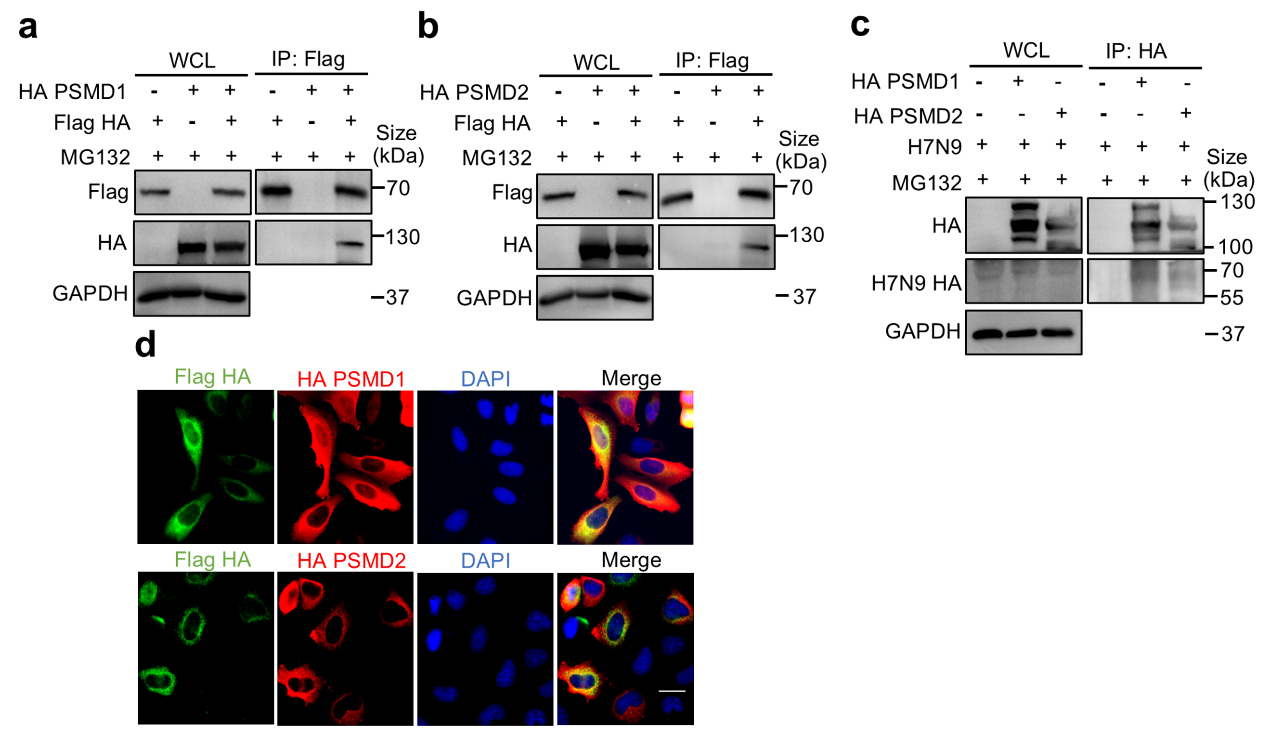


**Supplementary Fig. 13 HA interacts with PSMD1 or PSMD2 (related to Fig. 6).** **a, b** HEK293T cells were transfected with HA plasmids tagged with Flag, along with PSMD1 (**a**) or PSMD2 (**b**) plasmids tagged with HA, treated with MG132 (20 μM) for 12 h before collection. The WCLs were incubated with anti-Flag magnetic beads and used for Western blotting. **c** HEK293T cells were transfected with PSMD1 or PSMD2 plasmids tagged with HA, and infected with H7N9 virus, treated with MG132 (20 μM) for 12 h before collection. The WCLs were incubated with anti-HA magnetic beads and subjected to Western blotting. **d** Colocalization of HA and PSMD1 or PSMD2 at 24 h post-transfection in HeLa cells transfected with the indicated plasmids. Scale bars, 20 μm. WCL: whole-cell lysate.

Supplementary Fig. 14.


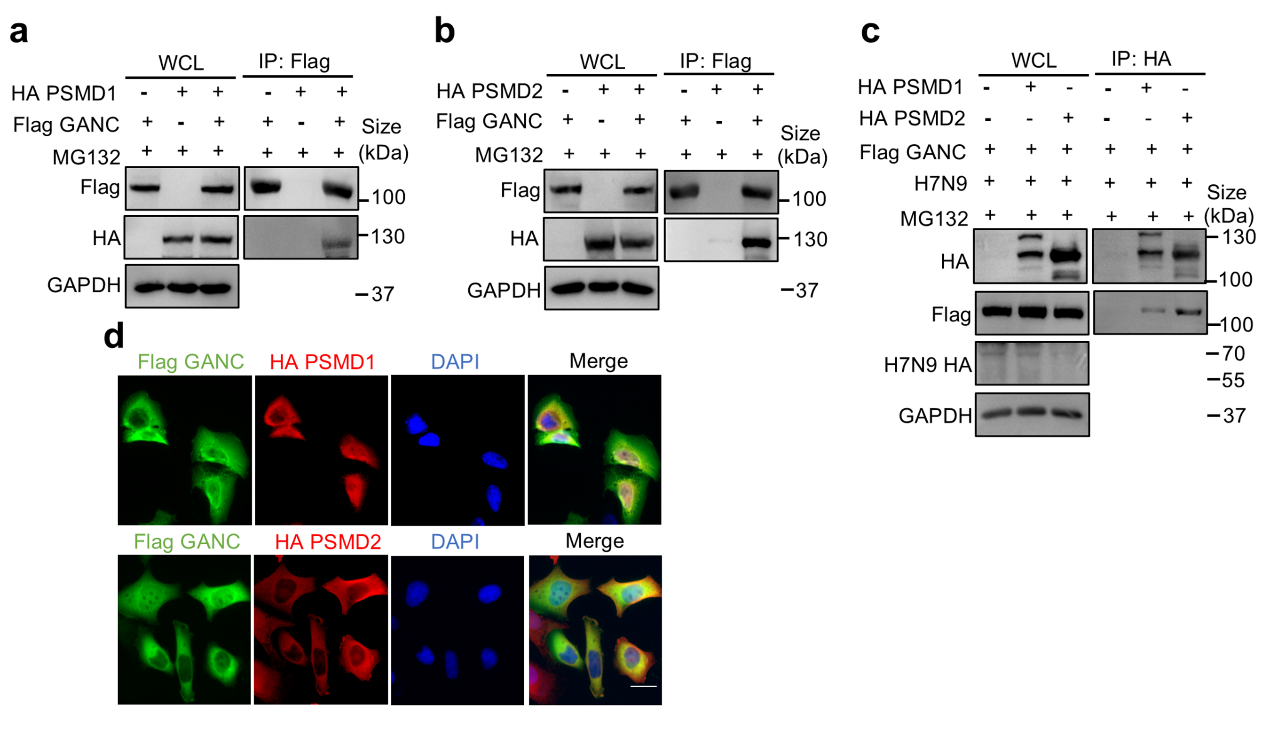


**Supplementary Fig. 14 GANC interacts with PSMD1 or PSMD2 (related to Fig. 7).** **a, b** HEK293T cells were transfected with GANC plasmids tagged with Flag, along with PSMD1 (**a**) or PSMD2 (**b**) plasmids tagged with HA, treated with MG132 (20 μM) for 12 h before collection. The WCLs were incubated with anti-Flag magnetic beads and used for Western blotting. **c** HEK293T cells were transfected with PSMD1 or PSMD2 plasmids tagged with HA, and GANC plsamids tagged with Flag, and infected with H7N9 virus, treated with MG132 (20 μM) for 12 h before collection. The WCLs were incubated with anti-HA magnetic beads and subjected to Western blotting. **d** Colocalization of GANC and PSMD1 or PSMD2 at 24 h post-transfection in HeLa cells transfected with the indicated plasmids. Scale bars, 20 μm. WCL: whole-cell lysate.

Supplementary Fig. 15.


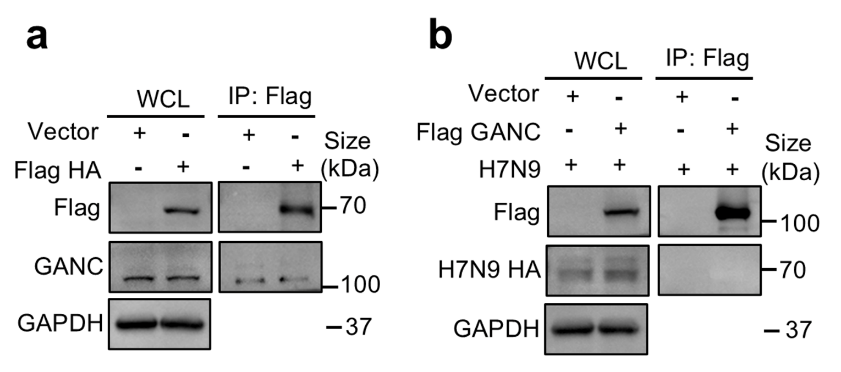


**Supplementary Fig. 15 HA and GANC does not interact (related to Fig. 7). a** HEK293T cells were transfected with HA plasmids tagged with Flag. 48 h later, the WCLs were incubated with anti-Flag magnetic beads, and subjected to Western blotting to detect GANC. **b** HEK293T cells were transfected with GANC plasmids tagged with Flag, and infected with H7N9 virus. 48 h later, the WCLs were incubated with anti-Flag magnetic beads and subjected to Western blotting to detect HA. WCL: whole-cell lysate.

**Supplementary Table. 1.**

**Carrier frequencies of low-frequency variants of *GANC* gene in H7N9 infections and healthy controls.**

| Sample group | N | Carriers (%) | OR (95% CI)$\mathbf{*}$ | *P* value† |
| --- | --- | --- | --- | --- |
| H7N9 infections | 217 | 29 (13.26) |  |  |
| Healthy controls | 116 | 3 (2.65) | 5.81 (1.73, 19.51) | 0.0008 |

N, number of individuals; OR, odds ratio; CI, confidence interval.

*OR for H7N9 patients compared with control groups. †*P* values were calculated using (two-sided) *Fisher’s* exact test.

**Supplementary Table. 2.**

**Five low-frequency variants of *GANC* gene in H7N9 infections and healthy controls.**

| **dbSNP^#^** | **Position*** | **Nucleotide Change†** | **Amino Acid Change†** | **Type of mutation** | **State** | **Case (217)** | **Control (116)** | **gnomAD (8624 EAS)^$^** |
| --- | --- | --- | --- | --- | --- | --- | --- | --- |
| rs745346596 | 15:42585105 | c.503_506del | p.H168fs | frameshift | Het | 1 | 0 | 0.0003 |
| Novel | 15:42614101 | c.T1176G | p.Y392X | nonsense | Het | 1 | 0 | NA |
| rs768140579 | 15:42614169 | c.1245delC | p.F415fs | frameshift | Het | 1 | 0 | 0.0004 |
| rs188658053 | 15:42631978 | c.G1955A | p.R652Q | missense | Het | 1 | 0 | 0.0024 |
| rs79004308 | 15:42640302 | c.T2306A | p.I769N | missense | Het | 25 | 3 | 0.0366 |

#dbSNP version 150. * NCBI Build 37. † Variations are based on RefSeq records NM_198141.3 and NP_937784.2. $ The allele frequency from the gnomAD EAS exome database. Het, heterozygous.

**Supplementary Table. 3.**

**The sequences of identified peptides of STUB1, HECTD1, and TRIM21.**

| **Identified peptides of STUB1**  **HA** | **Identified peptides of HECTD1**  **HA** | **Identified peptides of**  **TRIM21**  **HA** |
| --- | --- | --- |
| LNFGDDIPSALR | TALENLIVLLK | LAQQSQALQELISELDR |
|  | STTGAPSTTADSK | LGDTQQSIPGNEER |
|  |  | DLDITSPELR |
|  |  | NFLVEEEQR |
|  |  | IHAEFVQQK |
|  |  | GGGSVCPVCR |
|  |  | LQVALGELR |

**Supplementary Table. 4.**

**The sequences of identified peptides of PSMD1 and PSMD2.**

| **Identified peptides of PSMD1**  **HA** | **Identified peptides of PSMD2**  **HA** | |
| --- | --- | --- |
| LLHVAVSDVNDDVR | | VGQAVDVVGQAGKPK |
| TPEASPEPK | | EDVLTLLLPVMGDSK |
|  | | AVPLALALISVSNPR |

**Supplementary Table. 5.**

**The sequences of identified peptides of PSMD1 and PSMD2.**

| **Identified peptides of PSMD1**  **GANC** | **Identified peptides of PSMD2**  **GANC** |  |
| --- | --- | --- |
| QFAALVASK | FGGSGSQVDSAR | |
| VSTAVLSITAK | LNILDTLSK | |
| QAIGIALETR | LVGSQEELASWGHEYVR | |
| TSSAFVGK | VGQAVDVVGQAGKPK | |
|  | YLYSSEDYIK | |
|  | HLAGEVAK | |

**Supplementary Table. 6.**

**The primers for detecting mRNA level of genes.**

| **Target gene** | **Primer sequence (5’-3’)** |
| --- | --- |
| *Human GAPDH* | Forward: GCCTCCTGCACCACCAACTG |
|  | Reverse: ACGCCTGCTTCACCACCTTC |
| *Mouse Gapdh* | Forward: AGGTCGGTGTGAACGGATTTG |
|  | Reverse: TGTAGACCATGTAGTTGAGGTCA |
| *Human GANC* | Forward: AGGCGACAACACAGCAGAATGG |
|  | Reverse: CTCTGGATTCCCAATGAACCCG |
| *Mouse Ganc* | Forward: ACTGTGGCAAGATTGCATTCT |
|  | Reverse: ACTTCATACCTTGGTTTCAGTGG |
| *H7N9 HA* | Forward: CCCARGATCTGCTCAAAAGG |
|  | Reverse: TTGTTCYCAGAAGTCCACATTG |
| *Ad-5* | Forward: GAGTTGGCACCCCTATTCGA |
|  | Reverse: GTTGCTGTGGTCGTTCTGGT |
