# Supplementary material for: Glucosidase alpha neutral C promotes influenza virus replication by inhibiting proteosome-dependent degradation of hemagglutinin
Source: Signal Transduct Target Ther. 2025 Apr 23;10:131. doi: 10.1038/s41392-025-02227-6 (PMC12015365; doi:10.1038/s41392-025-02227-6)

Uncropped Western Blots

Fig. 1d

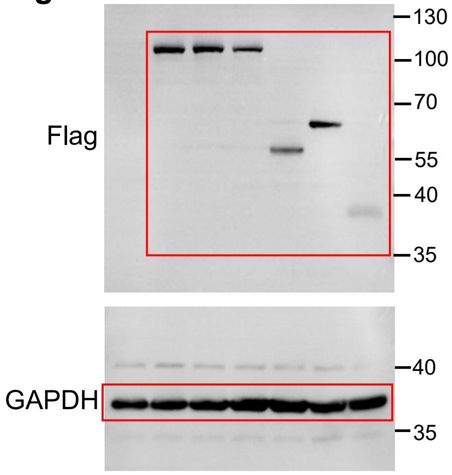

Fig. 2a

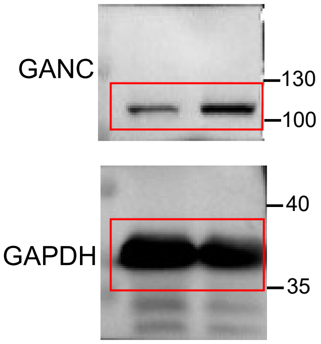

Fig. 2d

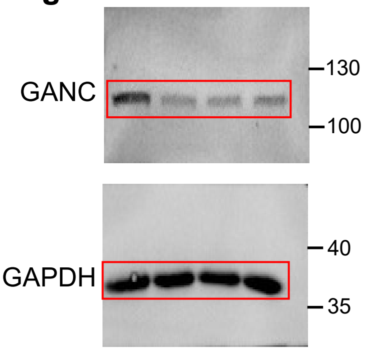

Fig. 2j

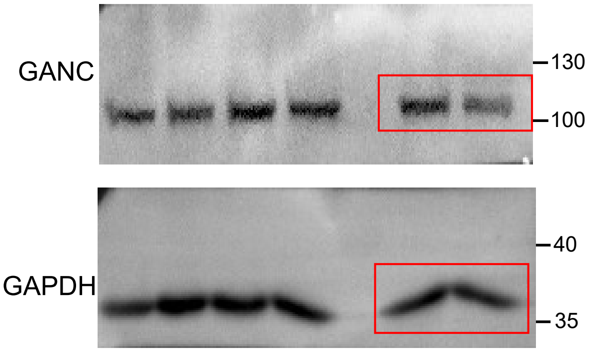

Fig. 3b

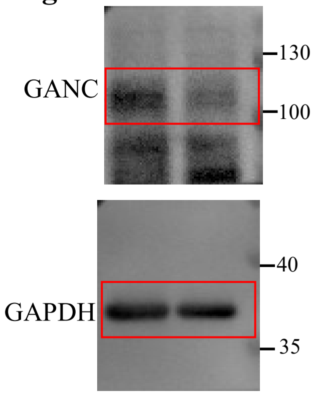

Fig. 4f

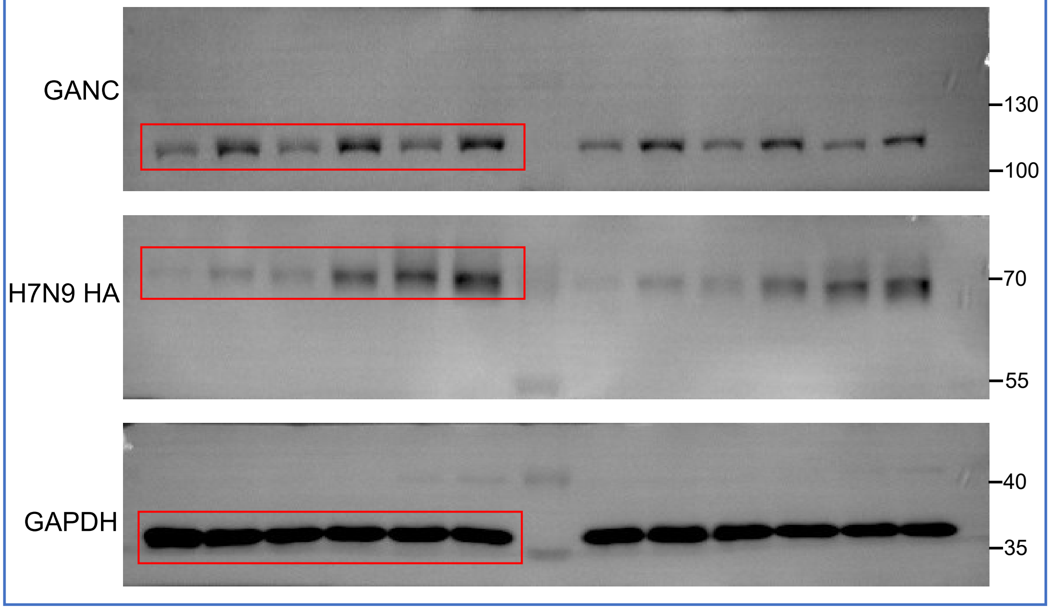

Fig. 4g

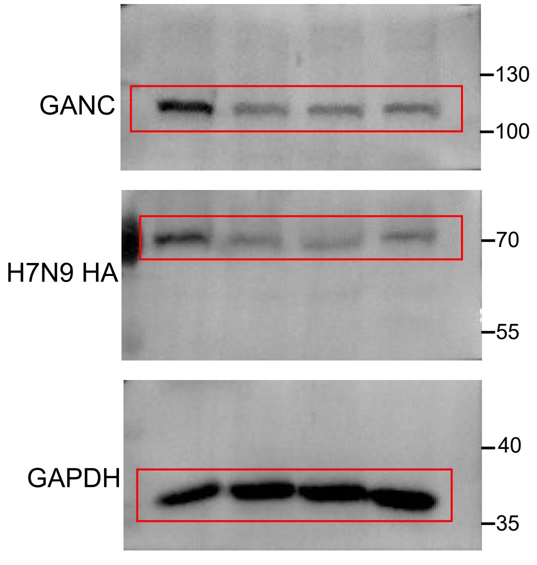

Fig. 4h

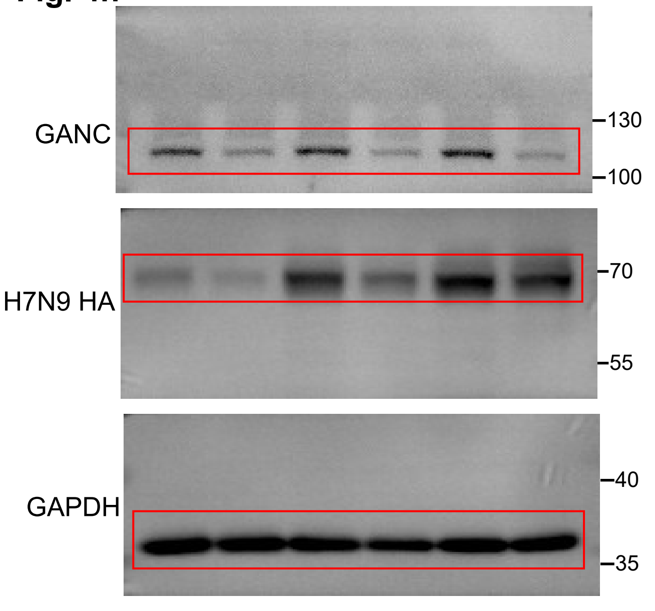

Fig. 4i

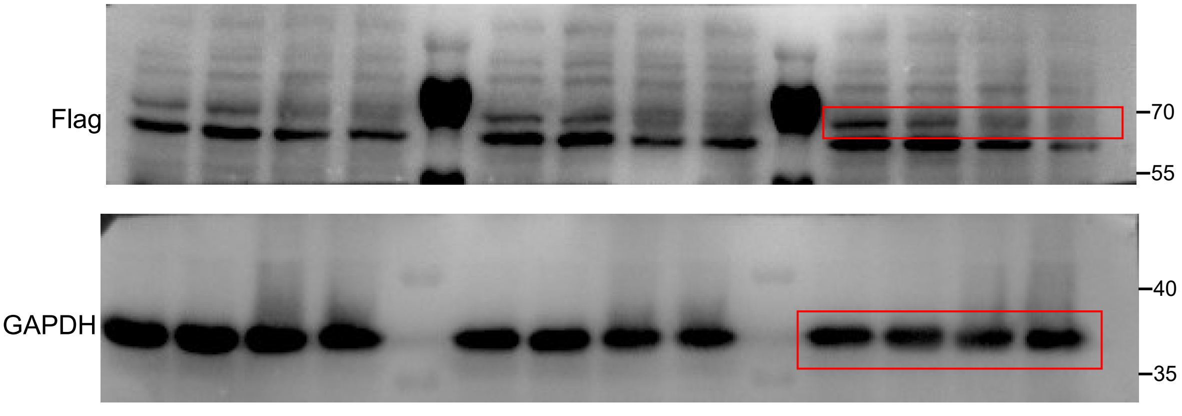

Fig. 5a

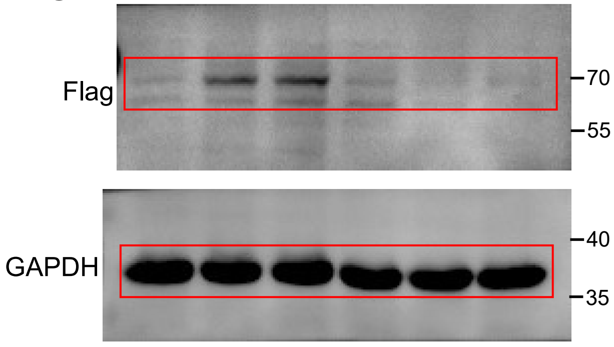

Fig. 5b

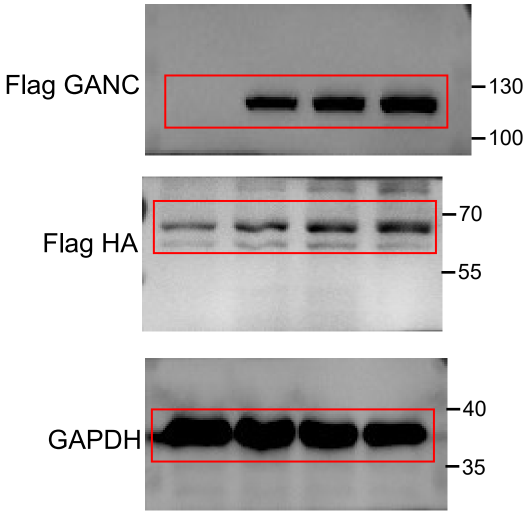

Fig. 5c

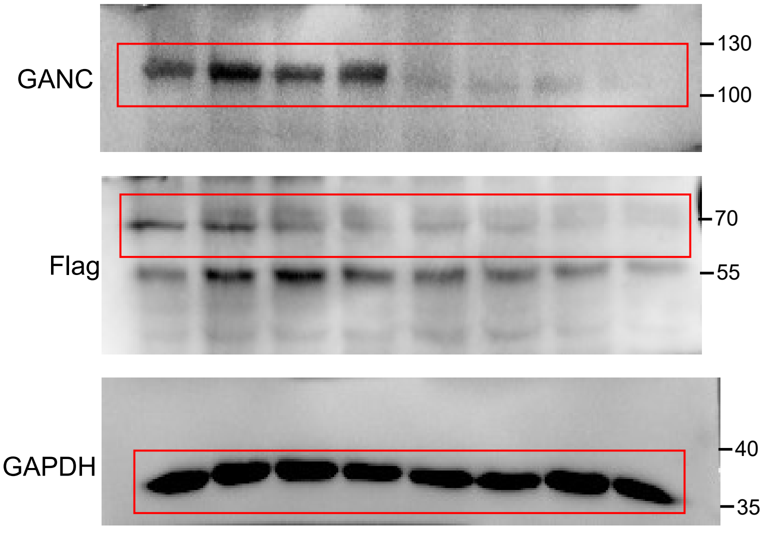

Fig. 5d

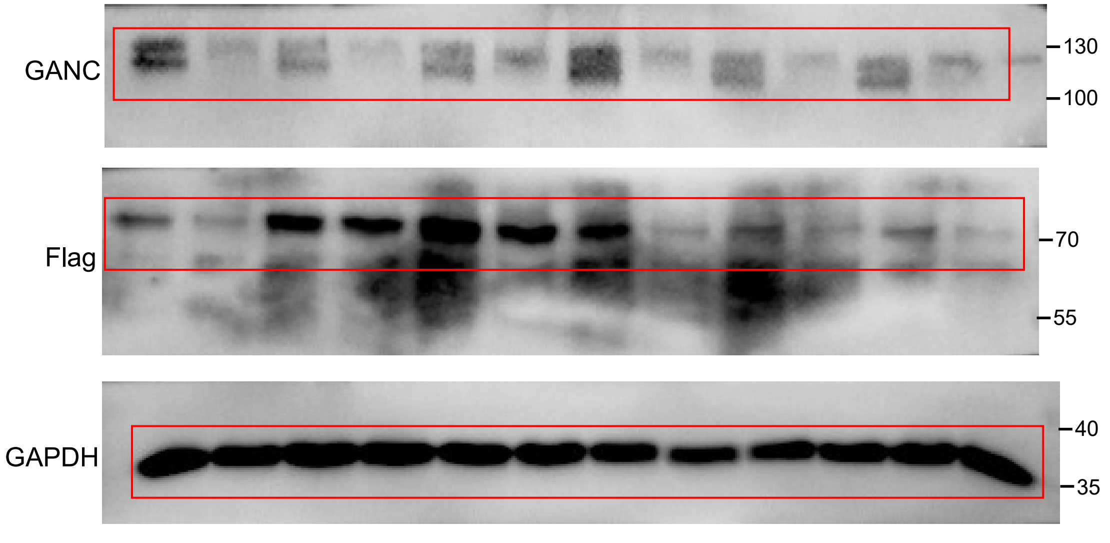

Fig. 5e

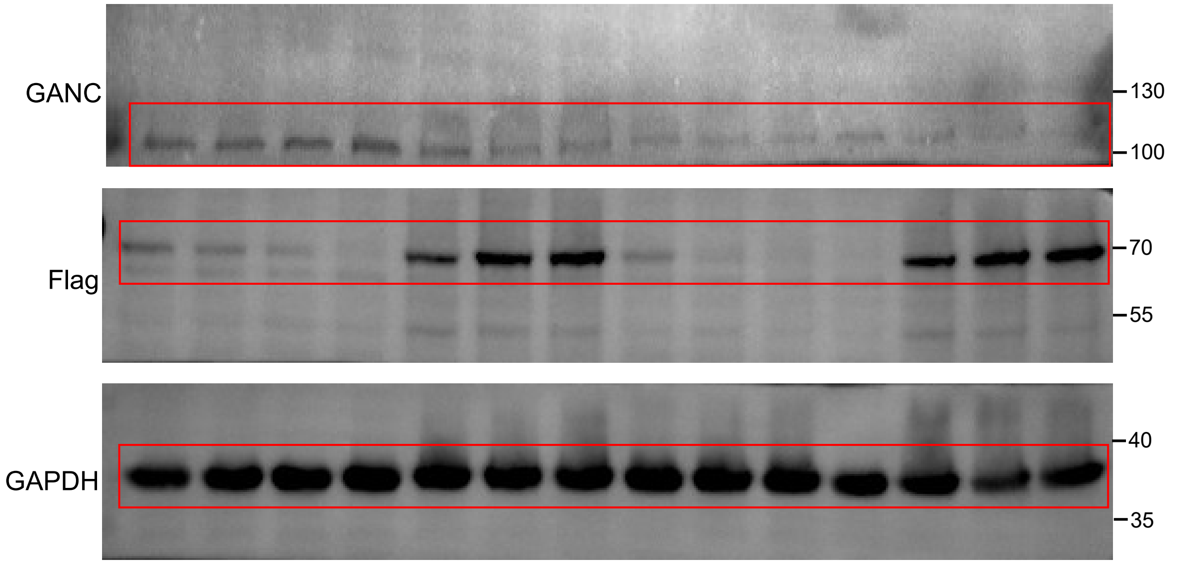

Fig. 5f

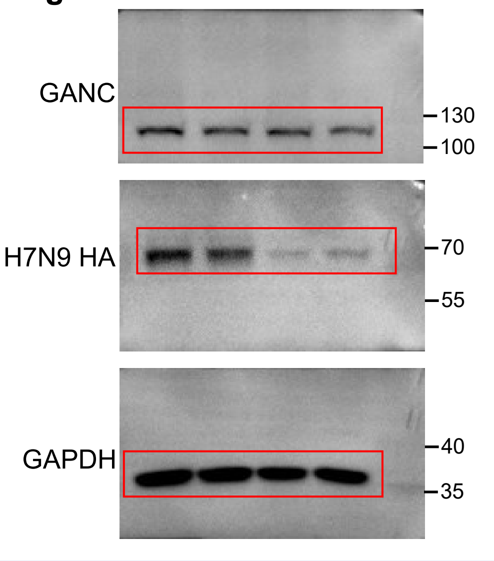

Fig. 6b

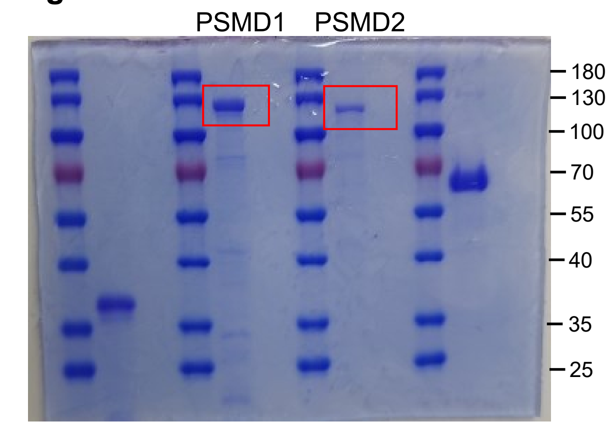

Fig. 6a

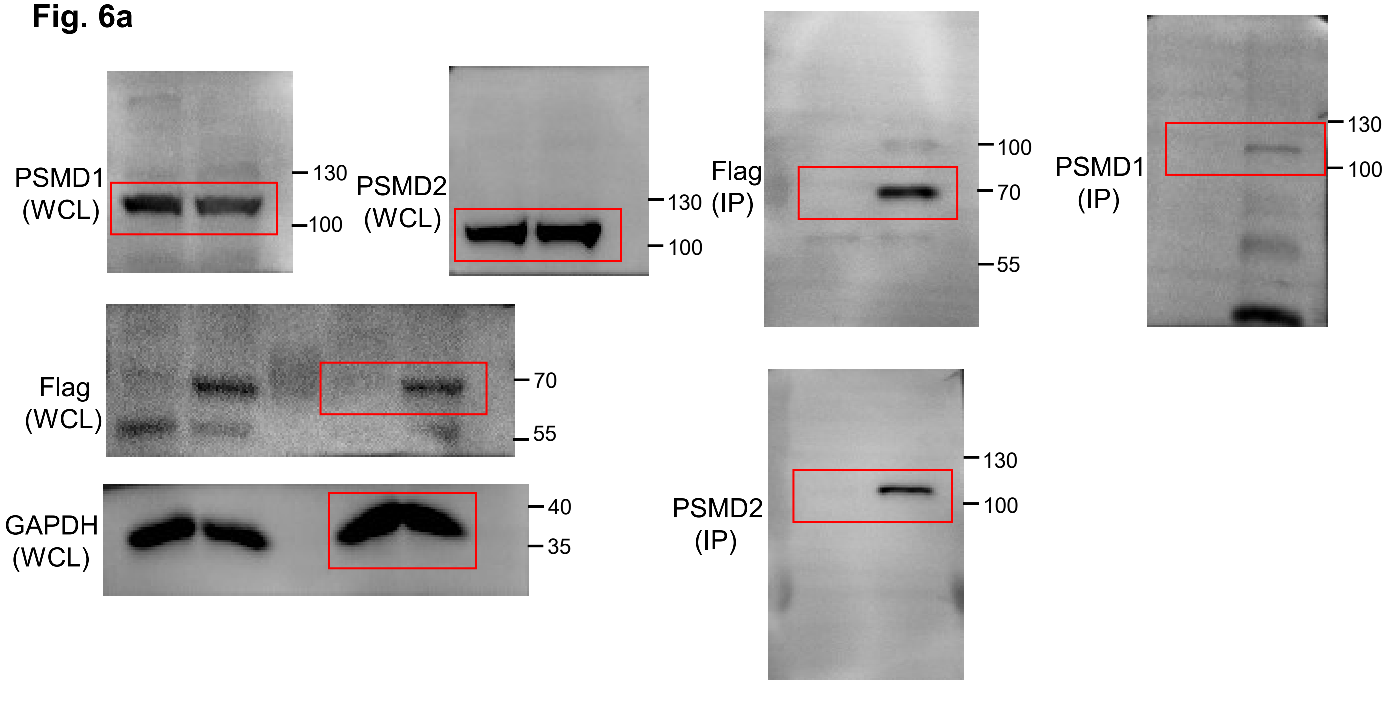

Fig. 6c

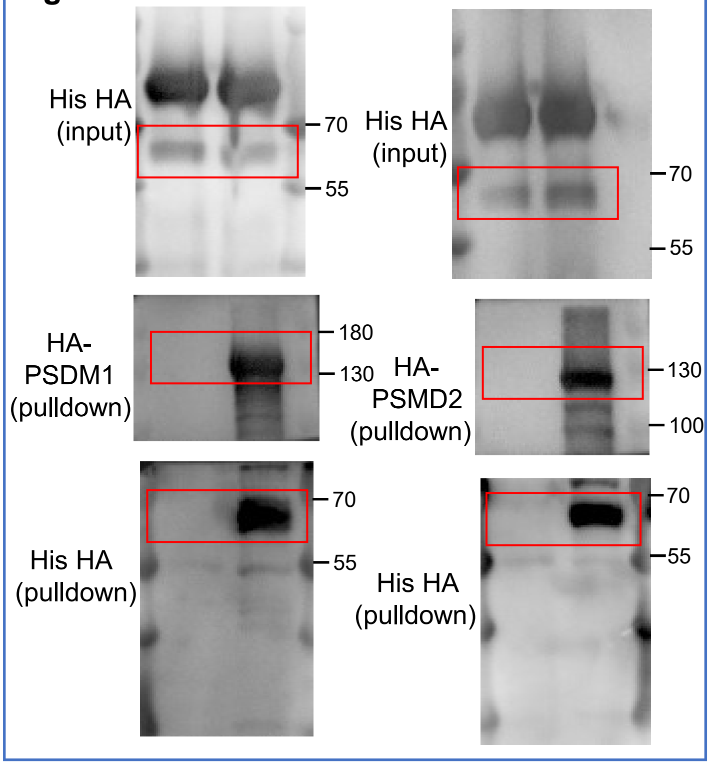

Fig. 6d

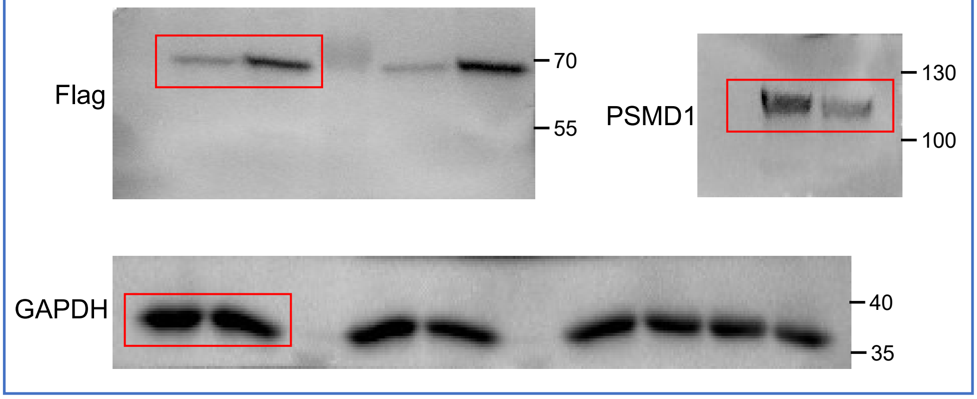

Fig. 6e

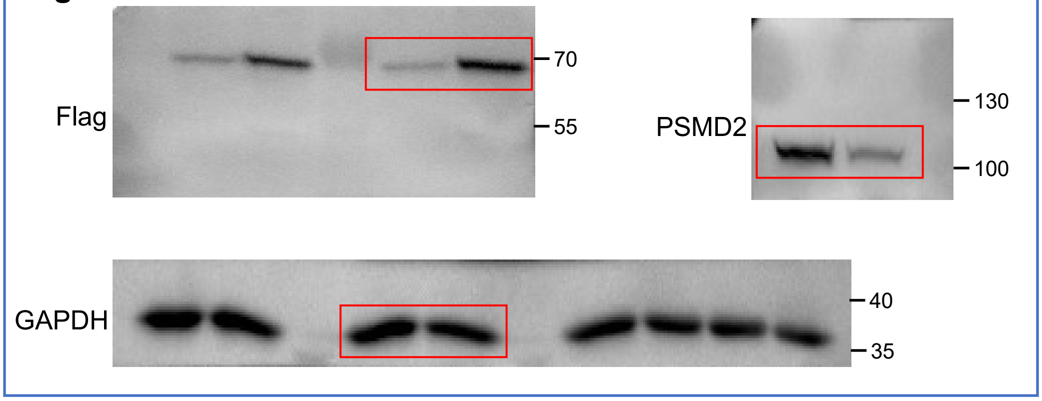

Fig. 6f

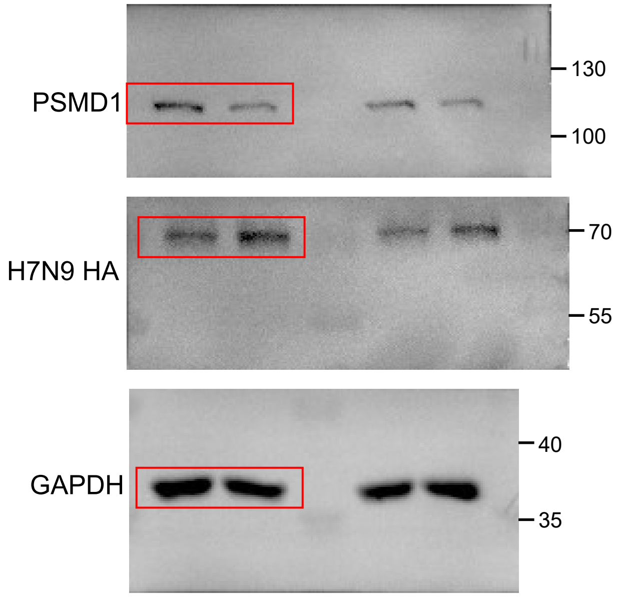

Fig. 6g

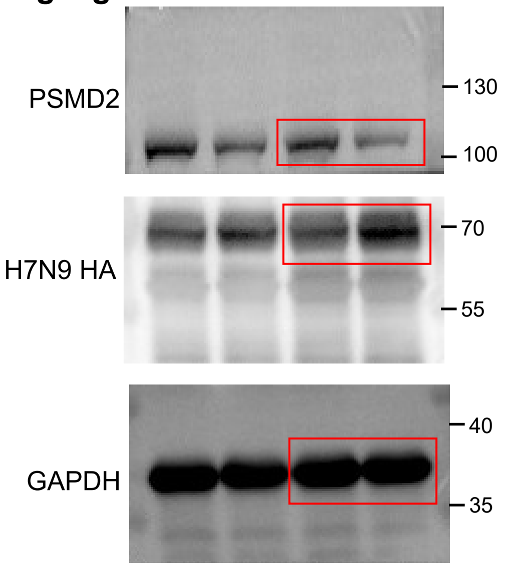

Fig. 6h

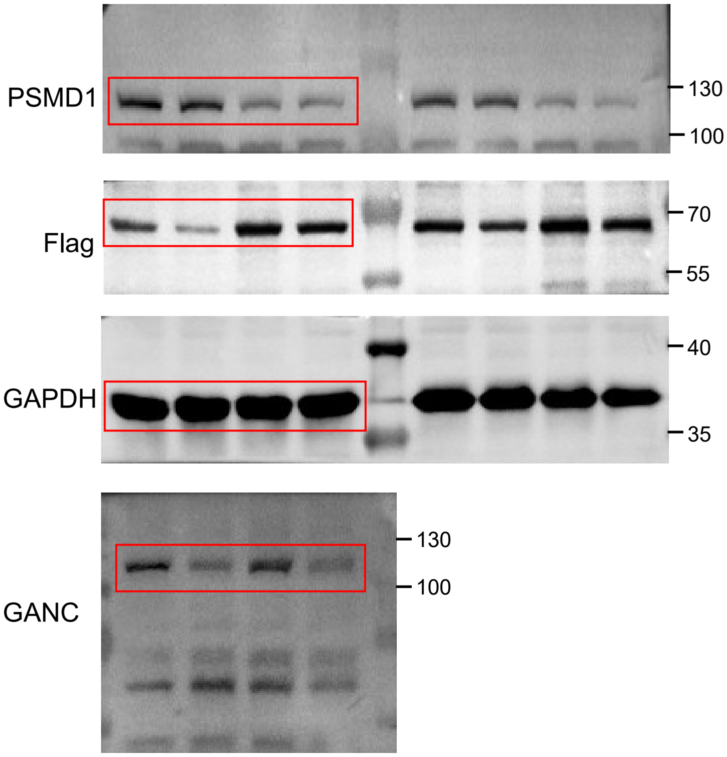

Fig. 6i

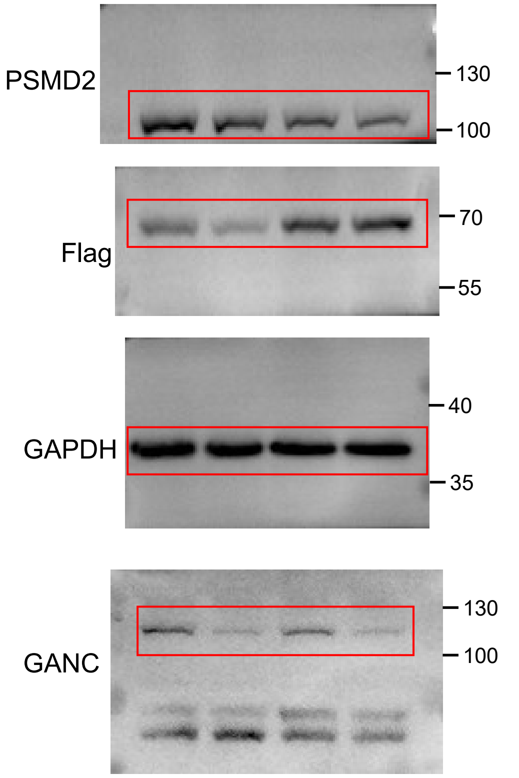

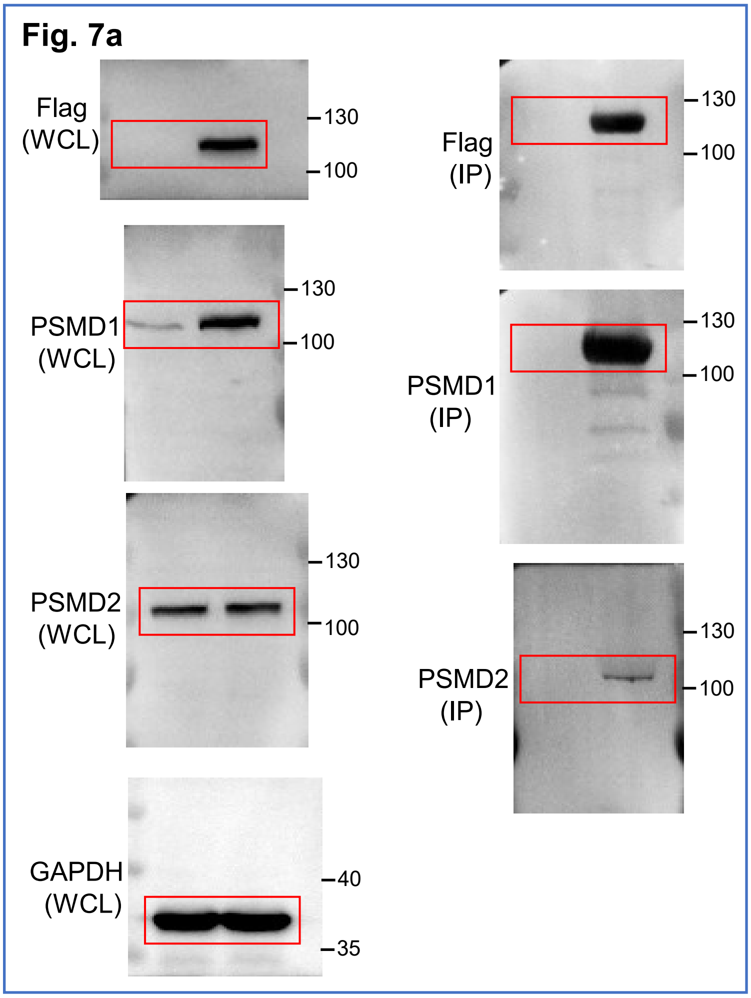
Fig. 7a
Flag (WCL)
130
100
PSMD1 (WCL)
130
100
PSMD2 (WCL)
130
100
GAPDH (WCL)
40
35
Flag (IP)
130
100
PSMD1 (IP)
130
100
PSMD2 (IP)
130
100

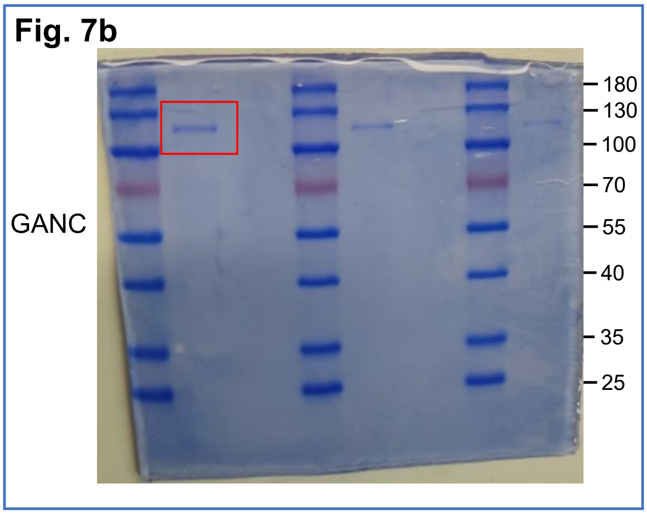
Fig. 7b
GANC
180
130
100
70
55
40
35
25

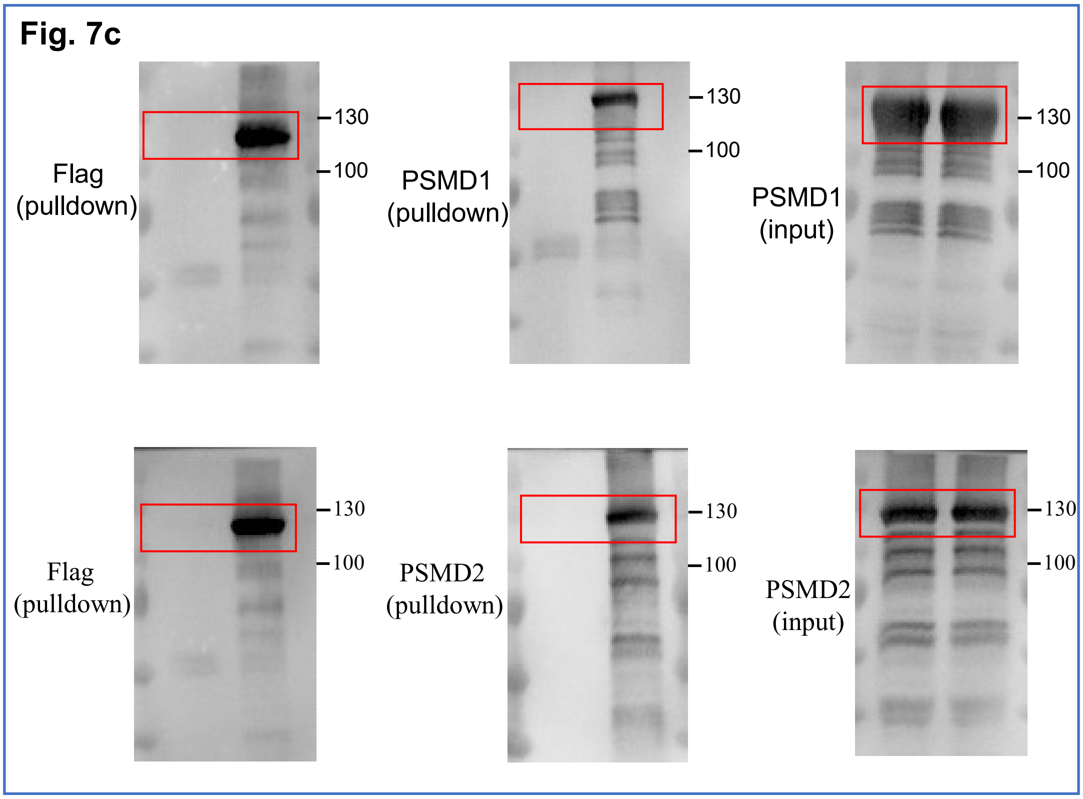
Fig. 7c
Flag (pulldown)
130
100
PSMD1 (pulldown)
130
100
PSMD1 (input)
130
100
Flag (pulldown)
130
100
PSMD2 (pulldown)
130
100
PSMD2 (input)
130
100

Fig. 7e

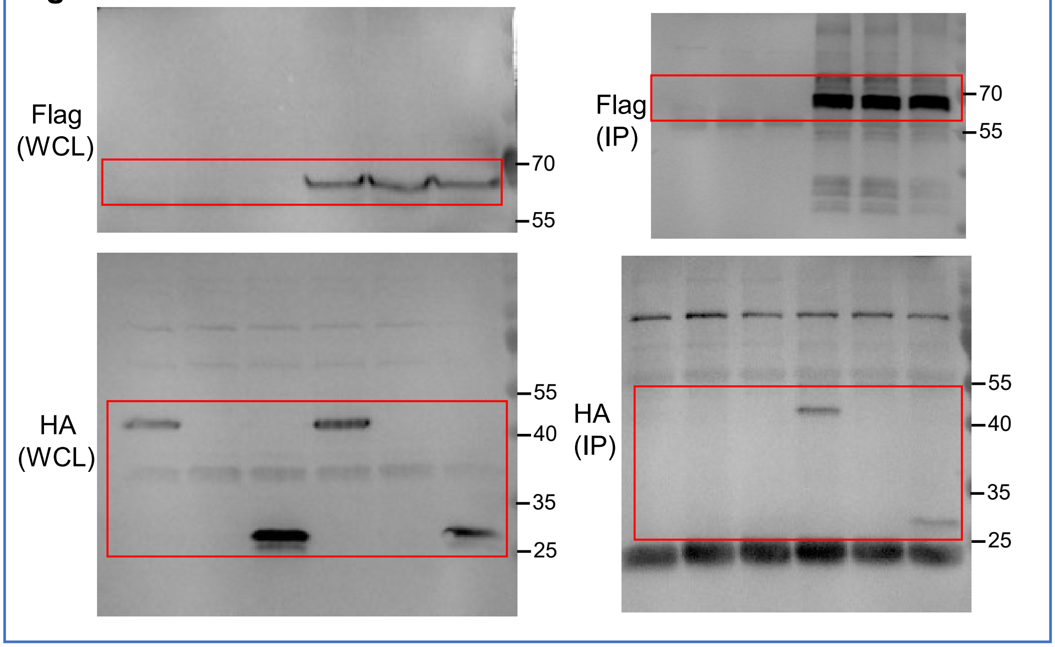
Flag (WCL)
70
55
Flag (IP)
70
55
HA (WCL)
55
40
35
25
HA (IP)
55
40
35
25

Fig. 7f

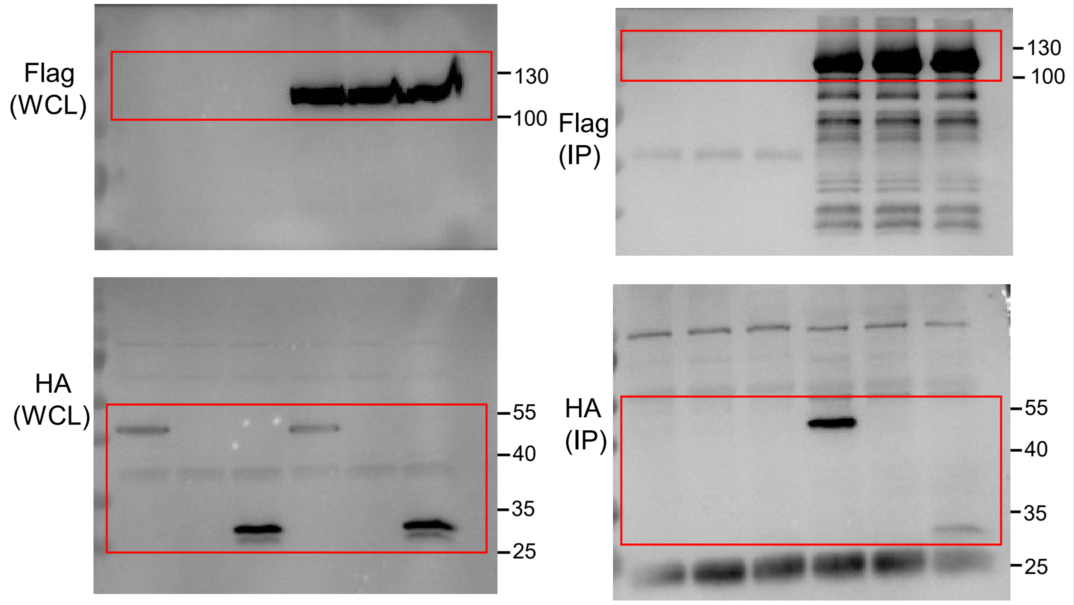
Flag (WCL)
130
100
Flag (IP)
130
100
HA (WCL)
55
40
35
25
HA (IP)
55
40
35
25

Fig. 7h

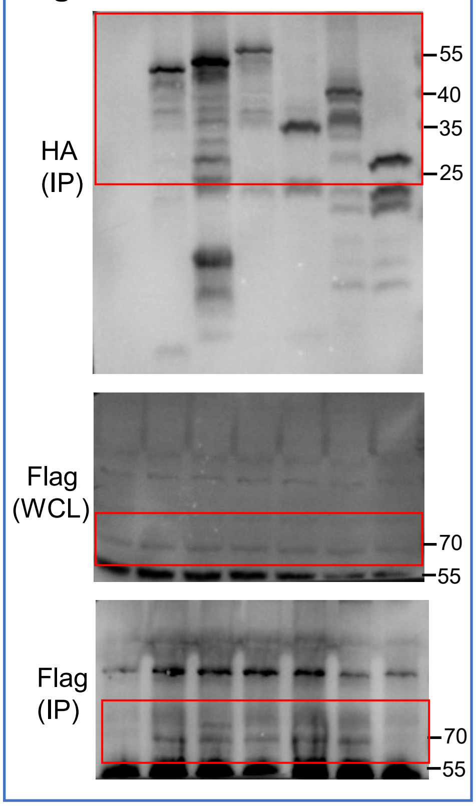

Fig. 7i

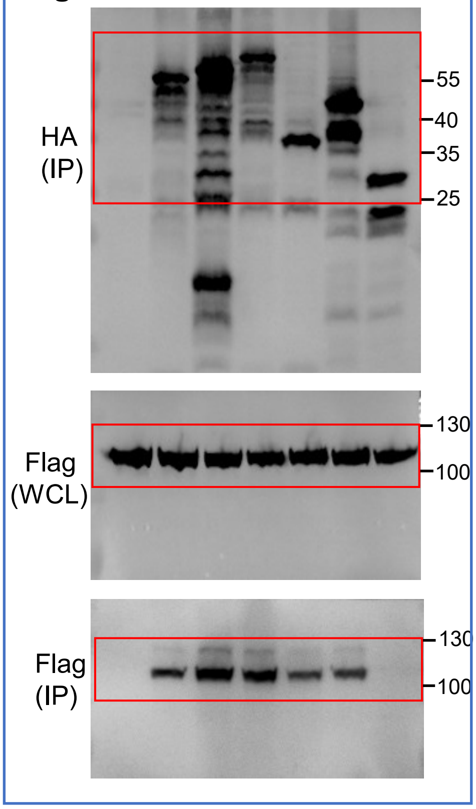

Fig. 7j

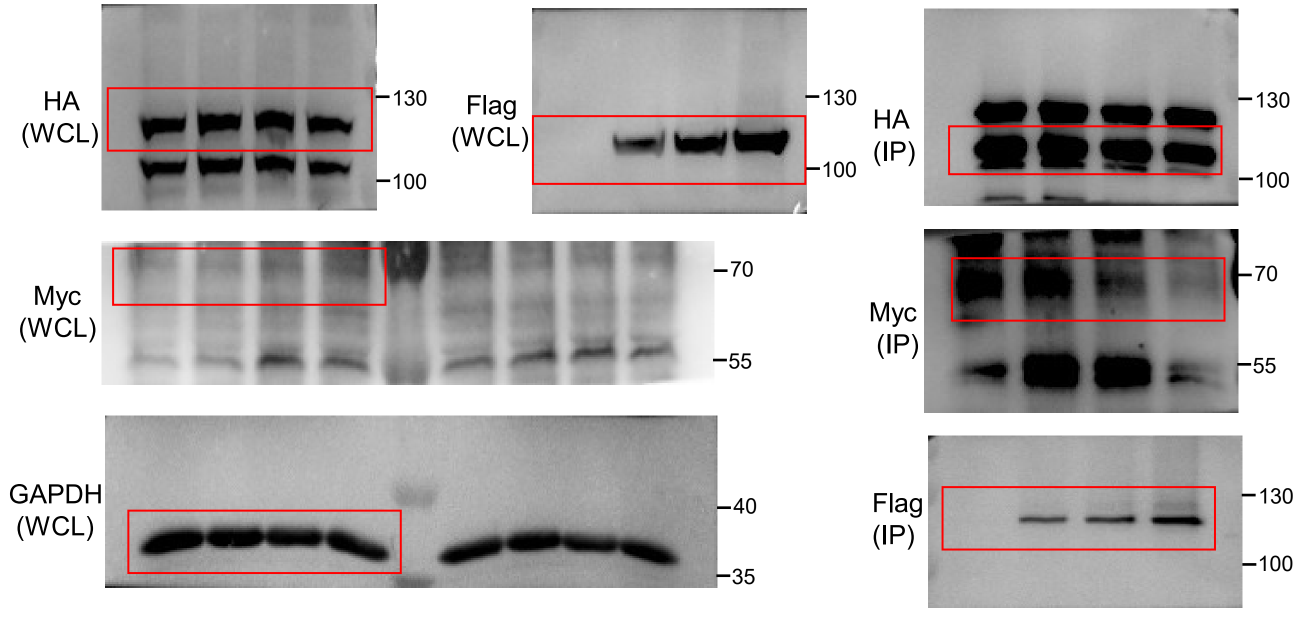

Fig. 7k

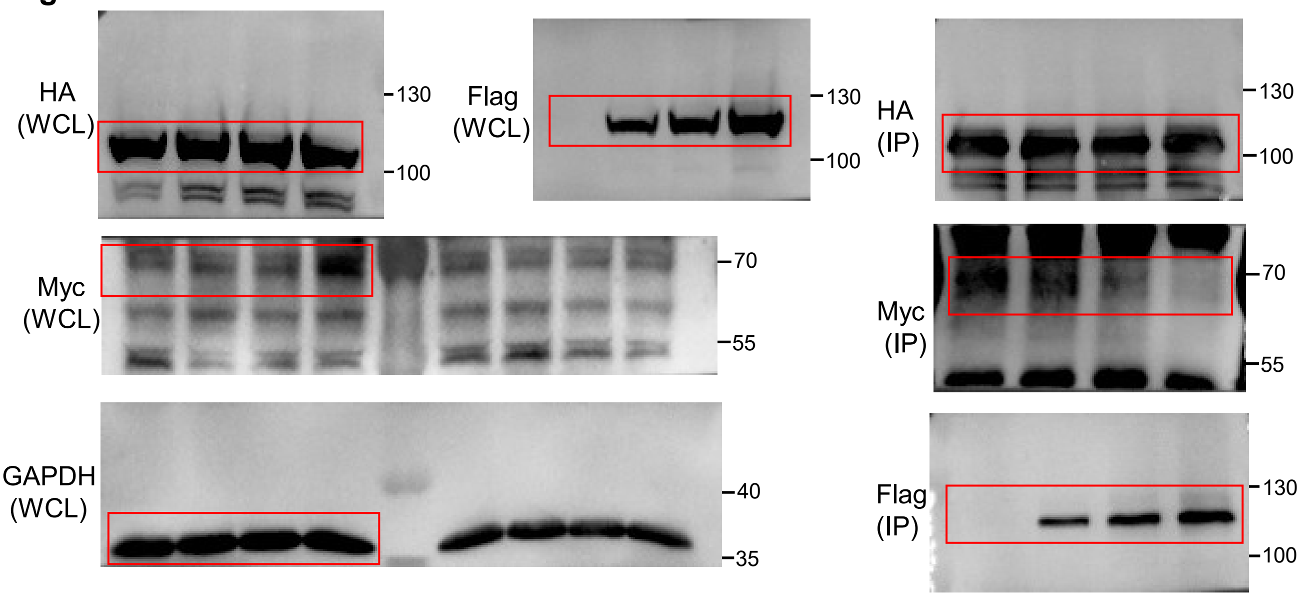

Fig. 7l

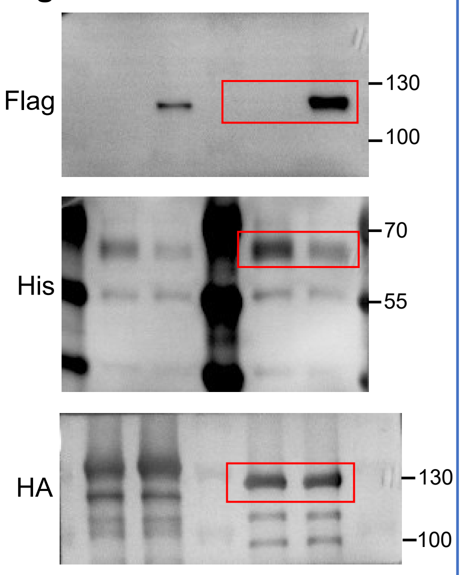

Fig. 7m

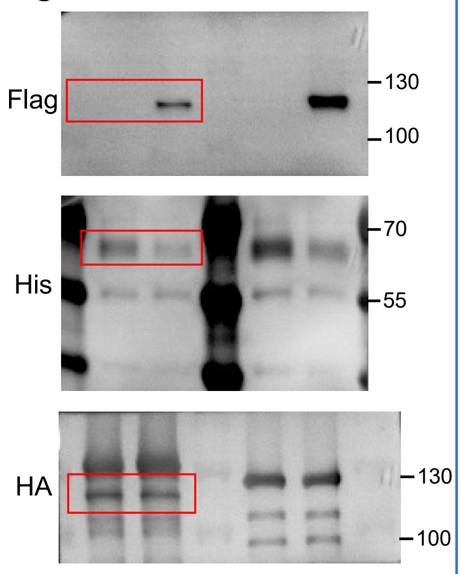

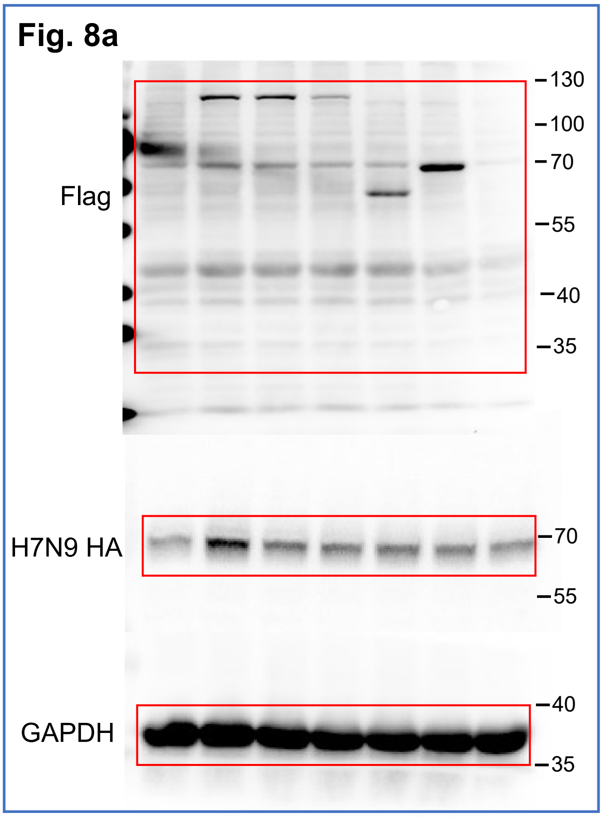

Fig. 8a

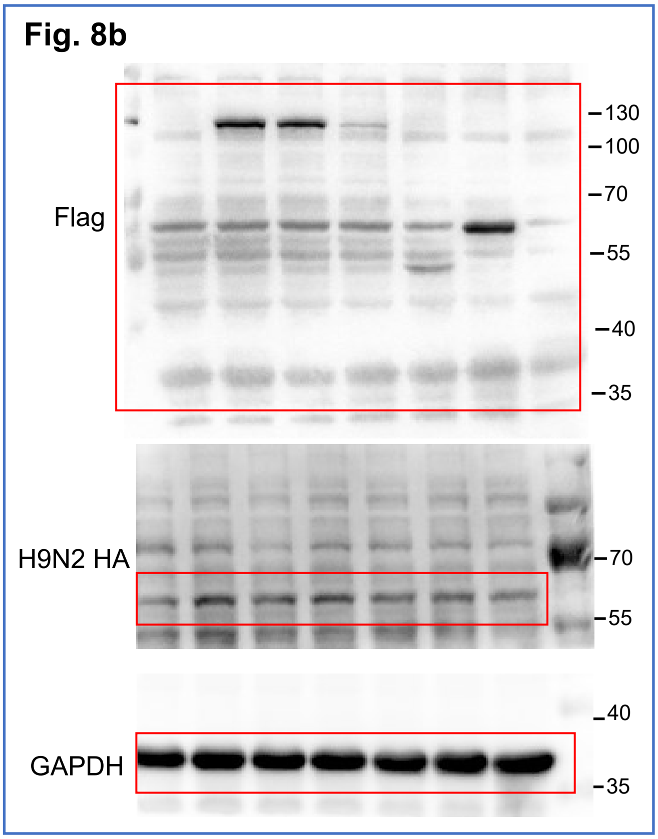

Fig. 8b

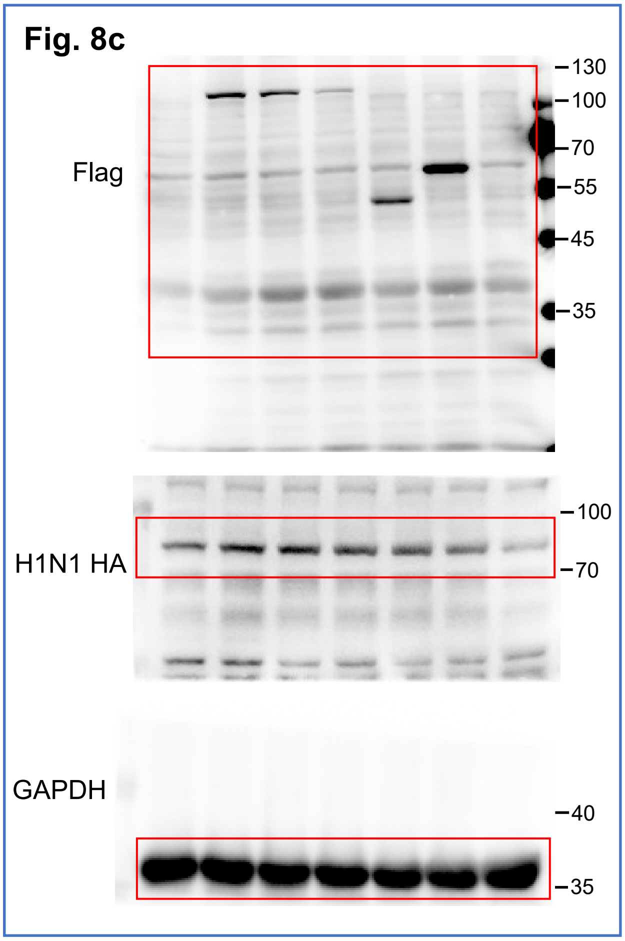

Fig. 8c

Fig. S2c

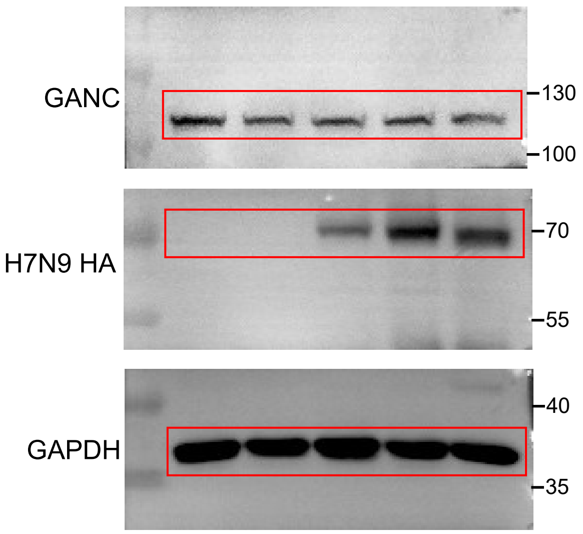

Fig. S2f

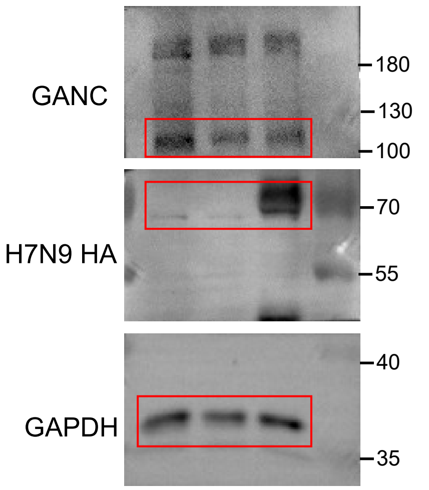

Fig. S6a

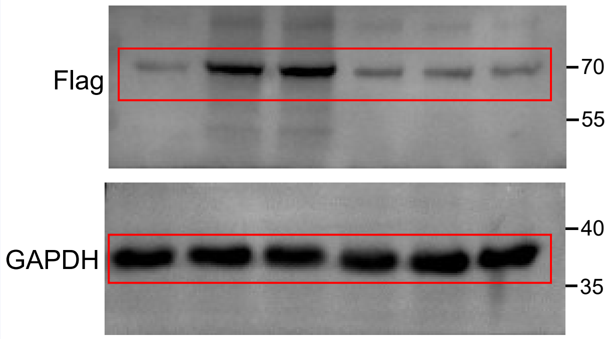

Fig. S6b

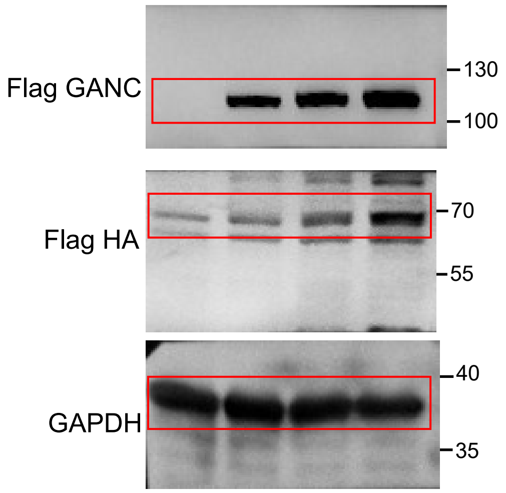

Fig. S6c

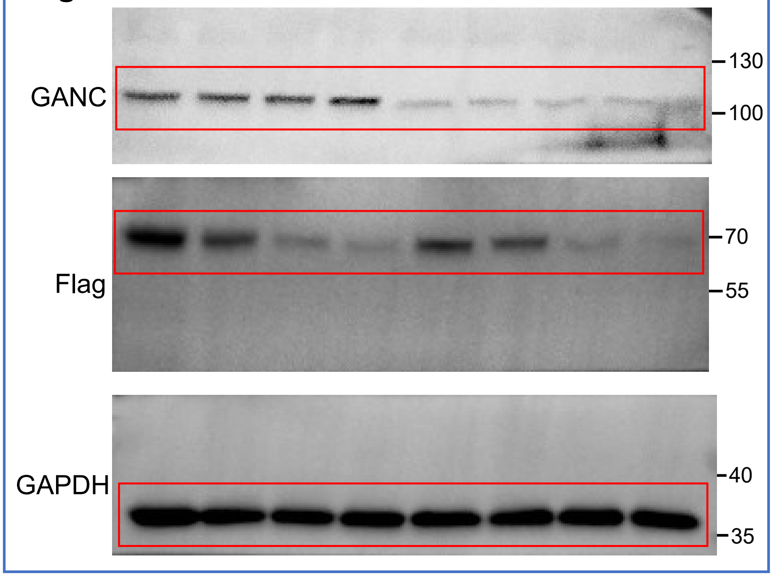

Fig. S6d

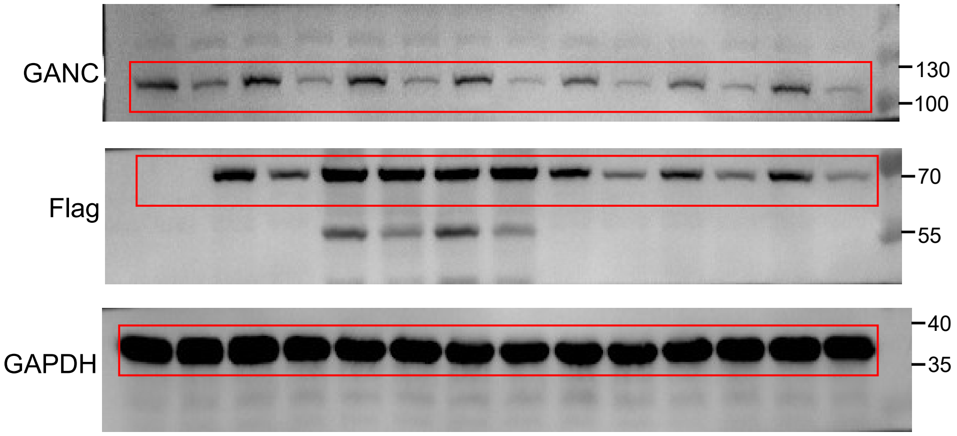

Fig. S6e

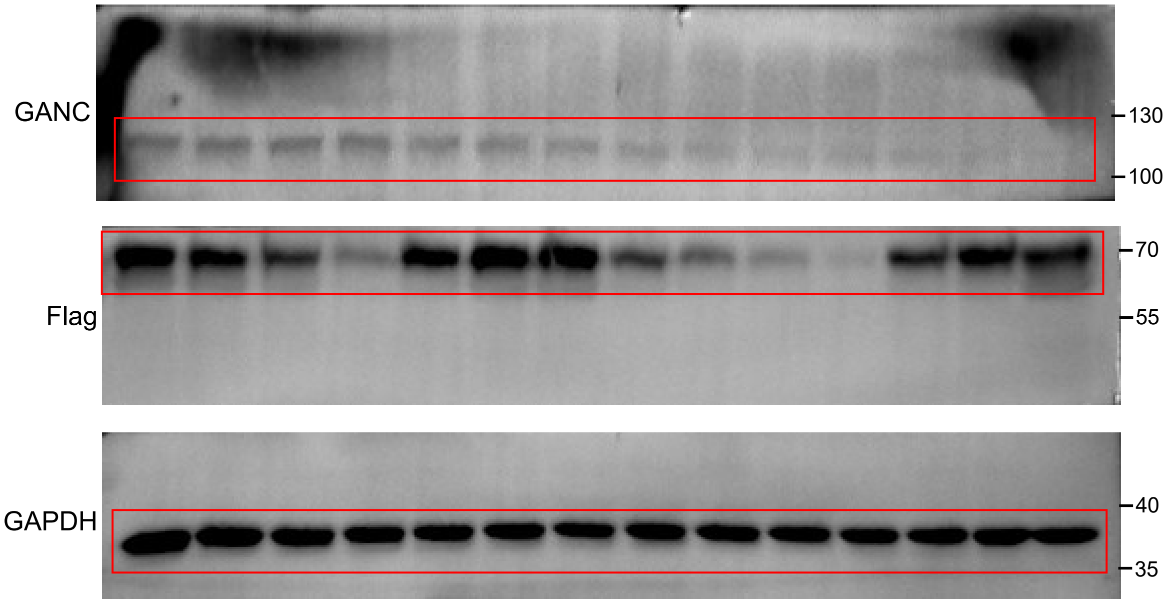

Fig. S7a

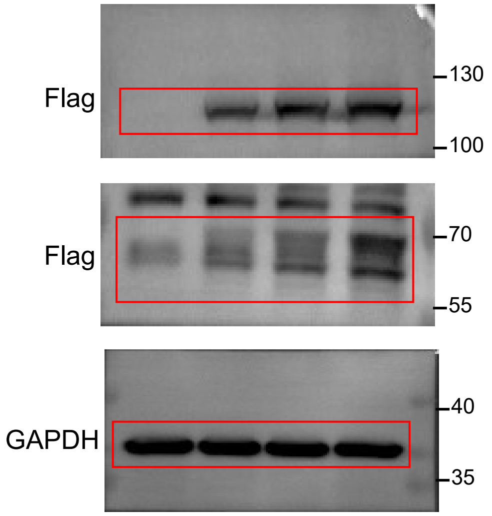

Fig. S7b

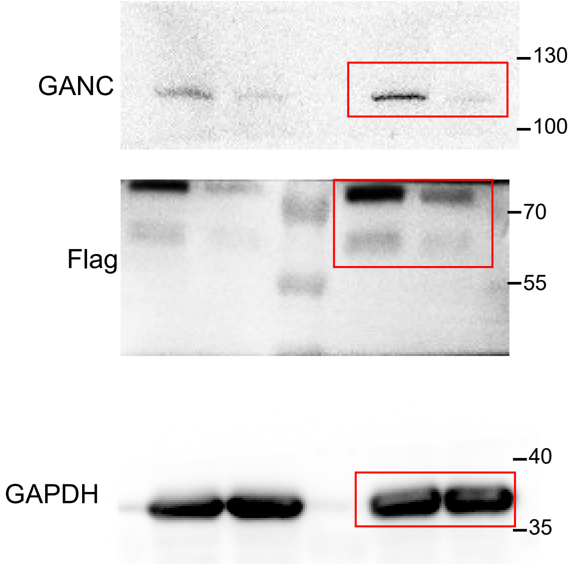

Fig. S8a

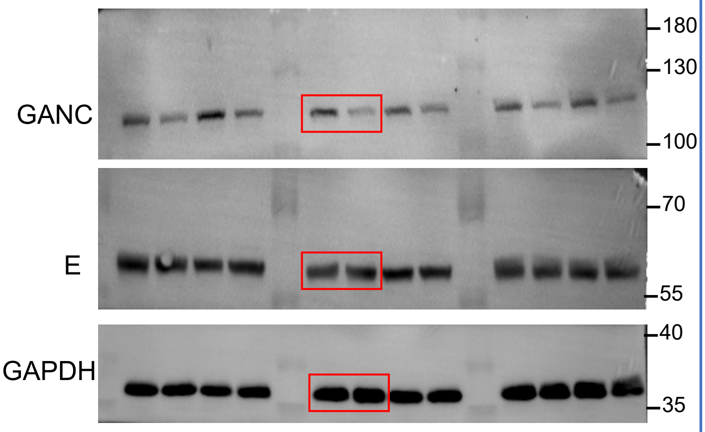

Fig. S9a

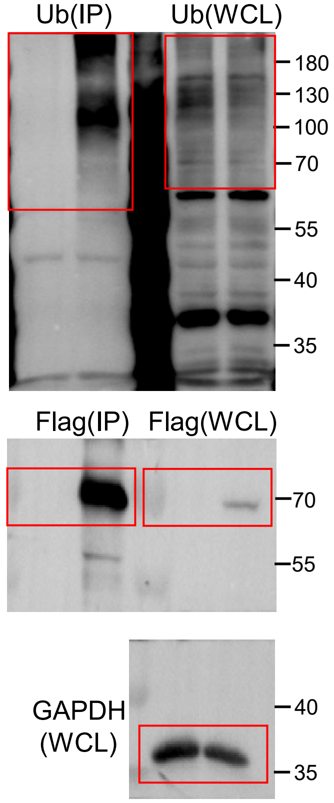

Fig. S9b

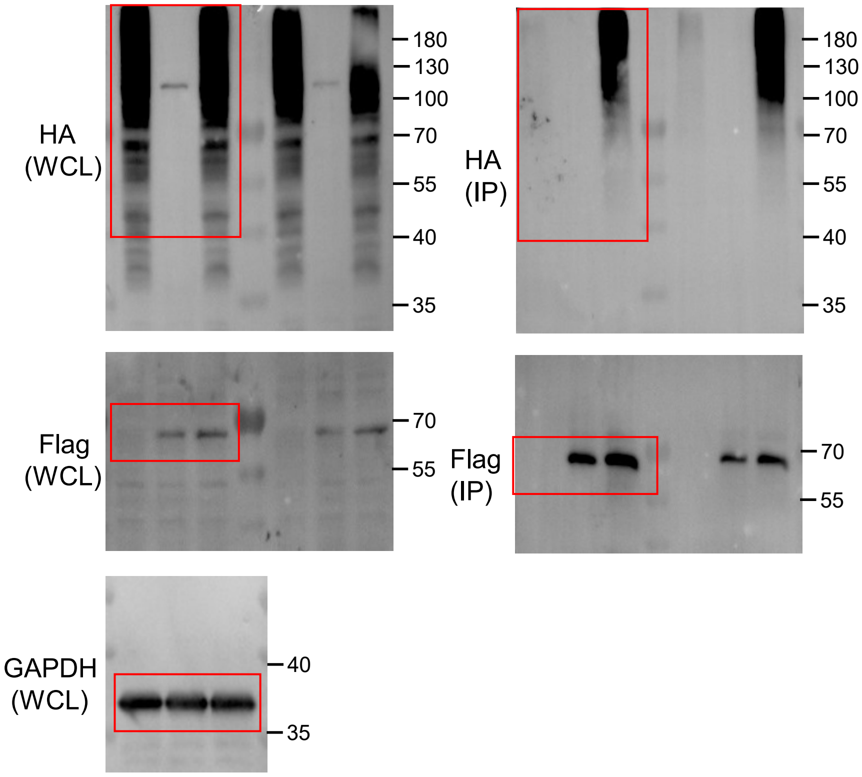

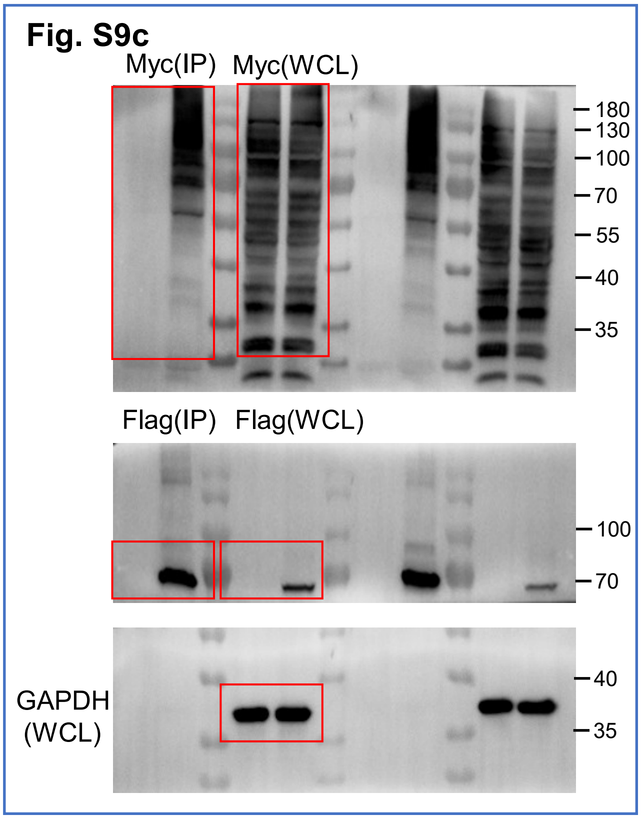
Fig. S9c
Myc(IP)
Myc(WCL)
180
130
100
70
55
40
35
Flag(IP)
Flag(WCL)
100
70
GAPDH
(WCL)
40
35

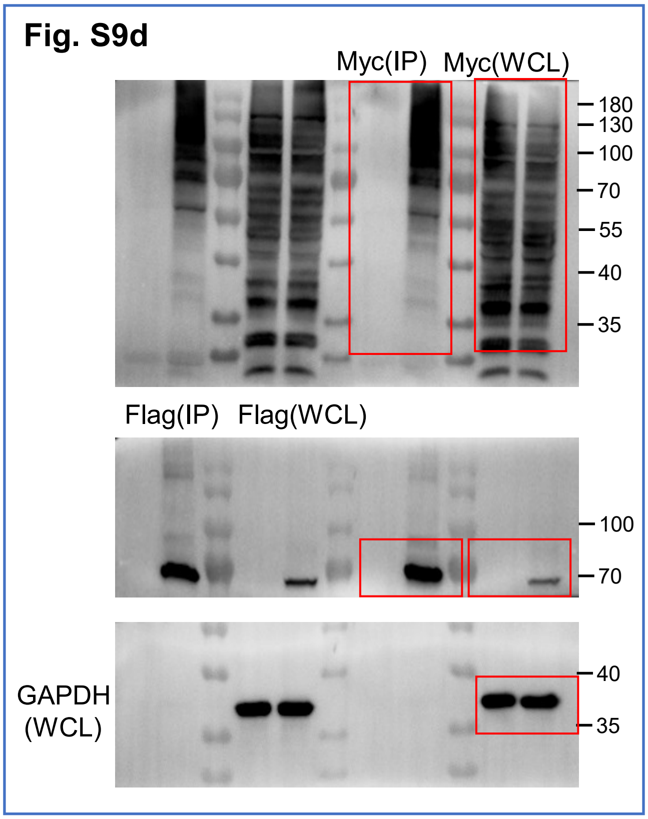
Fig. S9d
Myc(IP)
Myc(WCL)
180
130
100
70
55
40
35
Flag(IP)
Flag(WCL)
100
70
GAPDH
(WCL)
40
35

Fig. S10a

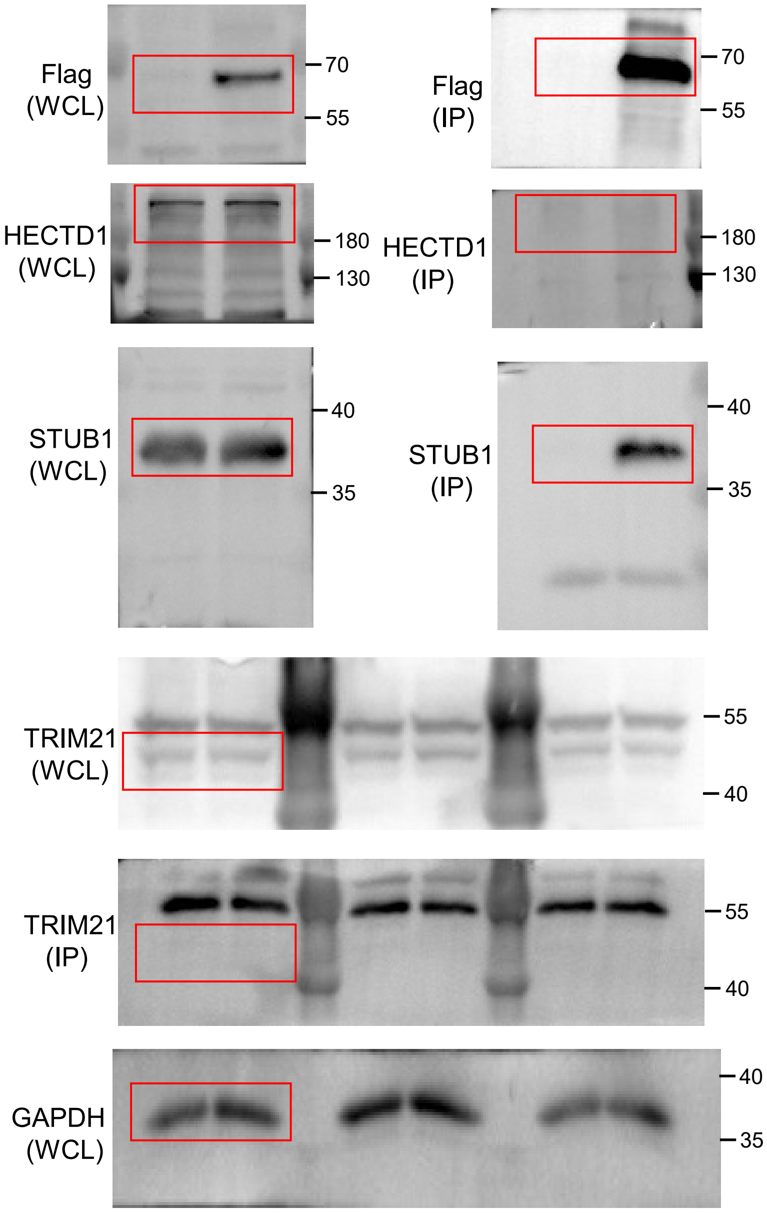

Fig. S10b

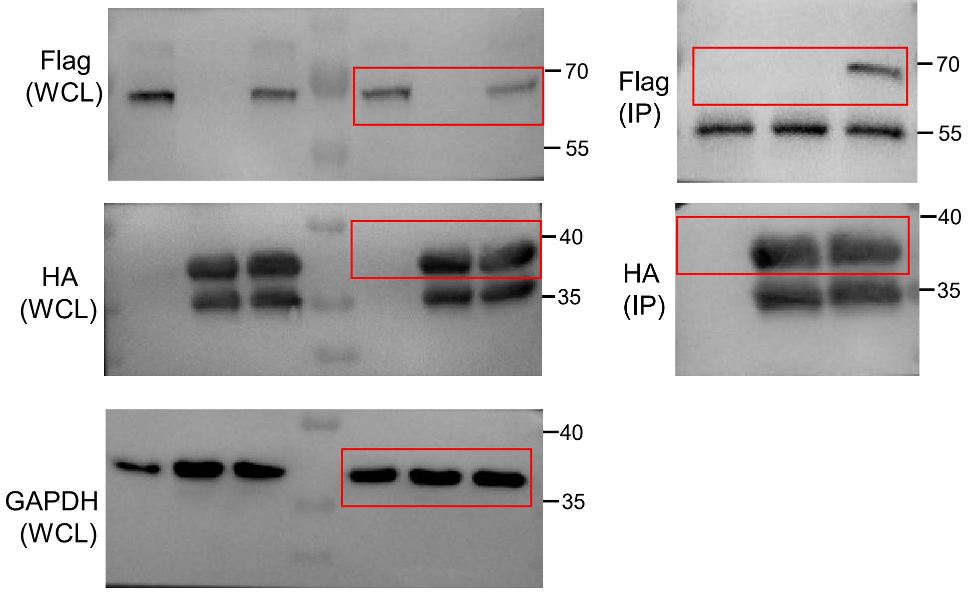

Fig. S10c

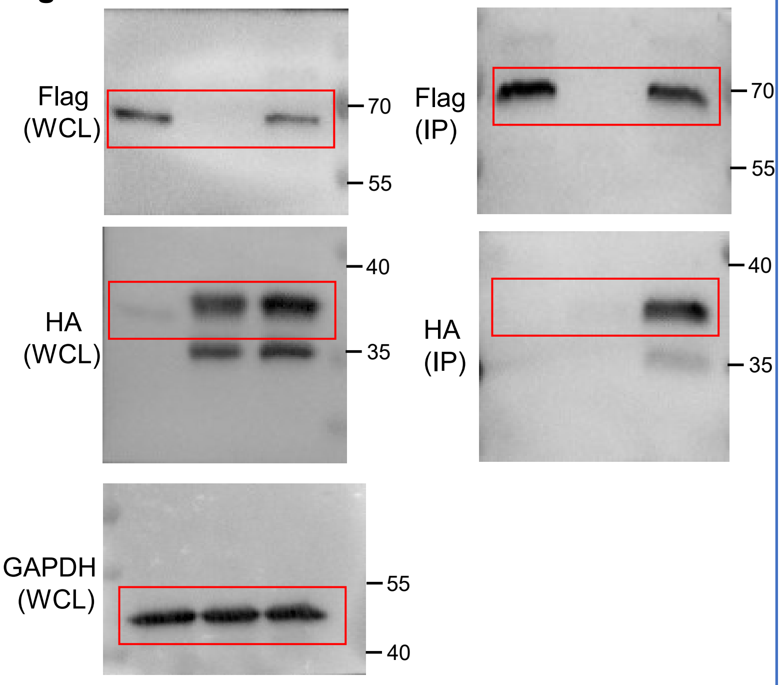

Fig. S10e

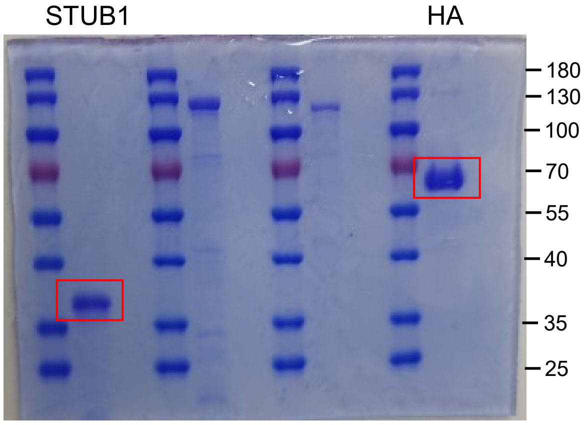

Fig. S10f

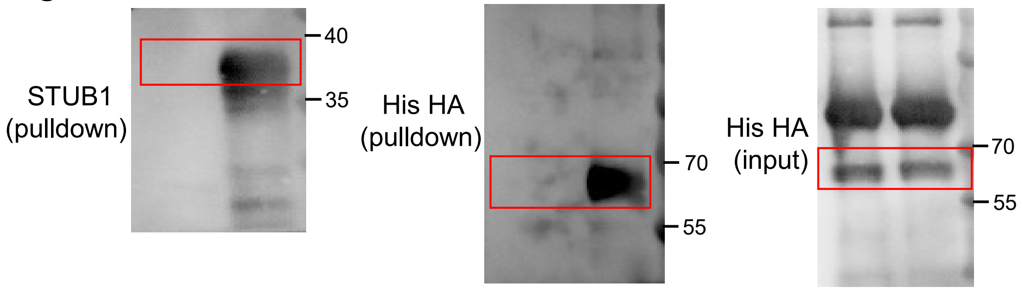

Fig. S11a

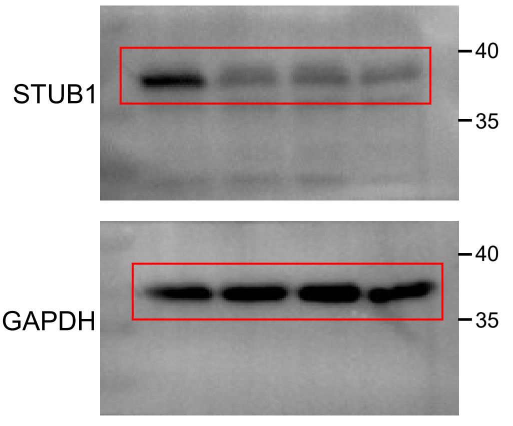

Fig. S11b

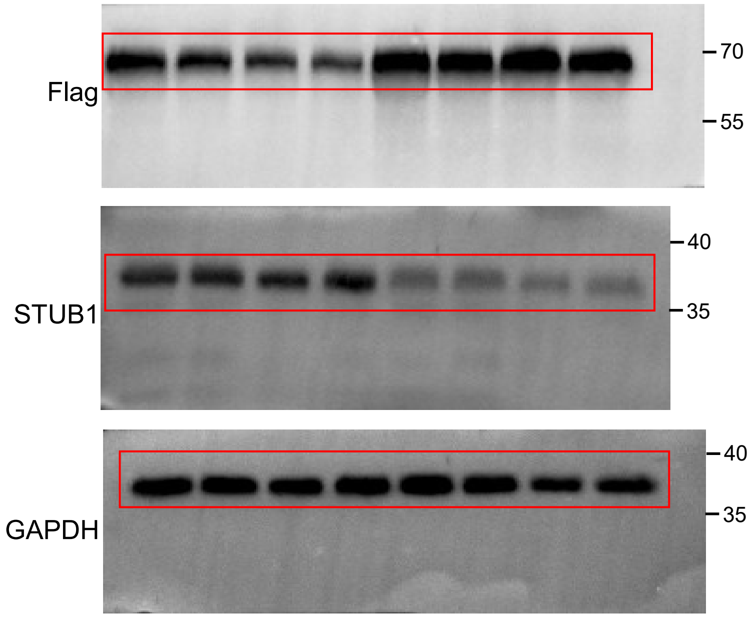

Fig. S11c

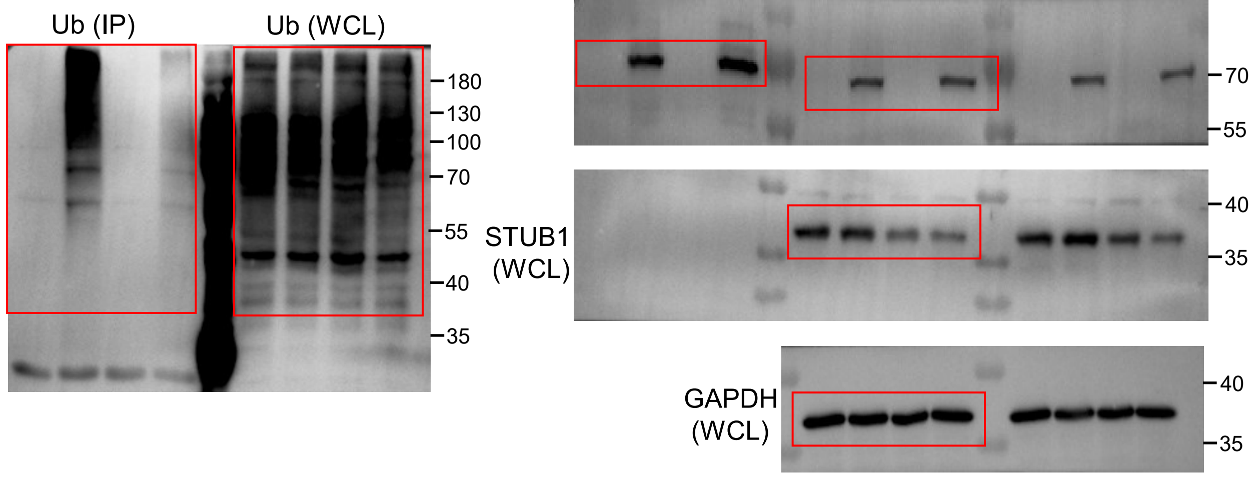

Fig. S11d

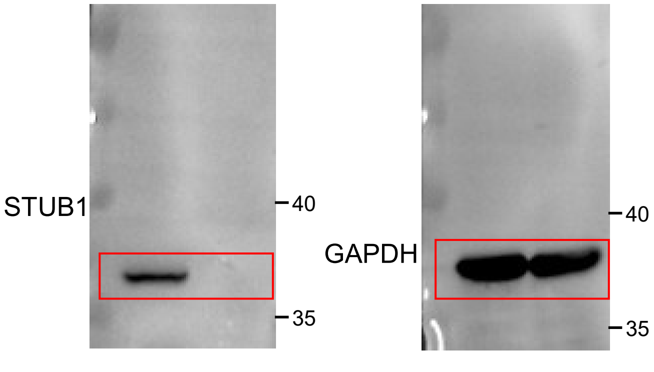

Fig. S11e

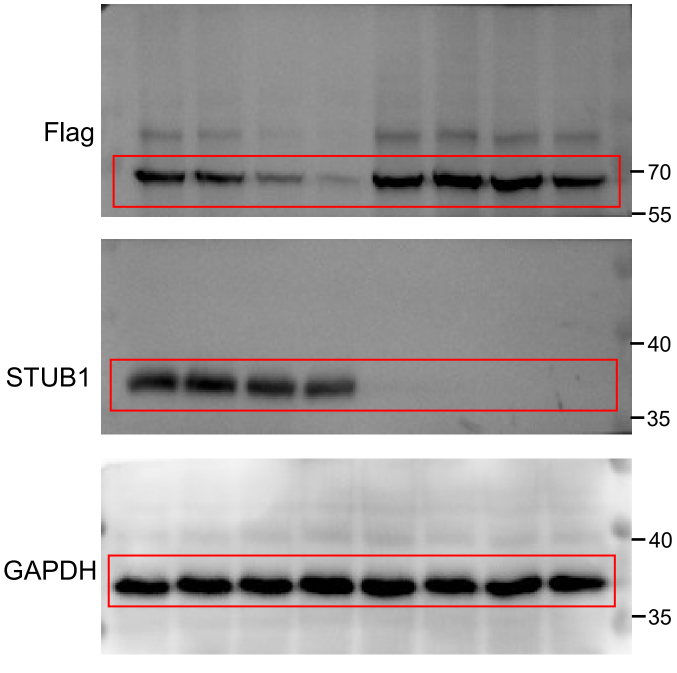

Fig. S11f

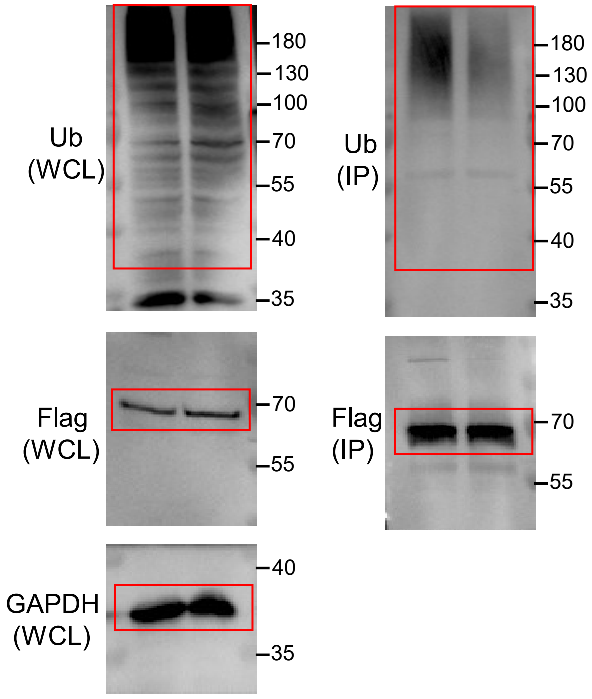

Fig. S11g

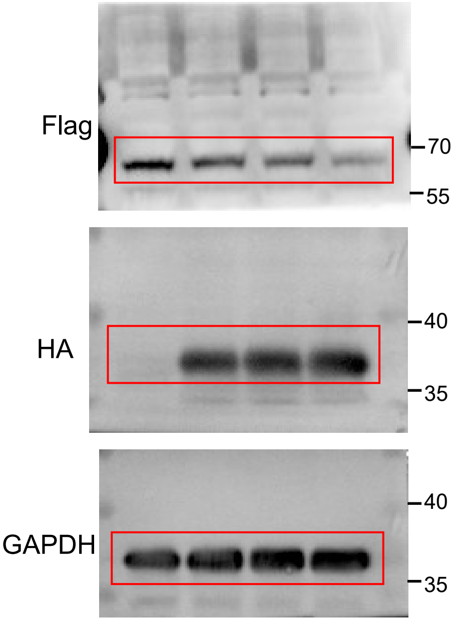

Fig. S11h

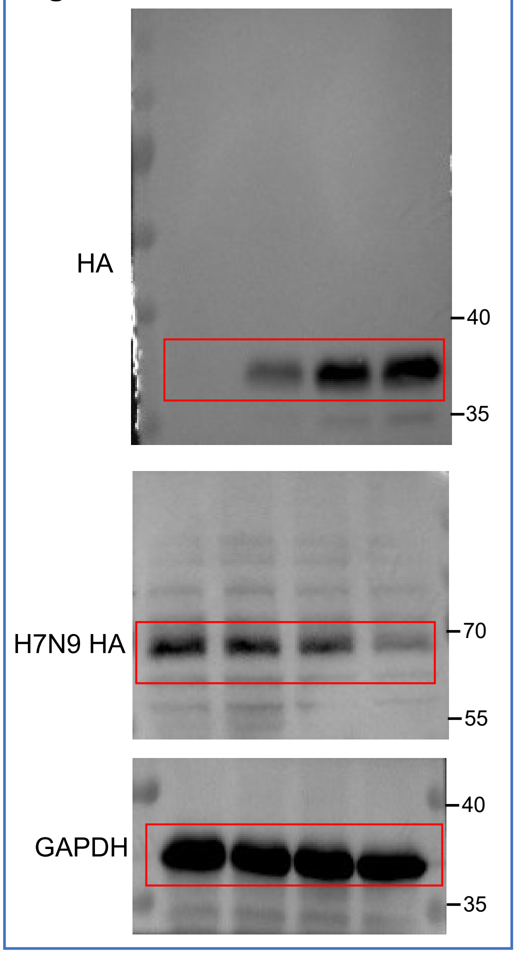

Fig. S11i

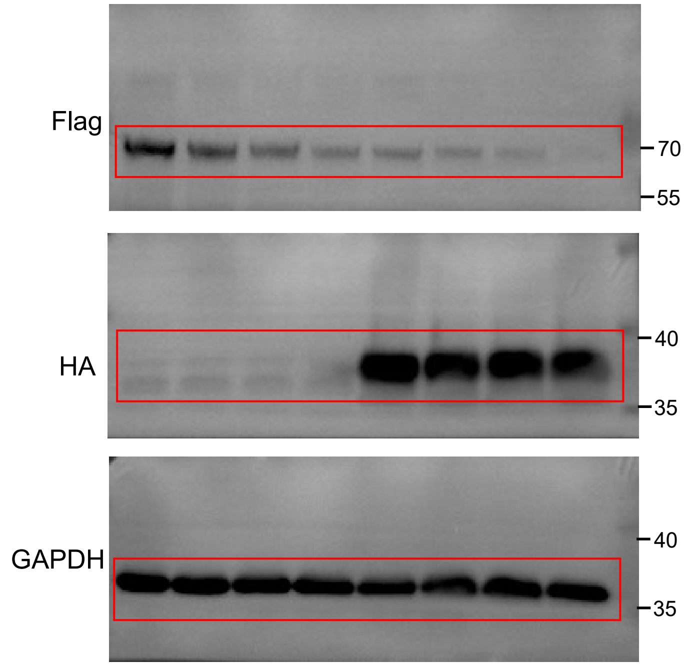

Fig. S11j

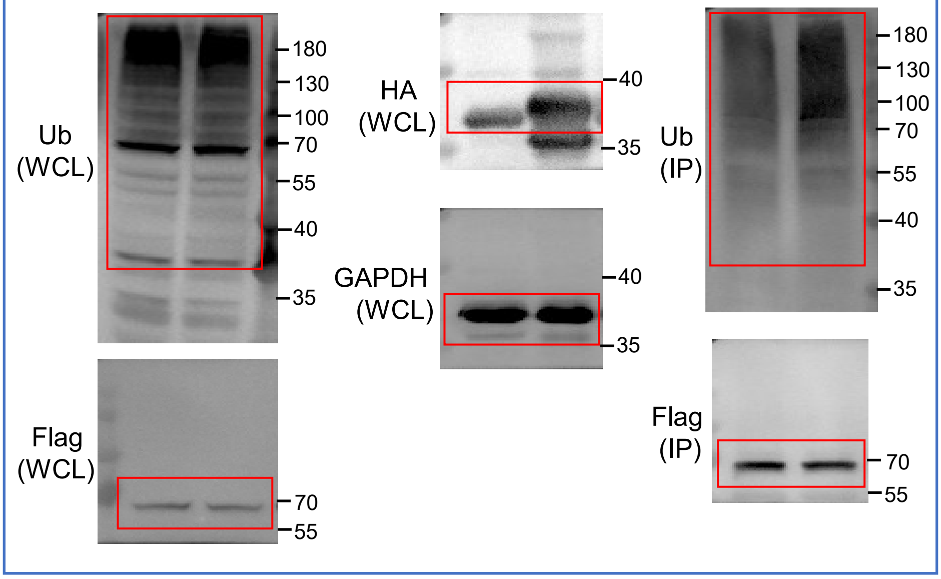

Fig. S11k

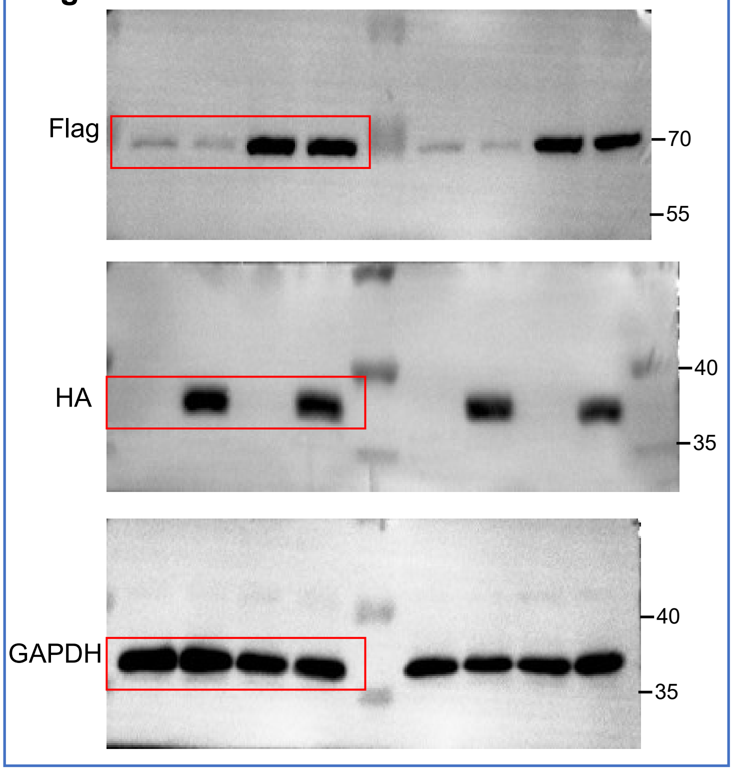

Fig. S11l

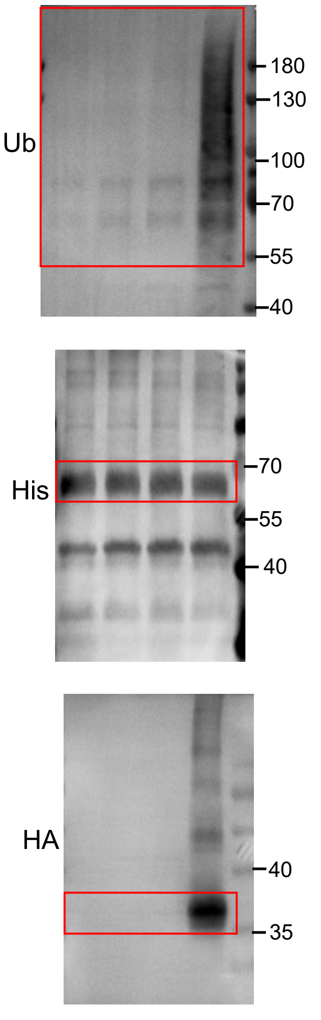

Fig. S11m

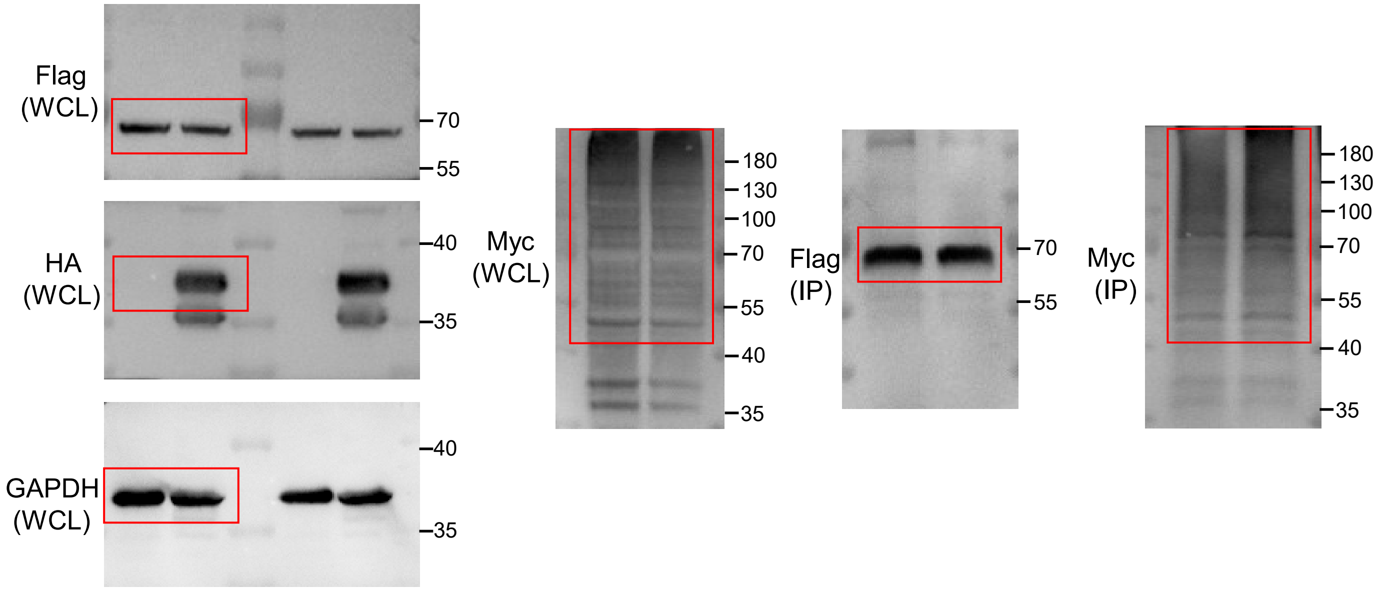

Fig. S11n

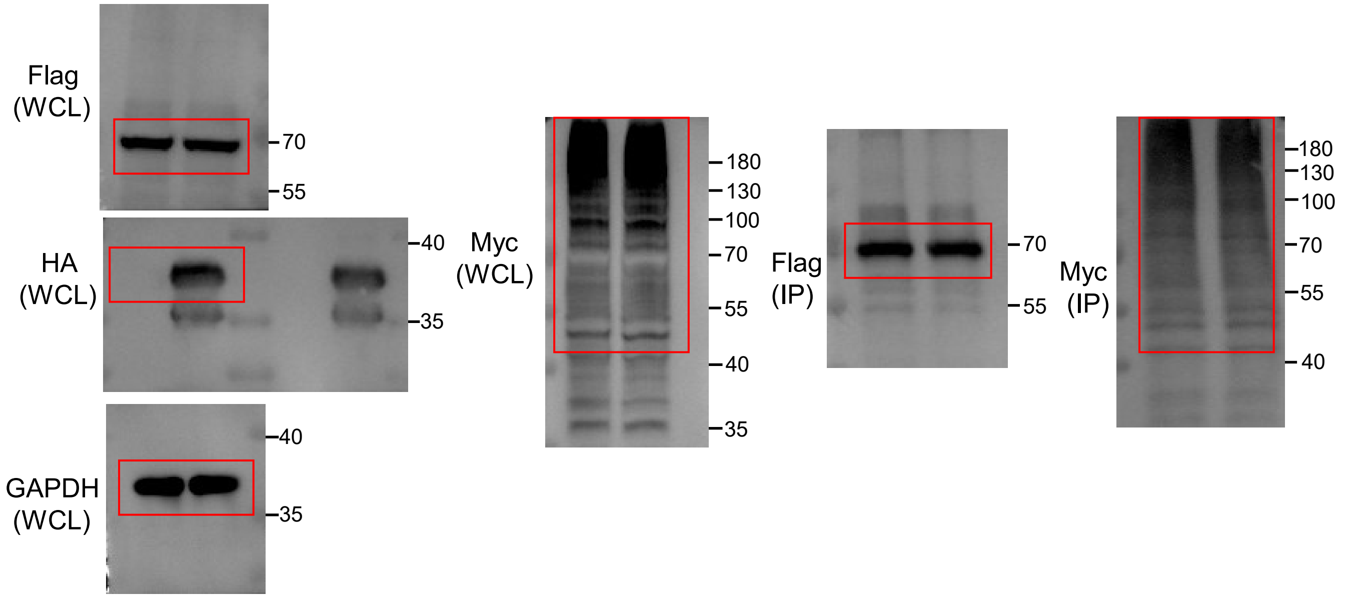

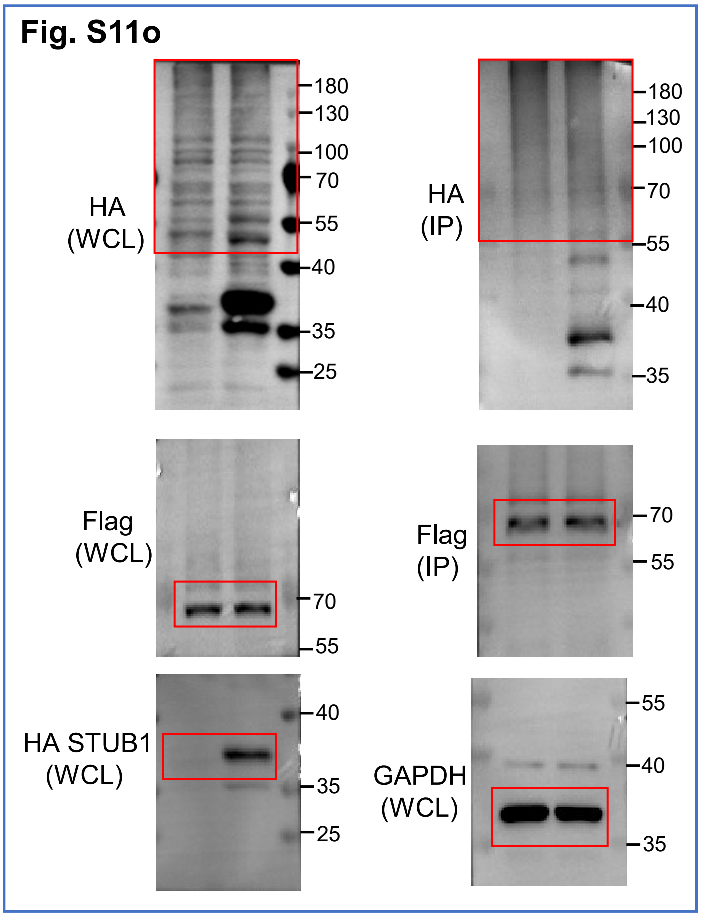
Fig. S11o
HA
(WCL)
180
130
100
70
55
40
35
25
HA
(IP)
180
130
100
70
55
40
35
Flag
(WCL)
70
55
Flag
(IP)
70
55
HA STUB1
(WCL)
40
35
25
GAPDH
(WCL)
55
40
35

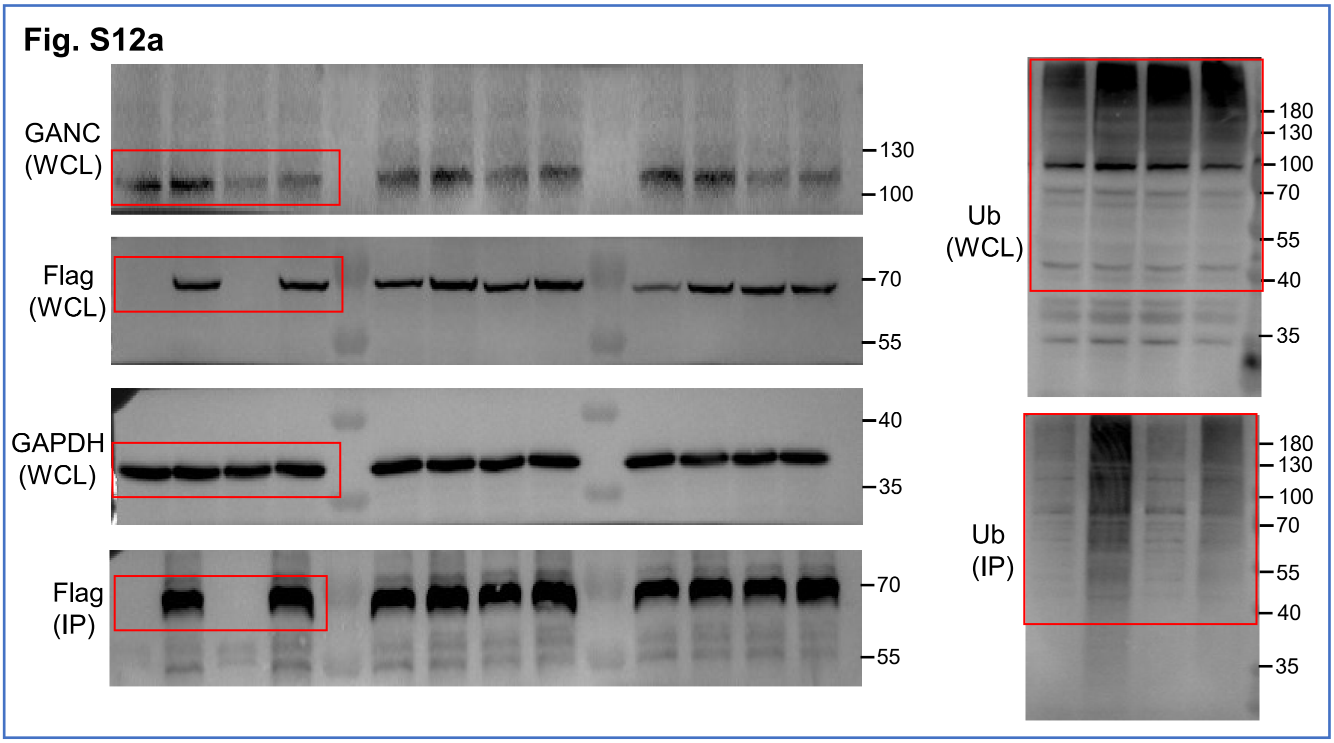
Fig. S12a
GANC
(WCL)
130
100
Flag
(WCL)
70
55
GAPDH
(WCL)
40
35
Flag
(IP)
70
55
Ub
(WCL)
180
130
100
70
55
40
35
Ub
(IP)
180
130
100
70
55
40
35

Fig. S12b

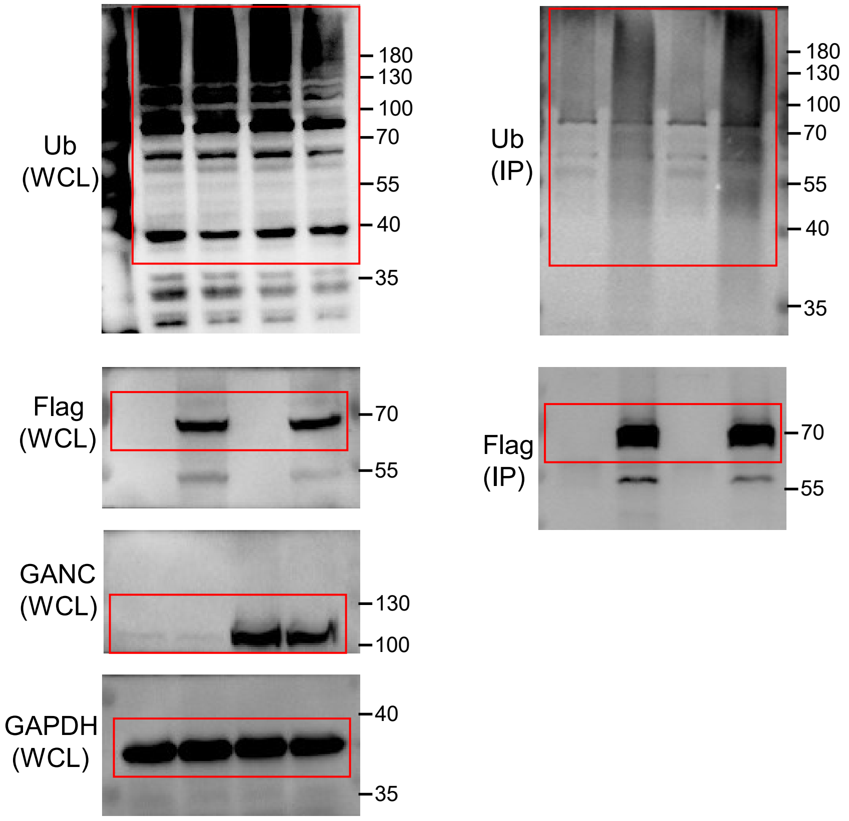

Fig. S13a

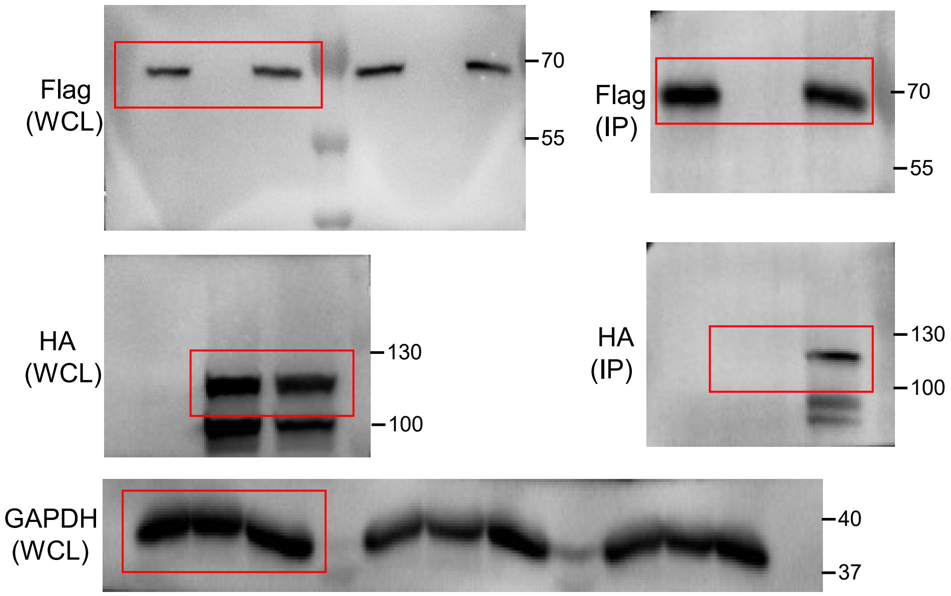

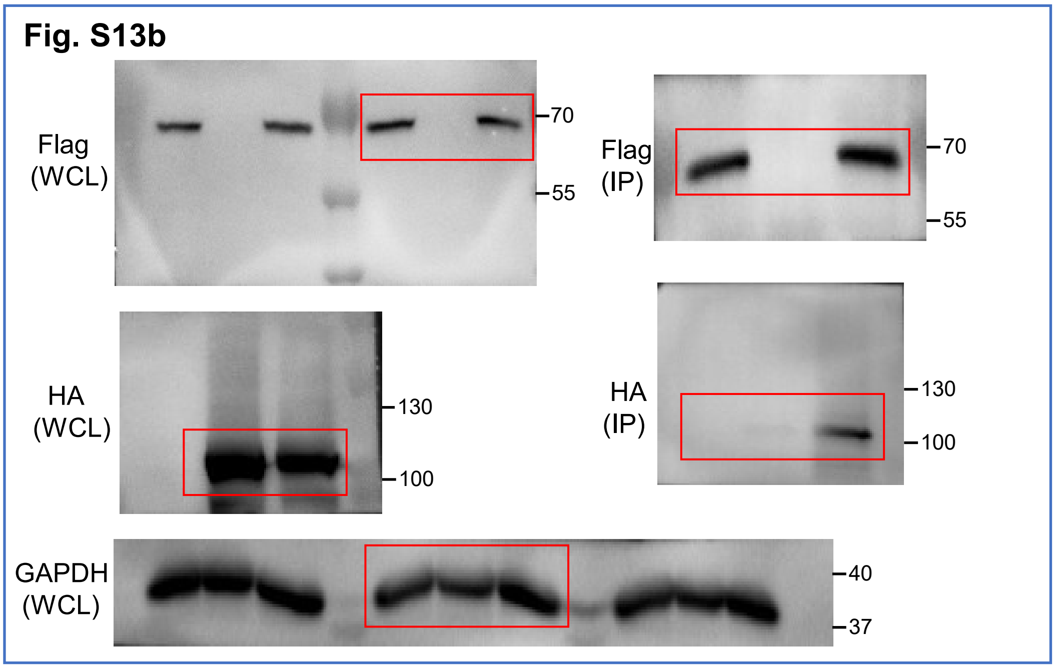
Fig. S13b
Flag
(WCL)
70
55
Flag
(IP)
70
55
HA
(WCL)
130
100
HA
(IP)
130
100
GAPDH
(WCL)
40
37

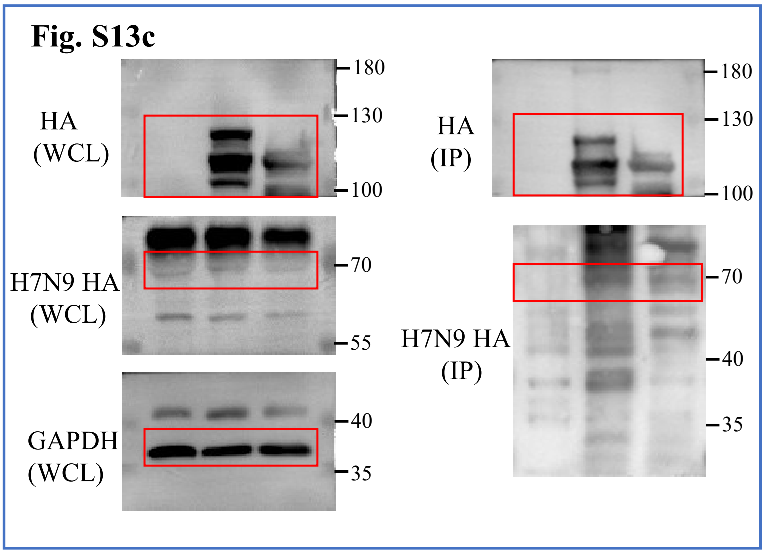
Fig. S13c
HA
(WCL)
180
130
100
HA
(IP)
180
130
100
H7N9 HA
(WCL)
70
55
H7N9 HA
(IP)
70
40
35
GAPDH
(WCL)
40
35

Fig. S14a

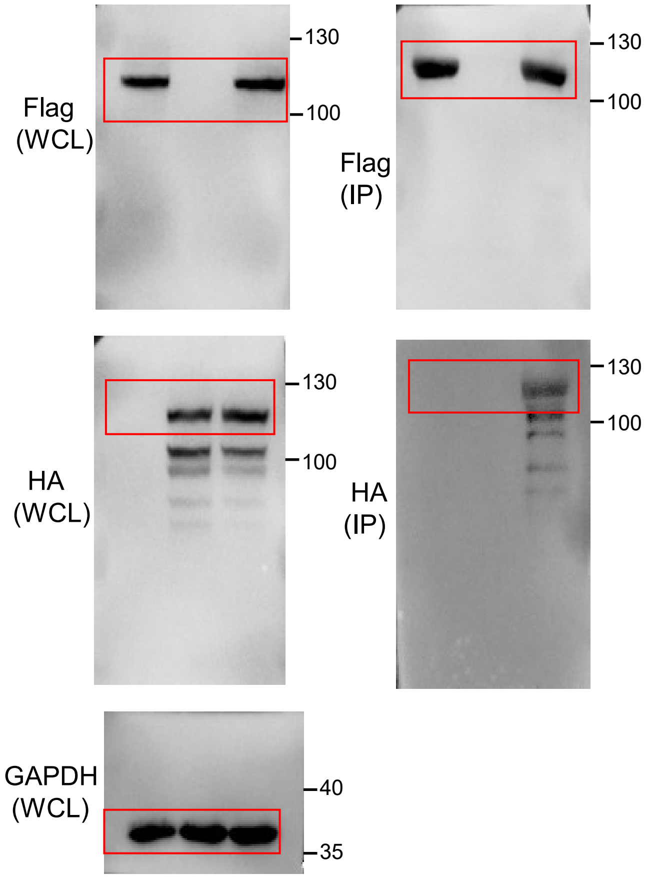

Fig. S14b

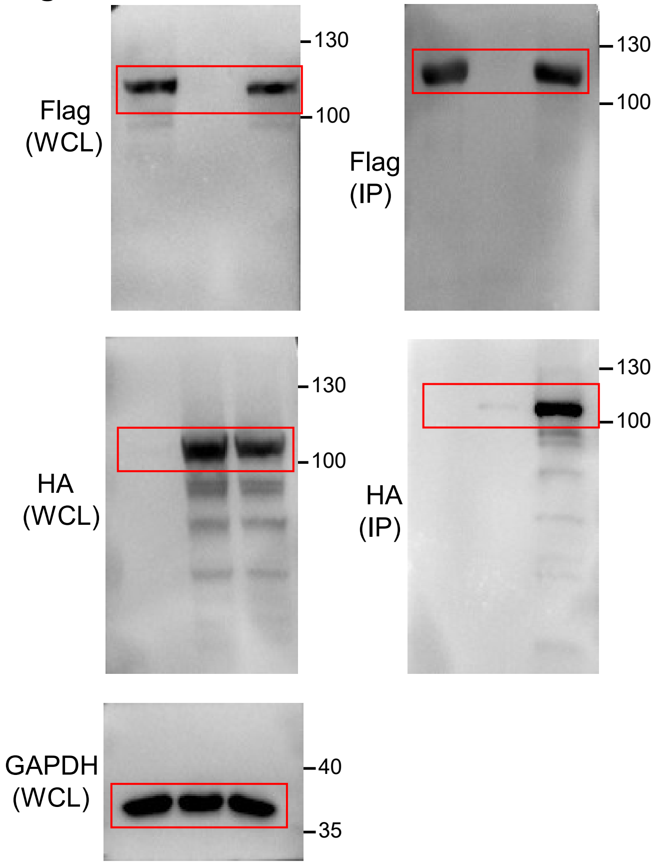

Fig. S14c

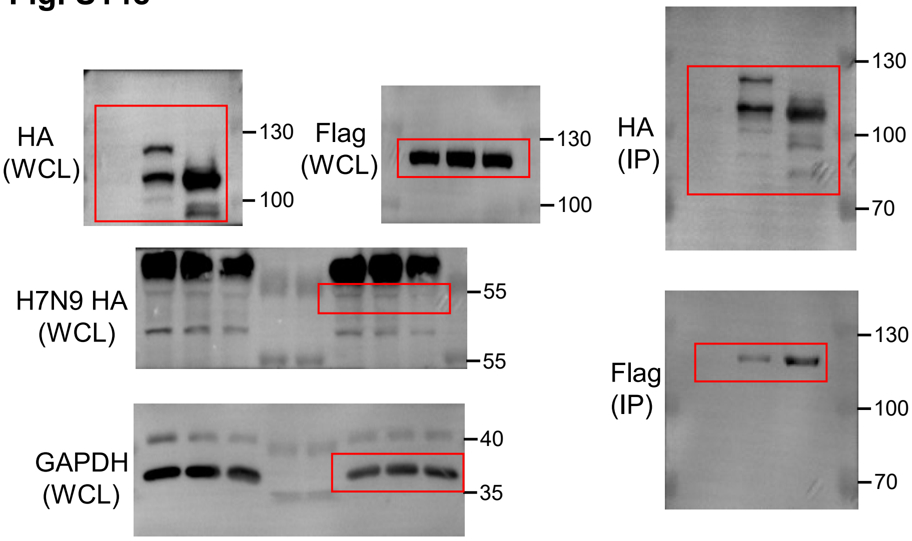

Fig. S15a

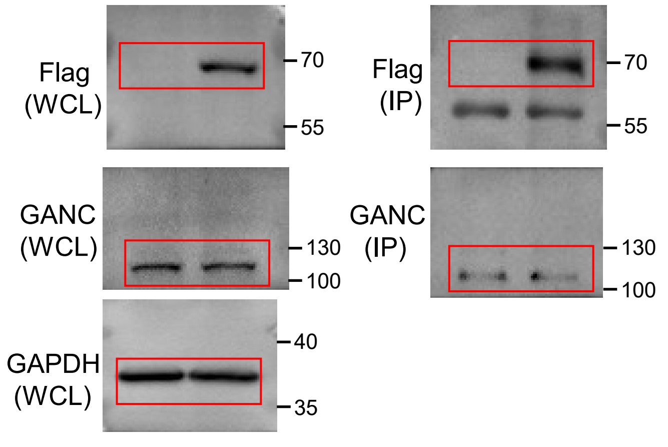

Fig. S15b

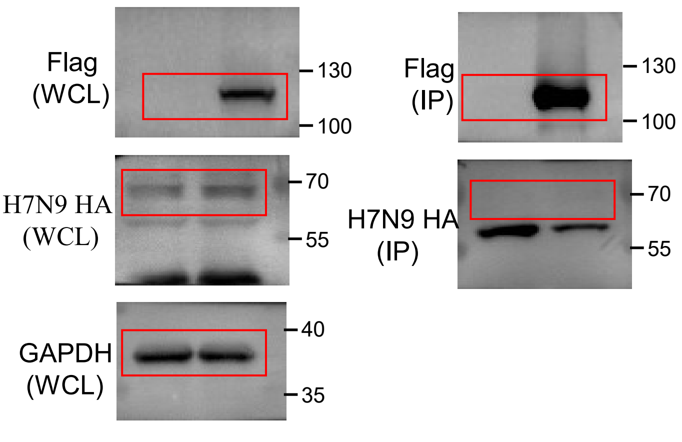

Supplement: Supplementary file 2 — Uncropped Western Blots [file 41392_2025_2227_MOESM2_ESM.pdf]
